# TUFT1 stabilizes TGF-β receptor II protein and facilitates activation of hepatic stellate cells into metastasis-promoting myofibroblasts

**DOI:** 10.1038/s41418-026-01664-2

**Published:** 2026-01-28

**Authors:** Yue Li, Yu Shi, Yaxin Fu, Lianying Jiao, Hanqi Li, Lu Shao, Xuelian Xiao, Ningling Kang, Liankang Sun, Kangsheng Tu

**Affiliations:** 1 Department of Hepatobiliary Surgery, The First Affiliated Hospital of Xi’an Jiaotong University, Xi’an, China; 2 Department of Oncology, The First Affiliated Hospital of Xi’an Jiaotong University, Xi’an, China; 3 Department of Biochemistry and Molecular Biology, School of Basic Medical Sciences, Xi’an Jiaotong University Health Science Center, Xi’an, China; 4 Tumor Microenvironment and Metastasis, the Hormel Institute, University of Minnesota, Austin, MN, USA; 5 These authors contributed equally: Yue Li, Yu Shi

## Abstract

Cancer-associated fibroblasts (CAFs) transdifferentiated from hepatic stellate cells (HSCs) are a critical determinant of liver metastasis of colorectal cancer (CRC). However, the mechanisms behind transforming growth factor β (TGF-β)-stimulated activation of HSCs into CAFs remain poorly understood. Immunoprecipitation coupled with mass spectrometry identified tuftelin 1 (TUFT1) as a novel TGF-β receptor II (TβRII) binding protein in primary human HSCs and immortalized LX2 cells. TUFT1 interacts with TβRII via its fragments (amino acids 1–86, 87–157), protecting TβRII from lysosomal degradation to facilitate TGF-β signaling and myofibroblastic activation of HSCs. Mechanistically, TUFT1 competes with caveolin-1 for TβRII binding, retrieving TβRII from the lipid rafts/caveolae-mediated degradation pathway and sorting it into the endosome-mediated trafficking and signaling pathway. Clinically, TUFT1 expression was confirmed in the CAFs of patient-derived colorectal cancer liver metastasis (CRCLM) tissues. Both protein and transcript analyses revealed higher TUFT1 expression in the CAFs of CRCLM than in HSCs. Furthermore, bulk RNA sequencing indicated that knocking down TUFT1 altered the TGF-β transcriptome of HSCs and suppressed HSC expression of tumor-promoting factors. In HSC/CRC co-implantation and portal vein tumor injection mouse models, targeting TUFT1 of HSCs inhibited HSC activation and restricted CRC growth in both subcutaneous and hepatic sites. Taken together, our findings uncover the novel function of TUFT1 in the hepatic tumor microenvironment, highlighting its role as a critical regulator of HSC activation and the pro-metastatic hepatic niche via promoting TβRII protein stability. Targeting TUFT1 in HSCs presents a promising therapeutic approach for combating CRCLM.

## INTRODUCTION

Colorectal cancer (CRC) is one of the most common and fatal malignancies in the world [1]. Metastasis develops in over 50% of CRC patients, with the liver as its primary target [2]. Clinically, liver metastasis is the predominant cause of death in patients with CRC [3]. Despite significant advances in treatment, clinical outcomes for CRC patients with liver metastasis remain unfavorable [4]. Therefore, understanding the molecular mechanisms of colorectal cancer liver metastasis (CRCLM) is crucial for developing effective preventive and therapeutic strategies.

Hepatic stellate cells (HSCs), liver-specific pericytes, are characterized by their storage of vitamin A and location in the space of Disse between the endothelial lining and hepatocytes [5]. Under normal conditions, HSCs maintain a quiescent and nonproliferative phenotype [6]. However, during the process of CRCLM, HSCs are activated by tumor-derived signals and become the primary source of cancer-associated fibroblasts (CAFs) [7]. These HSC-derived CAFs remodel the liver pre-metastatic microenvironment by depositing extracellular matrix (ECM) proteins, secreting cytokines and growth factors, and creating an immunosuppressive tumor microenvironment to accelerate tumor colonization [8, 9]. Previous studies have found that CRC cells-secreted fibroblast growth factor-19 (FGF19) binds to fibroblast growth factor receptor 4 on HSCs, promoting their transdifferentiation into CAFs and releasing angiopoietin-like 4, which in turn facilitates CRC cell migration to the liver [10]. Additionally, FGF19 polarizes HSCs into inflammatory CAFs that promote neutrophil infiltration and neutrophil extracellular trap formation within the liver metastatic niche, further facilitating the liver colonization of CRC cells [11]. Transforming growth factor β (TGF-β)-stimulated HSCs undergo metabolic reprogramming marked by upregulated glucose transporter 1, which enhances glucose uptake and glycolysis to drive HSC activation, thereby fostering the creation of a pro-metastatic niche conducive to CRCLM via the secretion of tumor-promoting factors [12]. Thus, targeting the activation of HSCs into CAFs represents a promising strategy to suppress the pro-metastatic liver microenvironment and prevent CRCLM.

As an abundant component of the hepatic tumor microenvironment, TGF-β is one of the most potent factors for CAF activation of HSCs [13]. TGF-β induces HSC activation via activating a TGF-β signaling cascade, including ligation of TGF-β receptor II (TβRII) and TGF-β receptor I (TβRI) at the plasma membrane (PM), phosphorylation of SMAD2/3, and induction of a SMAD2/3/4 complex in the cytoplasm, and translocation of the SMAD complex into the nucleus for gene transcription [14, 15]. Since TβRII is an initiator of the signaling cascade, its PM localization, internalization, trafficking, and degradation play a decisive role in HSC activation [16]. For instance, IQ motif-containing GTPase-activating protein 1 (IQGAP1) and vasodilator-stimulated phosphoprotein (VASP) influence HSC activation by controlling the recycling and degradation of TβRII of HSCs [17, 18].

Tuftelin 1 (TUFT1) is a highly conserved acidic protein, initially identified for its predominant expression in ameloblasts and closely associated with tooth enamel mineralization and structural organization [19, 20]. Subsequent studies have confirmed that TUFT1 expression extends beyond tooth enamel to various non-mineralizing tissues [21]. TUFT1 is now recognized to play crucial roles in the pathogenesis of diverse diseases, especially in malignant tumors [22-24]. Its oncogenic functions are mediated through multiple mechanisms. For instance, TUFT1 enhances the epithelial-mesenchymal transition (EMT) process to promote liver metastases of pancreatic cancer by regulating HIF1-Snail signaling [25]. In hepatocellular carcinoma, TUFT1 interacts with CREB1 to reprogram fatty acid metabolism, facilitating lipid accumulation [26]. Beyond these roles, TUFT1 activates several key oncogenic pathways, including PI3K-AKT, Rab5-Rac1, and Wnt signaling [27-29]. Additionally, TUFT1 is elevated in CRC cells, which drives the initiation and progression of CRCLM [29, 30]. Although TUFT1 has been studied in tumor cells, its function within the tumor stroma, particularly in activated-HSCs/CAFs, remains largely unexplored and warrants further investigation.

In this study, we show that primary human HSCs in culture and the CAFs of patient CRCLM express TUFT1. Knocking down TUFT1 inhibits TGF-β1/SMAD signaling and suppresses myofibroblastic activation of HSCs induced by TGF-β1. Mechanistically, the N-terminal amino acids (a.a.) 1–86 and a.a. 87–157 fragments of TUFT1 competitively disrupt caveolin-1 (CAV1) binding to TβRII so that TβRII is retrieved from the CAV1-mediated degradation pathway into an endosome-mediated protein trafficking and signaling pathway required for HSC activation. In a CRC/HSC coimplantation mouse model, targeting TUFT1 of HSCs by shRNA significantly suppresses CRC growth in mice; in an experimental liver metastasis model, targeting HSC TUFT1 by Cre/LoxP recombination inhibits CAF activation of HSCs and mitigates liver metastasis of CRC in mice. Together, our data reveal a novel role of TUFT1 and uncover a previously unrecognized mechanism underlying CAF activation of HSCs and the pro-metastatic liver microenvironment.

## MATERIALS AND METHODS

### Clinical samples and study approval

Human CRCLM and adjacent normal tissues were obtained from the First Affiliated Hospital of Xi’an Jiaotong University (Xi’an, China). The collection and use of specimens were approved by the Ethics Committee at the First Affiliated Hospital of Xi’an Jiaotong University (ethical approval number XJTU1AF2025LSYY-558). CAFs were isolated from the liver metastasis tissues in patients undergoing hepatic resection for CRCLM as previously described [31], and matched HSCs were isolated from the adjacent normal liver tissues as previously reported [32]. All participating patients were informed of the study and signed a written informed consent form.

### Mice and ethics statement

All animal operations were carried out with the approval of the Institutional Animal Care and Use Committee of Xi’an Jiaotong University.Five-week-old female BALB/c nude mice were purchased from the Laboratory Animal Center of Xi’an Jiaotong University. The nude mice were randomly grouped (*n* = 6 per group). TUFT1 conditional knockout mice in the C57BL/6J genetic background were generated by Cyagen Biosciences (Suzhou, China) Inc. using the CRISPR/Cas9-mediated genome engineering technique. The TUFT1 floxed mutant mouse line and the Col1a2-CreER transgenic mouse line were generated by Cyagen Biosciences (Suzhou, China). In the mouse model of CRCLM, there were 7 mice in each group. All mice were housed and bred in a specific pathogen-free environment at the Laboratory Animal Center of Xi’an Jiaotong University under a controlled temperature (25 °C) and 12-h day-night cycle.

### Reagents

Recombinant human TGF-β1 protein (#7754-BH) was obtained from R&D Systems (Minneapolis, USA). Cycloheximide (CHX) (#HY-12320), MG132 (#HY-13259), Aloxistatin (E64d) (#HY-100229), Pepstatin A (#HY-P0018), Bafilomycin A1 (#HY-100558), Methyl-β-cyclodextrin (MβCD) (#HY-101461), and Tamoxifen (#HY-13757) were purchased from MCE (Shanghai, China).

### Cell culture

Primary human HSCs were purchased from Yizefeng Bioscience Inc. (Shanghai, China). LX2 cell lines and MC38 mouse colon cancer cells were purchased from iCell Bioscience Inc. (Shanghai, China). HT29, HCT116, and 293T cell lines were obtained from the Cell Bank, Chinese Academy of Sciences (Shanghai, China). Primary human HSCs used for experiments were maintained at low passage numbers (< 7). HT29 cells were grown in McCoy’s 5 A medium (Procell, China) containing 10% fetal bovine serum (FBS; ExCell Bio, China) and 1% penicillin-streptomycin (Gibco, USA). Primary human HSCs and LX2 cells were cultured in Dulbecco’s Modified Eagle’s Medium (DMEM; CellMax, China) supplemented with 2% FBS and 1% penicillin-streptomycin. The isolated CAFs were cultured in Fibroblast Medium (ScienCell, USA). The isolated HSCs were cultured in Stellate Cell Medium (ScienCell, USA). Other cells were cultured in DMEM (CellMax, China) supplemented with 10% FBS and 1% penicillin-streptomycin. All cells were maintained at 37 °C with 5% CO_2_ in an incubator. Cells were routinely tested for mycoplasma contamination and were verified to be free of mycoplasma contamination.

### Plasmid construction, transfection, and lentiviral transduction of cells

All lentiviral short hairpin RNA (shRNA) plasmids, control shRNA plasmid, TUFT1-3×Flag, and TβRII-HA overexpression plasmids with the tag at the C-terminus and corresponding truncated plasmids were constructed by Tsingke Biotechnology (Nanjing, China). The detailed sequences of all shRNAs were listed in Table S1. Transient transfection was performed using the polyethylenimine (PEI; #40816ES03, Yeasen, China) reagent according to the manufacturer’s instructions. In the context of lentiviral packaging, the target plasmids were co-transfected with the psPAX2 packaging plasmid and pMD2.G envelope plasmid into 293T cells using PEI transfection reagent. At 48 h and 72 h following transfection, viral supernatants were harvested and filtered through a 0.45 μm filter. Then, primary human HSCs and LX2 cells were infected with lentiviruses (fresh medium: filtered viral supernatants=1:1) in the presence of 8 μg/mL polybrene (#H8761, Solaibao, China). 72 h after lentiviral transduction, cells were collected for subsequent experiments.

### RNA isolation and quantitative real-time PCR (qRT-PCR)

The total RNA was isolated from the cells using Trizol (#15596018CN, Invitrogen, USA). Reverse transcription was carried out using the ABScript III RT Master Mix for qPCR kit (#RK20428, ABclonal, China). qRT-PCR was performed with 2X Universal SYBR Green Fast qPCR Mix (#RK21203, ABclonal, China) on a CFX96 Touch real-time PCR detection system (Bio-Rad, USA). GAPDH was included as a reference gene, and the relative gene expression was calculated using the 2^−ΔΔCt^ method. The primers were shown in Table S2.

### Western blot (WB)

Cells or tumor tissues were lysed with RIPA lysis buffer (#P0013B, Beyotime, China) supplemented with PMSF (#ST507, Beyotime, China), protease inhibitors (#P1005, Beyotime, China), and phosphatase inhibitors (#P1081, Beyotime, China). After protein quantification (BCA assay kit; #P0010S, Beyotime, China), 20 μg of denatured total protein was electrophoresed on SDS-PAGE gel and transferred to PVDF membranes (#1620177, Bio-Rad, USA) by wet-transfer system. The membranes were blocked in 5% non-fat milk at room temperature for 1 h, then incubated with primary antibodies overnight at 4 °C. Following this, the membranes were washed three times with TBST for 10 min and incubated at room temperature with secondary antibodies conjugated to horseradish peroxidase for 1 h. The protein bands were detected using Amersham Imager 680 (GE Healthcare, USA). The relative quantification of protein bands was determined using the ImageJ software (NIH). Details of antibodies and dilutions were provided in Table S3.

### Protein half-life assay

To explore the effect of TUFT1 on TβRII protein stability, primary human HSCs or LX2 cells were transduced with lentiviruses expressing control shRNA or TUFT1 shRNA. After 72 h of virus transduction, cells were treated with 40 μg/mL CHX to block protein synthesis and harvested at the indicated time points. Cell lysates were analyzed by WB to measure the protein level of TβRII at different times.

### Co-immunoprecipitation (Co-IP)

Cells were lysed with the IP lysis buffer (#P0013, Beyotime, China) containing PMSF (#ST507, Beyotime, China), protease inhibitors (#P1005, Beyotime, China), and phosphatase inhibitors (#P1081, Beyotime, China). A fraction of the supernatant was used as input for the total protein lysate. Then, the remaining protein supernatant was incubated overnight with the primary antibody and protein A/G plus-agarose beads (#sc-2003, Santa Cruz, USA) on a rotator at 4 °C. Next, the agarose beads were washed five times with the prechilled NETN lysis buffer (100 mM NaCl, 20 mM Tris-Cl pH 8.0, 0.5 mM EDTA, 0.5% Nonidet P-40), and the precipitated proteins were eluted from the agarose beads by adding 2×SDS-PAGE loading buffer and denaturing at 95 °C for 5 min. After centrifugation, the immunoprecipitates were subjected to WB. Antibodies used were listed in Table S3.

### Immunohistochemistry (IHC) and immunofluorescence (IF) staining

Tissue samples were fixed in 4% paraformaldehyde, embedded in paraffin, and cut into sections of 5 μm. According to the manufacturer’s instructions, IHC was performed using the PV-6001 or PV-6002 two-step kits and the DAB chromogenic kit (#ZLI-9018, ZSGB-Bio, China). The IHC stain scoring was carried out as described previously [33]. For IF staining, paraffin sections were deparaffinized and hydrated for antigen repair. The cells were fixed using 4% paraformaldehyde for 20 min and then permeabilized with 0.3% Triton X-100 for 5 min at room temperature. Tissue sections or cells were incubated with primary antibodies overnight at 4 °C after blocking in 5% bovine serum albumin for 1 h. The following day, after washing with PBS, the tissue sections or cells were incubated with fluorescence-labeled secondary antibodies for 1 h at room temperature, and the nucleus was counterstained with DAPI (#40728ES03, Yeasen, China) for 10 min. Fluorescence images were visualized and acquired with either a Zeiss Axio Observer 7 fluorescence microscope (Zeiss, Germany) or an Olympus FV3000 confocal microscope (Olympus, Japan). Analysis of intensity and co-localization was performed using ImageJ (NIH). Antibodies used were listed in Table S3.

### Polysome profiling

Polysome profiling was conducted according to previously described [34]. Briefly, cells were incubated with 100 μg/ml CHX for 5 min, digested with trypsin, collected the cell precipitate by centrifugation, and washed twice with cold PBS. Precipitated cells were lysed in lysis buffer (100 mM KCl, 5 mM MgCl_2_, 20mM HEPES-KOH pH 7.4, 1× EDTA-free protease inhibitor cocktail, 100 μg/ml CHX, 20 U/mL RNase inhibitor, 1% Triton X-100, and 2 mM DTT). After centrifuging at 16000 × g for 10 min at 4 °C, the supernatants were loaded on 10–45% sucrose gradients and centrifuged at 36000 rpm for 3 h in a SW41 Ti rotor (Beckman Optima XE-90, Germany). Then, samples were monitored and fractionated with a gradient fractionator (Biocomp, Canada). After fraction collection, RNA was isolated from each fraction using the TRIzol (Invitrogen, USA) method. qRT-PCR was performed to measure mRNA expression in each fraction.

### Biotin-labeled pull-down assay

To qualify the expression of TβRII on the cell membrane, primary human HSCs or LX2 cells after infection with the lentivirus were starved for 24 h in a serum-free medium and then stimulated with TGF-β1 (5 ng/mL) for 6 h. Or cells were infected with lentivirus and then directly used for subsequent experiments. Next, cells were incubated with 1 mg/ml EZ-Link Sulfo-NHS-Biotin (#21217, Thermo Scientific, USA) for 30 min at 4 °C. Cells were washed 3 times with cold PBS to remove excess unbound biotin, and the lysis buffer was added to the dishes to lyse the cells. After centrifugation, supernatants were incubated with streptavidin agarose beads (#S1638, Millipore, USA) overnight at 4 °C on a rotating wheel. After 5 washes, streptavidin agarose beads were denatured with 2×SDS-PAGE loading buffer and subjected to WB analysis. Streptavidin-HRP (#434323, Thermo Scientific, USA) was applied to detect total biotinylated proteins.

### Luciferase reporter assay

The TUFT1 luciferase reporter plasmid was constructed by Professor Lianying Jiao’s laboratory (Xi’an Jiaotong University). 293T cells were transfected using PEI transfection reagent according to the manufacturer’s instructions. 24 h after transfection, cells were incubated with or without TGF-β1 (5 ng/mL) for 48 h. Then, firefly luciferase activity was detected with the Firefly Luciferase Reporter Gene Assay Kit (#RG005, Beyotime, China) and was normalized using a β-galactosidase reporter assay (#RG0036, Beyotime, China).

### Chromatin immunoprecipitation (ChIP) and ChIP-qPCR

The primary human HSCs were treated with TGF-β1 (5 ng/mL), and then the cells were harvested for ChIP experiments. The ChIP assay was performed as previously reported [35]. In brief, chromatin was cross-linked with 1% formaldehyde, and cells were lysed and sonicated. Chromatin fragments were immunoprecipitated with ChIP-grade SMAD2/3 antibody (#8685, CST, USA) or control IgG antibody (#A7058, Beyotime, China) overnight at 4 °C and then added with 50 μL BeyoMag^™^ Protein A + G magnetic beads (#P2108, Beyotime, China) for 2 h 4 °C. After IP, the protein-DNA cross-links were reversed, and the DNA was purified, followed by qPCR. The primers for ChIP-qPCR were presented in Table S4.

### RNA sequencing and bioinformatic analyses

Primary human HSCs transduced with control shRNA or TUFT1 shRNA lentiviruses were starved with serum-free medium for 24 h and then stimulated with TGF-β1 (5 ng/ml) for 24 h. RNA from primary human HSCs was isolated using the Trizol (#15596018CN, Invitrogen, USA) method. RNA quality control, library construction, sequencing, and data processing were performed by Shanghai Personal Biotechnology Co. Ltd. All raw RNA sequencing data were uploaded to the NCBI SRA database (accession No: PRJNA1165333). In brief, library preparation was completed using the NEBNext Ultra Directional RNA Library Prep Kit for Illumina (#E7420S, NEB, USA) according to the supplier’s recommendations. The cDNA was purified with AMPure XP beads (#A63880, Beckman Coulter, USA) to obtain 400 to 500 bp cDNA fragments, amplified by PCR, and the PCR products were purified again using AMPure XP beads. The quality of the cDNA library was detected using the Agilent high-sensitivity DNA kit (#5067-4626, Agilent Technologies, USA) on the Bioanalyzer 2100 system (Agilent Technologies, USA). The library was then sequenced on the NovaSeq 6000 platform (Illumina, USA). Clean reads were mapped to the GRCh38/hg38 reference genome using HISAT2 v2.0.5. Differentially expressed genes were identified using the R package DESeq2 (∣log2 fold change∣ >1, *p* < 0.05). GO enrichment analyses were conducted using the R package clusterProfiler. Gene set enrichment analysis (GSEA) was applied with the enrichplot R package.

### Spatial transcriptomic analysis

The public spatial transcriptomic data for the ST-P1 sample of CRCLM were downloaded from the web portal (http://www.cancerdiversity.asia/scCRLM/) [36]. Using this data, the expression of TUFT1 was analyzed across different cell types in CRCLM tissues. In brief, deconvolution analysis was performed with the “SPOTlight” package to determine the proportion of each cell type at each spot. We performed quality control of single-cell transcriptome data based on expressed gene number, unique molecular identifier counts, and mitochondrial RNA percentages in each cell. Subsequently, the average expression of the top 25 specific genes was calculated for each cell type to construct a signature score matrix. The enrichment scoring matrix was generated using the get enrichment matrix and enrichment analysis functions in the Cottrazm package. Gene spatial expression was visualized by the “SpatialFeaturePlot” in the Seurat package.

### Single-cell RNA sequencing (scRNA-seq) data access and processing

scRNA-seq data GSE160541 were downloaded from the Gene Expression Omnibus (GEO, https://www.ncbi.nlm.nih.gov/geo) database [37]. We chose the murine CRCLM sample GSM4874984 for the following analysis. The raw gene expression matrix was converted to a Seurat object using the “Seurat” R package. Cells with fewer than 300 detected genes and genes expressed in less than 5 cells were discarded for infiltration and quality control. Following normalizing and scaling the data, principal component analysis and Uniform Manifold Approximation and Projection (UMAP) were performed for dimension reduction. After that, we conducted the cell clustering and annotation using the “SingleR” R package. Finally, we separated 15 clusters and identified 6 cell types. The expression of genes in different cells was visualized using the “FeaturePlot” function in UMAP graphics.

### Immunoprecipitation-mass spectrometry (IP-MS)

Primary human HSCs were transduced with TβRII-HA lentiviruses. After 72 h, HSCs were lysed with IP lysis buffer, and then the supernatant was harvested for co-IP assay as described above. After electrophoresis, immunoprecipitated proteins were stained with Coomassie brilliant blue (#P0017F, Beyotime, China). Subsequent enzymatic hydrolysis and liquid chromatography coupled tandem mass spectrometry (LC-MS) analysis were performed at the Jingjie PTM BioLab Co., Ltd. (Hangzhou, China). The raw data of LC-MS were processed using Proteome Discoverer 2.4.

### Cell proliferation assay

Conditioned medium (CM) was collected from primary human control HSCs and HSCs with TUFT1 knockdown. After treatment with CM, HT29 or HCT116 cells were seeded in 96-well plates at 5 × 10^3^ cells per well. Next, cell viability was detected at different time points (24 h, 48 h, and 72 h) using the CCK8 kit (#E-CK-A362, Elabscience, China), and the absorbance at 450 nm was measured by a microplate reader (Thermo Scientific, USA). For the colony formation assay, HT29 or HCT116 cells were plated in 6-well plates at a density of 800 cells per well and then subjected to stimulation with CM for 48 h after an overnight culture. Later, the culture medium was replaced with fresh medium for 12 days of culture. Cell clones were fixed with 4% paraformaldehyde, stained with crystal violet solution, and photographed. For the EdU assay, after being treated with CM, HT29 or HCT116 cells were incubated with 50 μM EdU for 2 h and then underwent EdU staining according to the kit instructions (#C10310-3, Ruibo, China). Fluorescence images were acquired by a Zeiss Axio Observer 7 fluorescence microscope.

### Transwell migration and invasion assays

For the migration assay, HT29 or HCT116 cells (3 × 10^4^ cells in 200 μl) were seeded in the upper transwell chambers (#3422, Corning, USA), and the CM was added to the lower chambers. After 48 h of co-culture, the chambers were fixed with 4% paraformaldehyde, stained with crystal violet solution, and photographed under a microscope. For the invasion assay, the membranes of transwell chambers were pre-coated with Matrigel (#354234, BD, USA), and the remaining steps were the same as described above.

### Co-injection of HT29 and HSCs into mice

HSCs were transduced with control shRNA or TUFT1 shRNA lentiviruses. 72 h after viral transduction, 1 × 10^6^ primary human HSCs mixed with 1 × 10^6^ human HT29 colon cancer cells were re-suspended in 100 μL of PBS and injected subcutaneously into the flank of BALB/c nude mice. Tumor sizes were measured every 3 days via a vernier caliper, and tumor volume was calculated using a formula: tumor volume = 0.5 × length × width^2^. The mice were sacrificed 21 days after tumor injection, and tumors were isolated for subsequent experiments.

### Mouse genotyping

Genomic DNA was extracted from mouse tail tips using the MiniBEST Universal Genomic DNA Extraction kit (#9765, TaKaRa, China). Then, 1.5 μl of extracted DNA was used for PCR validation. The primers used in PCR were as follows: LoxP sites Forward: 5’-CTGTTCGTTCCAGTGTGGTTTCTA-3’ and LoxP sites Reverse: 5’-AGAGACACACTCCTATCTTTCCAC-3’; Col1a2-CreER-Forward: 5’-CAGGAGGTTTCGACTAAGTTGG-3’ and Col1a2-CreER-Reverse: 5’-CATGTCCATCAGGTTCTTGC-3’.

### Mouse model of CRCLM

To generate CRCLM, 10-week-old TUFT1^flox/flox^ Col1a2-CreER mice and matched control TUFT1^flox/flox^ mice were subjected to intraperitoneal injection of tamoxifen (50 mg/kg/day) for 7 consecutive days to activate the Cre recombinase of the mice. Tumor injection was performed 5 days after the tamoxifen injection cycle, which was done by injecting 5 × 10^5^ MC38 murine CRC cells in 50 μL PBS into the portal vein of the mouse [38].

### Statistical analysis

Data are presented as the mean ± standard deviation (SD). Differences between the two groups were analyzed by unpaired Student’s t-tests. One-way or two-way ANOVA was applied to analyze the differences among more than two groups, which was completed with the GraphPad Prism 9.0 software. Statistical significance was defined as a p-value less than 0.05.

### Ethics approval

The collection and use of human specimens were approved by the Ethics Committee at the First Affiliated Hospital of Xi’an Jiaotong University (ethical approval number XJTU1AF2025LSYY-558). All animal operations were carried out with the approval of the Institutional Animal Care and Use Committee of Xi’an Jiaotong University.

## RESULTS

### Mass spectrometry identified TUFT1 as a TβRII-binding protein in primary human HSCs

**Fig. 1:**
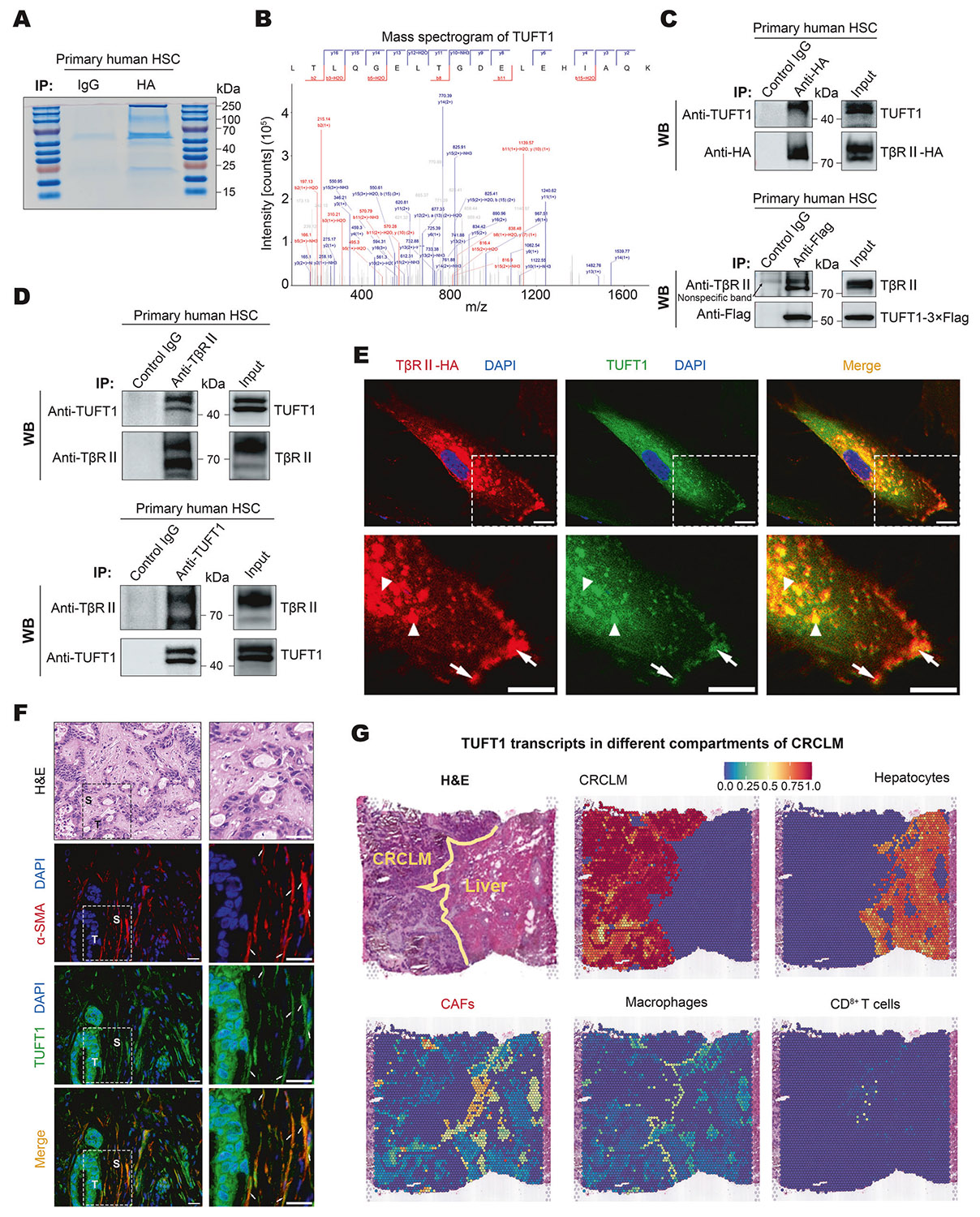
TUFT1 binds to TGF-β receptor II (TβRII) in HSCs. **A** Immunoprecipitation (IP) was performed with primary human HSCs overexpressing TβRII-HA fusion protein with anti-HA antibody. The co-precipitates were subjected to SDS-PAGE and Coomassie blue staining. **B** Mass spectrometry analysis identified TUFT1 as a TβRII-binding protein. **C** Primary human HSCs with overexpression of TβRII-HA (upper) or TUFT1-3×Flag (lower) were collected for co-IP with anti-HA antibody or anti-Flag antibody; co-precipitated TUFT1 or TβRII was detected by Western blot (WB) analysis. Data represent 3 repeats with similar results. **D** The interaction between endogenous TβRII and TUFT1 in primary human HSCs was verified by co-IP with anti-TβRII antibody (upper) or anti-TUFT1 antibody (lower). Data represent 3 repeats with similar results. **E** Double immunofluorescence staining (IF) showing co-localization of TβRII-HA (red) and TUFT1 (green) at the plasma membrane (arrows) and in the endosomes (arrowheads) of primary human HSCs. Nuclei were stained with DAPI (blue). Scale bar, 10 μm. **F** Sections of a patient’s colorectal cancer liver metastasis (CRCLM) sample were subjected to H&E staining and double IF staining for α-SMA (red) and TUFT1 (green). Nuclei were stained with DAPI (blue). α-SMA and TUFT1 co-localization was detected in the stroma of CRCLM (arrows). Scale bar, 50 μm. **G** Spatial transcriptomics of a patient CRCLM (ST-P1) revealed that TUFT1 transcripts were detected from CRC cells, the hepatocytes, cancer-associated fibroblasts (CAFs), macrophages, and CD8^+^ T cells. The minimum level (blue) to the maximum level (red) is shown by a color bar.

To identify proteins that interacted with TβRII, we generated TβRII-HA-overexpressing HSCs by transducing primary human HSCs with lentiviruses encoding TβRII-HA. IP performed with anti-HA antibody, followed by mass spectrometry (MS) for TβRII-HA binding proteins, revealed that TUFT1 bound to TβRII-HA in HSCs (Fig. 1A, B). Co-IP with anti-HA confirmed that endogenous TUFT1 co-immunoprecipitated with overexpressed TβRII-HA in primary human HSCs (Fig. 1C upper) and LX2 cells (Fig. S1A Left). Conversely, TUFT1–3×Flag-overexpressing cells were subjected to co-IP with anti-Flag antibody, revealing that endogenous TβRII co-immunoprecipitated with overexpressed TUFT1–3×Flag in primary human HSCs (Fig. 1C lower) and in LX2 cells (Fig. S1A right). Moreover, the binding between endogenous TβRII and endogenous TUFT1 was validated by co-IP as well, using primary human HSCs (Fig. 1D) and LX2 cells (Fig. S1B). The presence of multiple bands for TβRII in our co-IP results was a consistent finding, which might be due to the post-translational modifications prevalent in this protein [39]. Double IF for TβRII-HA and TUFT1 demonstrated co-localization of the two proteins at the PM (arrows, Fig. 1E) and in the endosomes (arrowheads, Fig. 1E) of HSCs. Given that TUFT1 can influence vesicular trafficking of mTORC1 via physical interaction with RABGAP1 [40], we hypothesized that TUFT1 may bind to TβRII to regulate TβRII trafficking. The hypothesis was tested using the experiments shown below.

### TUFT1 protein and transcripts were detected in activated-HSCs/CAFs of patient CRCLM

As revealed by H&E staining, CRC cells are embedded in rich stromal tissues in a patient CRCLM (Fig. 1F). Double IF for α-smooth muscle actin (α-SMA) (a marker of activated-HSCs/CAFs) (red, Fig. 1F) and TUFT1 (green, Fig. 1F) revealed co-localization of α-SMA IF signals and TUFT1 IF signals in a patient CRCLM (yellow, Fig. 1F), indicating that TUFT1 protein was expressed in the CAFs of patient CRCLM. TUFT1 IF signals were also detected from CRC cells, consistent with the data of a prior study [29, 30]. Immunohistochemistry confirmed TUFT1 protein expression in the liver metastatic foci and stromal tissues (Fig. S2A). Additionally, a positive correlation between TUFT1 and TβRII protein level was detected in the stromal compartment of CRCLM tissues (Spearman *R* = 0.776, *P* < 0.001, Fig. S2B). Next, we used bioinformatic approaches to analyze publicly available spatial transcriptomic data of the ST-P1 sample of a patient CRCLM [36] for spatial localization of TUFT1 transcripts (Fig. 1G). The highest TUFT1 transcript levels were detected from CRC cancer cells, followed by the hepatocytes (top row in Fig. 1G). Notably, among the CRCLM stromal cells, the highest TUFT1 transcript levels were detected from the CAFs, followed by the macrophages, and very low levels of TUFT1 transcripts were detected from CD8^+^ T cells (bottom row in Fig. 1G). Thus, activated-HSCs/CAFs of patient CRCLM indeed express TUFT1 transcripts and TUFT1 protein.

### The protein level of TβRII, but not that of TβRI, is reduced by TUFT1 targeting shRNA

**Fig. 2:**
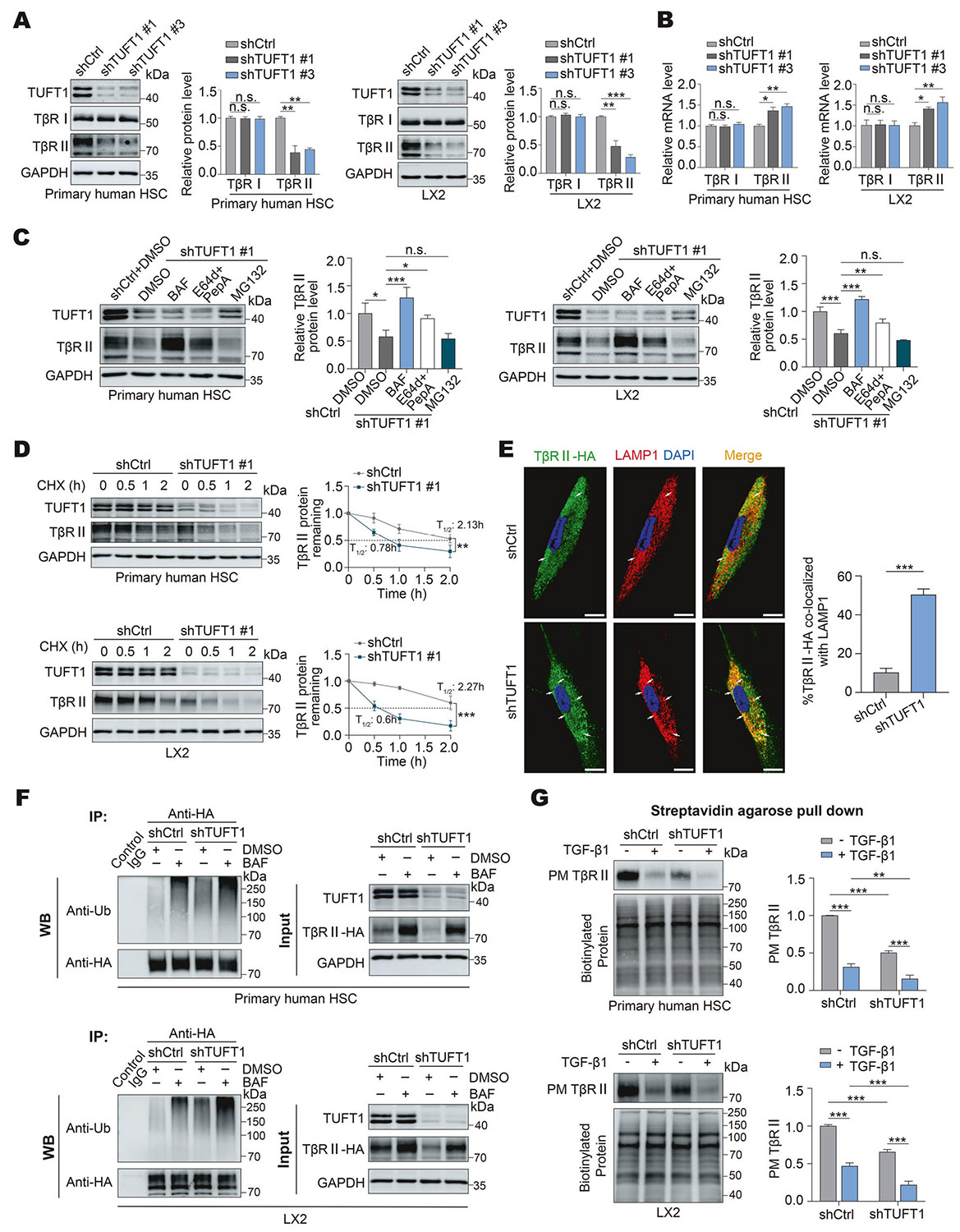
Targeting TUFT1 by shRNA promotes ubiquitination and lysosome-mediated degradation of TβRII in HSCs. **A** WB revealed that knocking down TUFT1 by one of two different shRNA lentiviruses reduced the protein level of TβRII, but not TβRI, in primary human HSCs (left) and LX2 cells (right). Not significant (n.s., *P*> 0.05), **P < 0.01, ***P < 0.001 by ANOVA, n = 3. **B** qRT-PCR revealed that knocking down TUFT1 induced upregulation of the mRNA level of TβRII, but not TβRI in primary human HSCs (left) and LX2 cells (right). Not significant (n.s., *P* > 0.05), *P < 0.05, **P < 0.01 by ANOVA, *n* = 3. **C** Primary human HSCs (left) or LX2 cells (right) expressing TUFT1 shRNA #1 were incubated with lysosomal inhibitors (bafilomycin A1 [BAF, 10 nM] or E64d [10 μg/mL] + pepstatin A [PepA, 10 μg/mL]) or a proteasomal inhibitor MG132 (25 μM) for 8 h followed by WB for TβRII. The TβRII protein levels were restored by the lysosomal inhibitors, but not by the proteasomal inhibitor. Not significant (n.s., *P* > 0.05), *P < 0.05, **P < 0.01, ***P < 0.001 by ANOVA, *n* = 3. **D** Primary human HSCs (upper) or LX2 cells (lower) expressing TUFT1 shRNA #1 were incubated with cycloheximide (CHX) (40 μg/mL) and collected at the indicated times for WB for TβRII. TβRII was degraded much faster in TUFT1 knockdown cells compared to control cells. **P < 0.01, ***P< 0.001 by ANOVA, *n* = 3. **E** Double IF for TβRII-HA (green) and LAMP1 (red, a marker of lysosomes) was performed with control and TUFT1 knockdown HSCs. Representative confocal images revealed that the co-localization of the two proteins (yellow, arrows) was higher in TUFT1 knockdown HSCs compared to control cells. Scale bar, 15 μm. ***P < 0.001 by t-test, *n* = 12 cells per group. **F** Primary human HSCs (upper) or LX2 cells (lower) with overexpression of TβRII-HA were subjected to lentiviral transduction to create control and TUFT1 knockdown cells for co-IP. TβRII-HA was pulled down by anti-HA, and its ubiquitination level was quantified by WB with anti-ubiquitin. TβRII-HA ubiquitination level was higher in TUFT1 knockdown cells compared to control cells, which was further potentiated by incubation with 10 nM Bafilomycin A1 (BAF). Data represent 3 independent repeats with similar results. **G** Control and TUFT1 knockdown cells were stimulated with or without TGF-β1 (5 ng/mL) for 6 h, followed by biotinylation and streptavidin agarose pull-down to detect TβRII at the cell plasma membrane (PM TβRII). PM TβRII level was lower in TUFT1 knockdown cells compared to control cells, which was further reduced by TGF-β1 stimulation. **P < 0.01, ***P < 0.001 by ANOVA, *n* = 3.

We next used a shRNA-mediated knockdown approach to study the role of TUFT1 in TβRII and the TGF-β signaling of HSCs. To this end, we generated 4 different lentiviruses, each encoding a distinct TUFT1 shRNA, and a lentivirus encoding a non-targeting shRNA (shCtrl) was used as a control. Among the 4 TUFT1 shRNAs tested, shTUFT1 #1 and shTUFT1 #3 exhibited the strongest silencing effects, so they were used in subsequent experiments (*P* < 0.01, Fig. S3A, B). WB revealed that silencing TUFT1 by two different shRNAs significantly reduced the protein level of TβRII in both primary human HSCs and LX2 cells, whereas it did not alter the protein level of TβRII (*P*< 0.01, Fig. 2A). qRT-PCR for the transcripts of TβRI and TβRII, however, revealed that silencing TUFT1 elevated TβRII mRNA level in both primary human HSCs and LX2 cells but had no effect on TβRI mRNA level (*P*< 0.05, Fig. 2B). These data indicate that targeting TUFT1 only affected TβRII mRNA and protein, and that it may downregulate TβRII via a post-transcriptional mechanism.

### Targeting TUFT1 leads to lysosomal degradation of TβRII

To determine whether TUFT1 regulates the biology of TβRII by regulating TβRII translation or protein degradation, we performed polysome profiling by sucrose gradient centrifugation, followed by qRT-PCR, to quantify the level of TβRII mRNA in polysome fractions. The polysome distribution profiles did not change in the control and TUFT1 knockdown groups (Fig. S4A), supporting that knocking down TUFT1 failed to influence the protein translation of the cell globally. qRT-PCR revealed that TβRII mRNA level in 40S, 60S, 80S, and polysome fractions remained unchanged in TUFT1 knockdown cells compared to control cells (*P*> 0.05, Fig. S4B), indicating that TβRII translation was not altered by TUFT1 knockdown.

We have previously reported that TβRII is degraded by late endosomes/lysosomes of HSCs [41, 42]. To test whether targeting TUFT1 led to lysosomal degradation of TβRII, TUFT1 knockdown primary human HSCs and LX2 cells were incubated with either lysosomal inhibitors (bafilomycin A1 [10 nM] or E64d [10 μg/mL] + pepstatin A [10 μg/mL]) or a proteasome inhibitor (MG132, 25 μM) for 8 h, and WB was performed for TβRII of the cells. WB revealed that the lysosomal inhibitors, as opposed to the proteasomal inhibitor, restored the TβRII protein levels in both primary human HSCs and LX2 cells expressing shTUFT1 #1 (*P* < 0.05, Fig. 2C). Consistent data were obtained from the experiments using shTUFT1 #3 to knock down TUFT1 (*P* < 0.01, Fig. S4C, D). Thus, targeting TUFT1 by shRNA leads to lysosomal degradation of TβRII.

We next performed experiments and studied the influence of TUFT1 on the protein stability of TβRII. To this end, control and TUFT1 knockdown cells were incubated with 40 μg/mL cycloheximide (CHX) to inhibit protein synthesis of HSCs, and WB was performed for cells at different time points to chase TβRII protein level, which demonstrated that TβRII was downregulated much faster (with a shorter half-life) in both primary human HSCs and LX2 cells expressing shTUFT1 #1 compared to their respective control cells (*P* < 0.01, Fig. 2D). Consistent data were obtained from the experiments using shTUFT1 #3 to knock down TUFT1 (*P* < 0.001, Fig. S4E, F). Double IF for TβRII-HA and lysosomal-associated membrane protein 1 (LAMP1) (a marker of late endosomes/lysosomes) and confocal microscopy showed that the rate of TβRII-HA/LAMP1 co-localization was enhanced in TUFT1 knockdown HSCs compared to control HSCs (*P* < 0.001, Fig. 2E). Thus, silencing TUFT1 indeed leads to lysosomal targeting and degradation of TβRII in HSCs.

### Targeting TUFT1 enhances ubiquitination of TβRII

Ubiquitination is a requisite for lysosomal targeting and degradation of TβRII [17, 42]. We therefore determined the influence of TUFT1 knockdown on the ubiquitination of TβRII in HSCs. To this end, primary human HSCs and LX2 cells overexpressing TβRII-HA were subjected to IP with an anti-HA antibody, and ubiquitination on TβRII-HA precipitated was measured by WB. As revealed by WB, the basal levels of TβRII ubiquitination in control primary human HSCs and LX2 cells were low, which were elevated by the lysosomal inhibitor bafilomycin A1 (Fig. 2F). Importantly, both levels were potentiated by TUFT1 knockdown in both primary human HSCs and LX2 cells (Fig. 2F), supporting that TUFT1 knockdown indeed promoted TβRII ubiquitination that led to its lysosomal targeting and degradation.

To investigate whether TUFT1 influences PM localization of TβRII in HSCs, cell surface TβRII was next quantified by a biotinylation assay, which demonstrated that PM TβRII was significantly reduced in TUFT1 knockdown primary human HSCs and LX2 compared to their controls (*P* < 0.01, Fig. 2G). Moreover, incubation of the primary human HSCs or LX2 cells with TGF-β1 for 6 h resulted in the downregulation of PM TβRII (*P*< 0.01, Fig. 2G), confirming that the PM TβRII level was negatively regulated by TGF-β1-mediated internalization and endocytosis of TβRII.

### Amino acids 1–157 of TUFT1 binds to and protects TβRII from lysosomal degradation

**Fig. 3:**
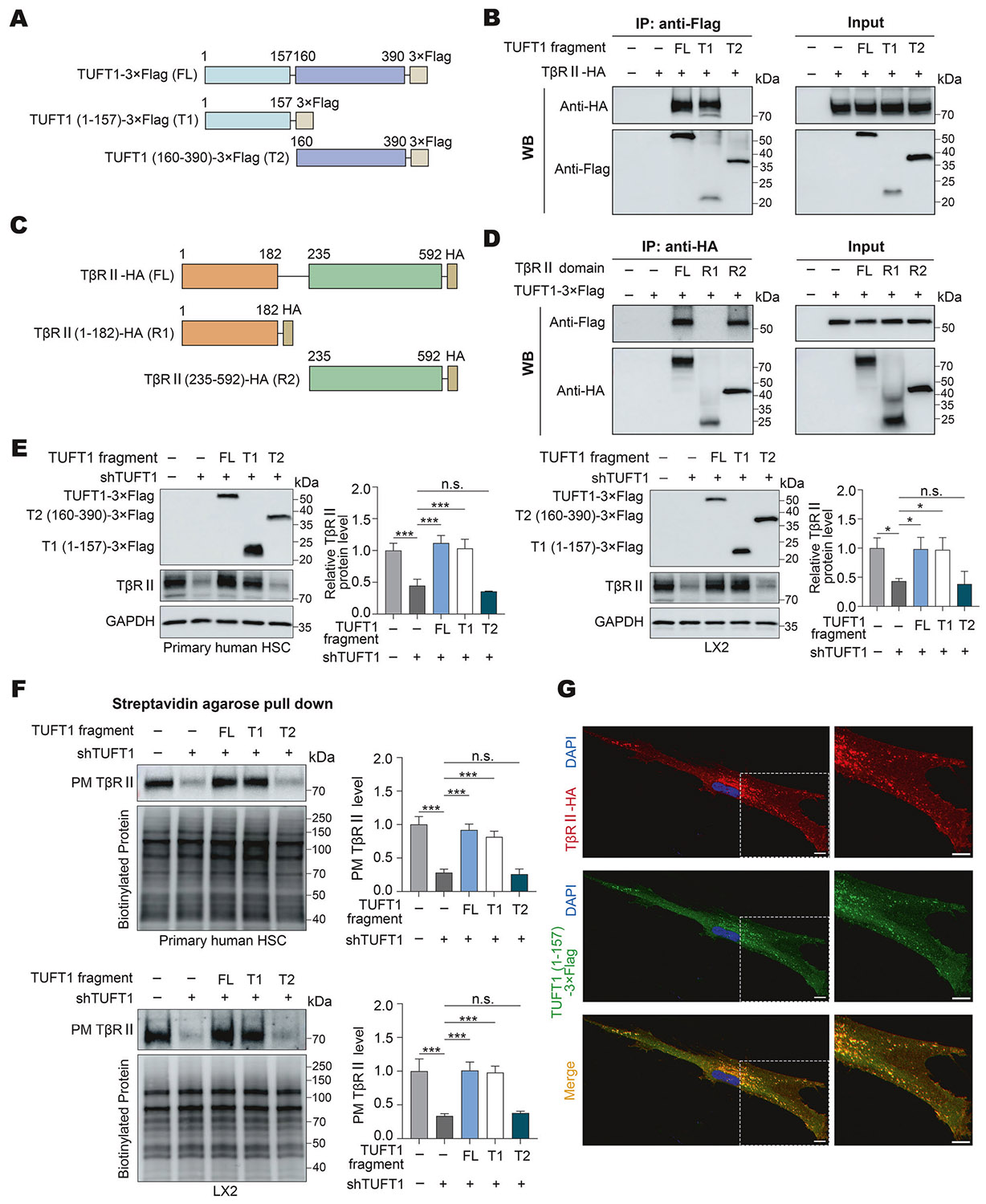
Amino acids 1-157 of TUFT1 binds to TβRII intracellular domain to stabilize TβRII protein. **A** Schematic representation of 3xFlag-tagged full-length (FL) and 2 truncation mutants of TUFT1. **B** 293T cells co-expressing TβRII-HA and one of the TUFT1 mutants were subjected to co-IP with anti-Flag; WB was performed to detect co-precipitated TβRII-HA. Amino acids 1-157 of TUFT1 binds to TβRII-HA in the cells. Data represent 3 independent repeats with similar results. **C** Schematic representation of TβRII truncation mutants. **D** 293T cells co-expressing 3×Flag-tagged FL TUFT1 and one of the TβRII mutants were subjected to co-IP with anti-HA; WB was performed to detect co-precipitated TUFT1. The intracellular domain of TβRII binds to TUFT1. Data represent 3 independent repeats with similar results. **E** Primary human HSCs (left) or LX2 cells (right) with co-expression of TUFT1 shRNA and one of the TUFT1 mutants were subjected to WB for TβRII. The TβRII protein levels affected by TUFT1 knockdown were restored by the FL TUFT1 and T1 mutant (a.a.1-157 of TUFT1), but not by the T2 mutant (a.a.160–390). Not significant (n.s., *P* > 0.05), *P < 0.05, ***P < 0.001 by ANOVA, *n* = 3. **F** Cells prepared as described above were subjected to a biotinylation assay to quantify PM TβRII protein level. The PM TβRII levels affected by TUFT1 knockdown were restored by the FL TUFT1 or the T1 mutant, but not the T2 mutant. Not significant (n.s., *P* > 0.05), ***P < 0.001 by ANOVA, *n* = 3. **G** Primary human HSCs co-expressing TβRII-HA and the T1 mutant were subjected to double IF for HA (red) and Flag (green). Co-localization of the two proteins was detected by fluorescence confocal microscopy (yellow). Scale bar, 10 μm.

To study how TUFT1 and TβRII interact in HSCs, we created their truncation mutants based on the lentiviral vector encoding TUFT1– 3×Flag or TβRII-HA. Because TUFT1 does not have a well-defined domain, we truncated TUFT1 from the middle based on its amino acid sequence. 2 truncation mutant constructs of TUFT1 were created with T1 encoding a.a. 1–157 and T2 encoding a.a. 160–390 of TUFT1 (Fig. 3A). 293T cells co-expressing a TUFT1 mutant and TβRII-HA were subjected to co-IP with anti-Flag, and coprecipitated TβRII-HA was detected by WB (Fig. 3B), which supported that the full-length (FL) TUFT1 or the T1 mutant, but not the T2 mutant, bound to TβRII-HA (Fig. 3B). To gain additional insight into TUFT1/TβRII interaction, two additional Flag-tagged TUFT1 truncation mutants, a.a. 1–86 of TUFT1 and a.a. 87–157 of TUFT1, were made for co-IP (Fig. S5A). Interestingly, co-IP demonstrated that both a.a. 1–86 and a.a. 87–157 fragments of TUFT1 interacted with TβRII in 293T cells (Fig. S5B). Additionally, we used the Boltz-2 [43] to predict the structure model of TUFT1 (a.a. 1–157)/TβRII complex, and structures were visualized using PyMOL (https://pymol.org/). As shown in Fig. S5C, TUFT1 (a.a. 1–86) and TUFT1(a.a. 87–157) interact with TβRII and form a semi-ring to wrap TβRII extensively. This computational prediction was consistent with our experimental result. Thus, TUFT1/TβRII interaction in HSCs is mediated by the a.a. 1–86 and a.a. 87–157 fragments of TUFT1.

We also created two truncation mutant constructs for TβRII-HA, with one encoding a.a. 1–182 of TβRII (R1) (extracellular domain of TβRII) and another encoding a.a. 235–592 (R2) (intracellular domain of TβRII) (Fig. 3C). Co-IP with anti-HA followed by WB demonstrated that the intracellular domain of TβRII (R2), but not the extracellular domain of TβRII-HA (R1), bound to TUFT1 (Fig. 3D).

Since the T1 mutant (a.a. 1–157) of TUFT1 binds to TβRII, we tested whether it was able to restore the TβRII protein levels of primary human HSCs and LX2 cells that were affected by TUFT1 knockdown. Overexpression of the FL TUFT1 was used as a control. Our data indeed revealed that, similar to the FL TUFT1, the T1 mutant, but not the T2 mutant, restored the TβRII protein levels (P < 0.05, Fig. 3E), as well as the PM TβRII protein levels (P < 0.001, Fig. 3F) affected by TUFT1 knockdown in both primary human HSCs and LX2 cells. Moreover, co-localization of TβRII-HA and the T1 mutant in the cytoplasm was confirmed by double IF (Fig. 3G). Thus, the N-terminal fragment of TUFT1 binds to TβRII and protects TβRII from lysosomal degradation.

### TβRII downregulation induced by TUFT1 targeting is mediated by lipid rafts/caveolae

**Fig. 4:**
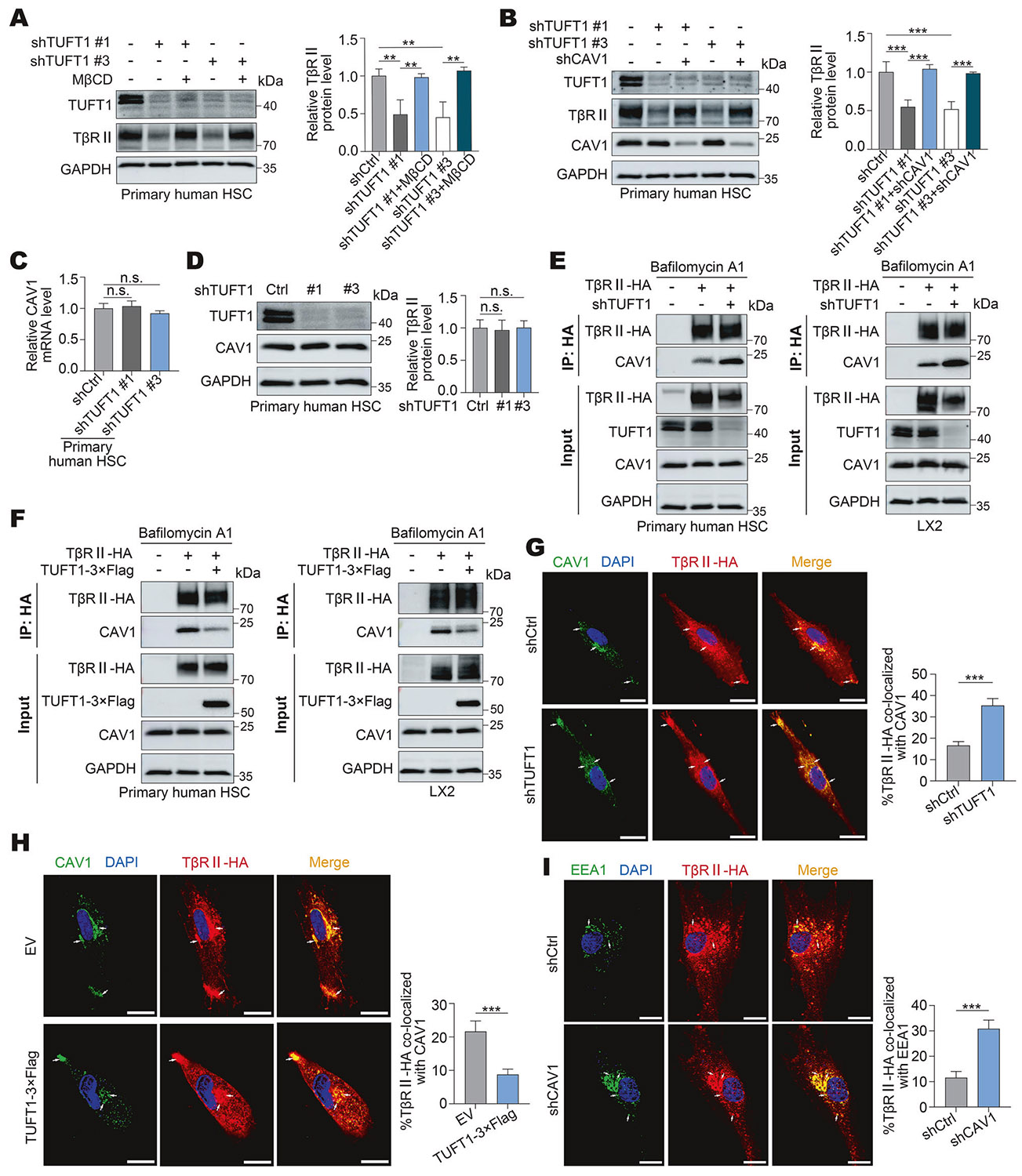
TUFT1 protects TβRII from degradation by competing with CAV1 for binding to TβRII. **A** Control HSCs and TUFT1 knockdown HSCs were incubated with Methyl-β-cyclodextrin (MβCD, 1.5 mM) for 4 h and collected for WB for TβRII. The TβRII protein levels affected by TUFT1 knockdown were restored by MβCD. **P < 0.01 by ANOVA, *n* = 3. **B** HSCs with knockdown of TUFT1 or knockdown of both TUFT1 and caveolin-1 (CAV1) were subjected to WB for TβRII. The TβRII protein levels affected by TUFT1 knockdown were restored by CAV1 knockdown. ***P < 0.001 by ANOVA, *n* = 3. **C**, **D** qRT-PCR and WB revealed that both mRNA and protein levels of CAV1 were not affected by TUFT1 knockdown in HSCs. Not significant (n.s., *P* > 0.05) by ANOVA, *n* = 3. **E** Primary human HSCs (left) and LX2 cells (right) with or without co-expression of TβRII-HA and a TUFT1 shRNA were subjected to co-IP with anti-HA to detect binding of TβRII-HA to CAV1. TβRII-HA /CAV1 binding in the cells was enhanced by TUFT1 knockdown. Data represent 3 independent repeats with similar results. **F** Co-IP revealing TβRII/ CAV1 binding in primary human HSCs (left) and LX2 cells (right) was reduced by overexpression of TUFT1. Data represent 3 independent repeats with similar results. **G** Double IF for TβRII-HA (red) and CAV1 (green) was performed for control and TUFT1 knockdown HSCs. TβRII-HA/CAV1 co-localization (yellow, arrows) was higher in TUFT1 knockdown HSCs compared to control HSCs. Scale bar, 20 μm. ***P < 0.001 by t-test, *n* = 12 cells per group. **H** Double IF revealing TβRII-HA/CAV1 co-localization (yellow, arrows) was reduced in TUFT1 overexpressing HSCs compared to control HSCs. Scale bar, 20 μm. ***P < 0.001 by t-test, *n* = 12 cells per group. **I** Double IF for TβRII-HA (red) and EEA1 (green) revealed that TβRII-HA/EEA1 co-localization (yellow, arrows) was higher in CAV1 knockdown HSCs compared to control HSCs. Scale bar, 20 μm. ***P < 0.001 by t-test, *n* = 12 cells per group.

It has been reported that lipid rafts/caveolae-mediated endocy- tosis downregulates TGF-β receptors and inhibits TGF-β signaling [44-46]. We therefore analyzed the role of lipid rafts/caveolae in TβRII degradation in TUFT1 knockdown cells. Methyl-β- cyclodextrin (MβCD) is an agent that removes cholesterol from the cell membrane and disrupts the lipid rafts of the cells. WB for cells incubated with or without MβCD (1.5 mM) revealed that MβCD effectively restored the TβRII protein level affected by TUFT1 knockdown in both primary human HSCs and LX2 cells (P < 0.01, Fig. 4A and S7A). Since CAV1 is one of the major structural components of lipid rafts/caveolae, we also knocked down CAV1 using shRNA (knockdown effect of CAV1 shRNA lentiviruses was validated by qRT-PCR and WB in Fig. S6A, B, P < 0.001). Consistent with the effect of MβCD, disrupting CAV1 by shRNA effectively restored the TβRII protein levels affected by TUFT1 knockdown in both primary human HSCs and LX2 cells (P < 0.01, Fig. 4B and S7B). Thus, TβRII downregulation induced by TUFT1 targeting is mediated by lipid rafts/caveolae.

### TUFT1 protects TβRII from degradation by competing with CAV1 for binding to TβRII

To gain additional insights into TβRII downregulation in TUFT1 knockdown cells, WB and qRT-PCR were performed for CAV1, which revealed that neither the mRNA nor protein level of CAV1 was influenced by TUFT1 knockdown (P > 0.05, Figs. 4C, D and S7C, D). The data led us to speculate that TUFT1 knockdown may lead to increased TβRII/CAV1 binding so as to sort more TβRII into the lipid rafts/ caveolae degradation pathway. To test this hypothesis, cells incubated with bafilomycin A1 were collected for co-IP for CAV1/TβRII binding, which revealed that TUFT1 knockdown enhanced CAV1/TβRII binding (Fig. 4E), whereas TUFT1 overexpression suppressed CAV1/TβRII binding in both primary human HSCs and LX2 cells (Fig. 4F). Double IF confirmed that TβRII-HA/CAV1 co-localization was elevated in TUFT1 knockdown HSCs, whereas it was reduced in TUFT1- overexpressing HSCs (P < 0.001, Fig. 4G, H). Since endosomes not only mediate the TGF-β signaling but also facilitate receptor trafficking and recycling, double IF for TβRII-HA and early endosome antigen 1 (EEA1; a marker of early endosomes) was performed for CAV1 knockdown HSCs. Confocal microscopy demonstrated that knocking down CAV1 indeed led to higher TβRII/EEA1 and TUFT1/EEA1 colocalization in HSCs (P < 0.001, Figs. 4I and S7E). Together, these data suggest that TUFT1 may prevent TβRII degradation by competing with CAV1 for binding to TβRII to retrieve TβRII from the lipid rafts/ caveolae-mediated degradation pathway into an endosome- mediated trafficking and signaling pathway.

### TUFT1 knockdown alters the TGF-β transcriptome of HSCs

**Fig. 5:**
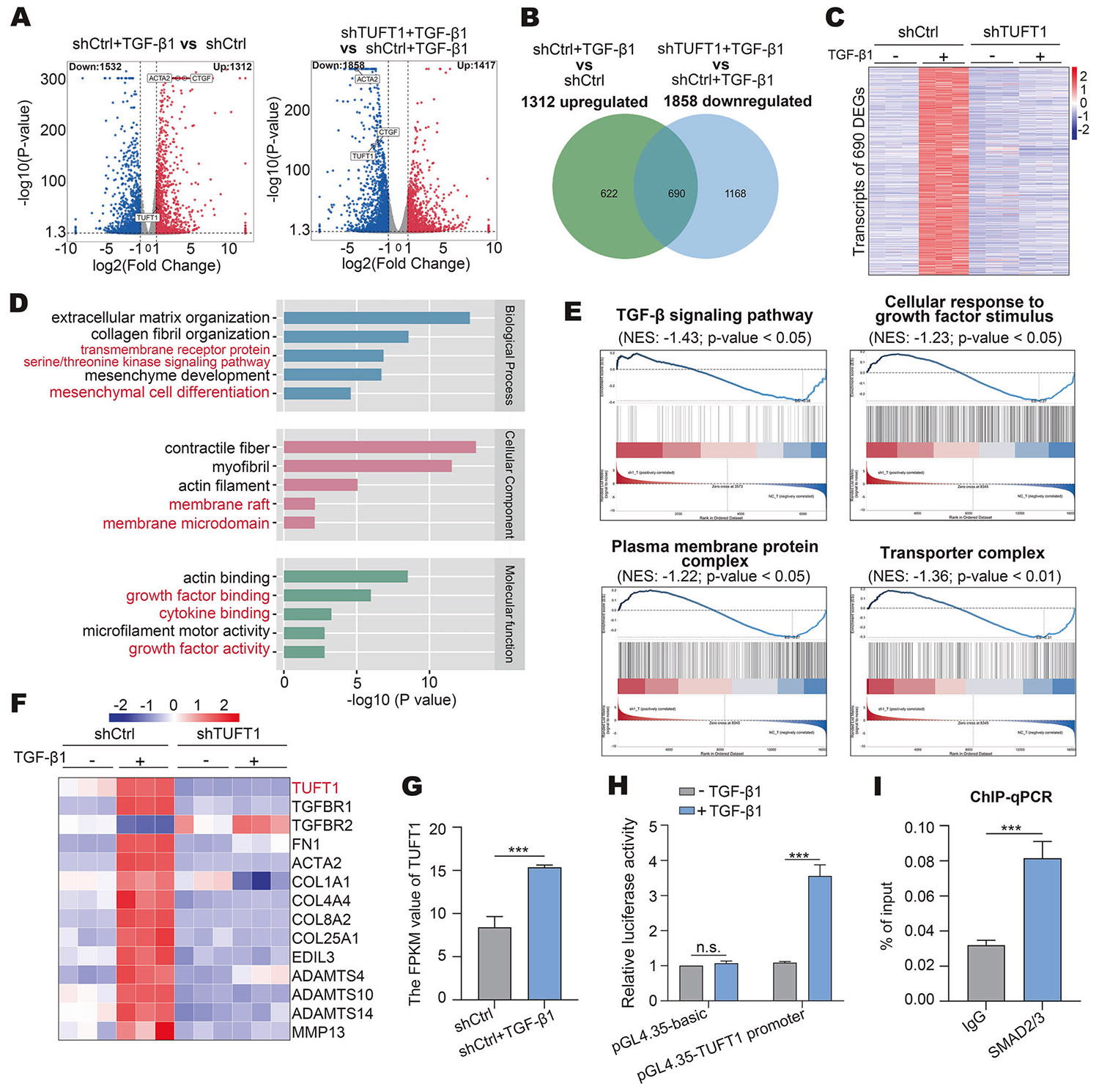
Effect of TUFT1 knockdown on TGF-β1-mediated gene transcription in HSCs and activation of TGF-β/SMAD signaling induces TUFT1 expression. **A** Volcano plots showing differentially expressed genes between the two groups. ∣log2 fold change∣ >1, *P* < 0.05. **B** Venny diagram revealing 690 TUFT1-dependent TGF-β1-inducible transcriptional targets. ∣log2 fold change∣ >1, *P* < 0.05. **C** A heatmap revealing expression levels of 690 differentially expressed genes (DEGs) affected by TUFT1 knockdown. ∣log2 fold change∣ >1, *P* < 0.05. The minimum expression level (blue) to maximum expression level (red) is shown by a color bar. **D** Enrichment analysis revealed that alterations of the expression of 690 DEGs potentially affect biological processes, cellular components, and molecular functions of the cells. Analyzed by R package “clusterProfiler”. **E** Gene Set Enrichment Analysis (GSEA) identified 4 pathways with their gene expression levels affected by TUFT1 knockdown in TGFβ1-stimulated HSCs. NES scores and P values are shown in the plots. **F** A heatmap showing that the transcripts of a gene set related to HSC activation and extracellular matrix remodeling are impacted by TUFT1 knockdown. The minimum expression level (blue) to maximum expression level (red) is shown by a color bar. **G** RNA sequencing revealed that TGF-β1 stimulation increased the transcripts of TUFT1 in HSCs. ***P < 0.001 by t-test, *n* = 3. FPKM: fragments per kilobase per million mapped fragments. **H** Luciferase reporter assay showed that the promoter activity of *TUFT1* was elevated upon TGF-β1 stimulation. Not significant (n.s., *P* > 0.05), ***P < 0.001 by t-test, *n* = 3. **I** ChIP-qPCR showed that SMAD2/3 protein bound to the promoter of *TUFT1* in primary human HSCs under TGF-β1 stimulation. ***P < 0.001 by t-test, *n*=3.

We have previously reported that TGF-β1 stimulation led to the alteration of thousands of gene transcripts of HSCs [42, 47]. To investigate how targeting TUFT1 affected the TGF-β transcriptome of HSCs, bulk-cell RNA sequencing was performed for control and TUFT1 knockdown HSCs (SRA number: PRJNA1165333), revealing that 1312 transcripts of HSCs were upregulated by TGF-β1 (Fig. 5A left, ∣log2 fold change∣ >1, P < 0.05), and that 1858 transcripts in TGF-β1-stimulated HSCs were altered upon TUFT1 knockdown (Fig. 5A right, ∣log2 fold change∣ >1, P < 0.05). The overlap of the two gene sets led to the identification of 690 differentially expressed genes (DEGs) as TGF-β1 targets that were affected by TUFT1 knockdown (Fig. 5B, C). GO enrichment analysis indicated that the DEGs were involved in numerous biological processes, such as ECM organization, transmembrane receptor protein serine/threonine kinase signaling pathway, mesenchyme development, and mesenchymal cell differentiation (Fig. 5D). Cellular component analysis showed that the DEGs were enriched in the contractile fiber, membrane raft, and membrane microdomain, and their molecular function was closely related to actin binding, growth factor binding, cytokine binding, and growth factor activity (Fig. 5D). Moreover, Gene Set Enrichment Analysis (GSEA) revealed that the transcripts in the TGF-β signaling pathway, cellular response to growth factor stimulus, plasma membrane protein complex, and transporter complex were impacted by TUFT1 knockdown (Fig. 5E). The transcripts ofa gene set related to HSC activation and ECM remodeling were also altered by TUFT1 knockdown (Fig. 5F). Thus, targeting TUFT1 leads to a global change in the TGF-β transcriptome of HSCs.

### TUFT1 is required for activation of HSCs into myofibroblasts induced by TGF-β1

**Fig. 6:**
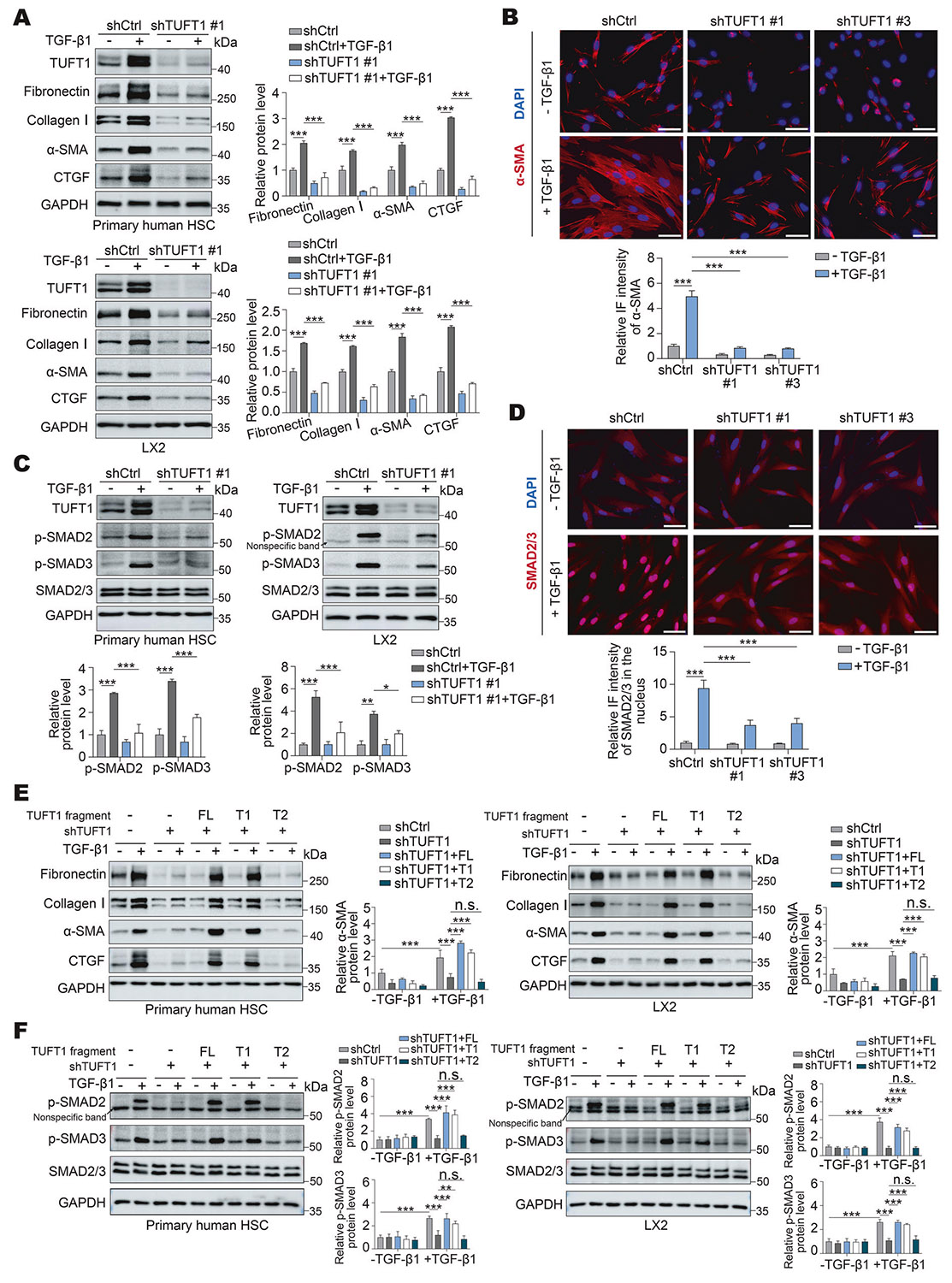
TUFT1 knockdown suppresses TGF-β1–induced activation of HSCs into myofibroblasts. **A** Primary human HSCs (upper) or LX2 cells (lower), with or without TUFT1 knockdown, were incubated with TGF-β1 (5 ng/mL) for 24 h and collected for WB to detect expression of HSC activation markers. TGF-β1-mediated HSC activation was suppressed by TUFT1 knockdown. ***P < 0.001 by ANOVA, *n* = 3. **B** α-SMA IF conducted with primary human HSCs revealed that TGF-β1-mediated myofibroblastic activation of HSCs was suppressed by TUFT1 knockdown. Scale bar, 50 μm. ***P < 0.001 by ANOVA, *n* = 9 randomly picked microscopic fields with each containing more than 50 cells. **C** Cells as described in (**A**) were incubated without or with TGF-β1 (5 ng/mL) for 30 min and collected for WB for phosphorylation of SMAD2 (p-SMAD2) and phosphorylation of SMAD3 (p-SMAD3). TGF-β1-induced SMAD phosphorylation was suppressed by TUFT1 knockdown. *P < 0.05, **P < 0.01, ***P < 0.001 by ANOVA, *n* = 3. **D** IF revealed that TGF-β1 stimulation for 30 min led to nuclear accumulation of SMAD2/3 in HSCs, which was attenuated by TUFT1 knockdown. Scale bar, 50 μm. ***P < 0.001 by ANOVA, *n* = 9 randomly picked microscopic fields with each containing more than 50 cells. **E** The FL TUFT1 or one of the 2 TUFT1 mutants was reintroduced into TUFT1 knockdown primary human HSCs (left) and TUFT1 knockdown LX2 cells (right), followed by TGF-β1 stimulation of the cells. WB revealed that the cell expression of HSC activation markers affected by TUFT1 knockdown was restored by the FL TUFT1 or the T1 mutant, but not by the T2 mutant. Not significant (n.s., *P* > 0.05), ***P < 0.001 by ANOVA, *n* = 3. **F** Cells as described in (**E**) were stimulated with TGF-β1 for 30 min. WB analysis showed that p-SMAD2 and p-SMAD3, affected by TUFT1 knockdown, were restored by the FL TUFT1 or the T1 mutant, but not by the T2 TUFT1 mutant. Not significant (n.s., *P* > 0.05), **P < 0.01, ***P < 0.001 by ANOVA, *n*=3.

Since TβRII is essential for the initiation of the TGF-β signaling, we next investigated the role of TUFT1 on myofibroblastic activation of HSCs in vitro. To this end, primary human HSCs and LX2 cells and their TUFT1 knockdown counterparts were incubated with TGF-β1 (5 ng/mL) for 24 h, and WB was performed to assess cell expression of stellate cell activation markers, α-SMA, CTGF, fibronectin, and type I collagen. As revealed by WB, the protein levels of α-SMA, CTGF, fibronectin, and type I collagen were markedly increased upon TGF-β1 stimulation in control cells, and this effect of TGF-β1 was suppressed by TUFT1 knockdown in both primary human HSCs and LX2 cells (P< 0.001, Figs. 6A and S8A, B). α-SMA IF revealed that in control HSCs, TGF-β1 promoted cell expression of α-SMA and formation of α-SMA-positive stress fibers, characteristics of myofibroblastic activation of HSCs (P < 0.001, Fig. 6B). In contrast, the effect of TGF-β1 was suppressed in TUFT1 knockdown cells (P < 0.001, Fig. 6B). More importantly, we found that TUFT1 knockdown reduced TGF-β1-induced expression of α- SMA, CTGF, fibronectin, and type I collagen, whereas this suppression was reversed by TβRII-HA overexpression in TUFT1 knockdown cells (P < 0.05, Fig. S9A). Thus, shRNA-mediated TUFT1 knockdown suppresses TGF-β1-induced myofibroblastic activation of HSCs in a TβRII-dependent manner.

Cells incubated with TGF-β1 for 30 min were also collected for WB to determine the influence of TUFT1 knockdown on SMAD phosphorylation induced by TGF-β1. In both primary human HSCs and LX2 cells, knocking down TUFT1 suppressed phosphorylation of SMAD2 and SMAD3 induced by TGF-β1 (P< 0.05, Figs. 6C and S8C, D). IF demonstrated that TGF-β1 stimulation led to the accumulation of SMAD2/3 in the nucleus of control HSCs, which was drastically reduced by TUFT1 knockdown (P < 0.001, Fig. 6D). Further, the protein levels of p-SMAD2 and p-SMAD3 induced by TGF-β1 were rescued by TβRII-HA overexpression in TUFT1 knockdown cells (P < 0.001, Fig. S9B). Moreover, overexpression of the FL TUFT1 or the T1 truncation mutant (a.a. 1–157 of TUFT1) restored TGF-β1-stimulated cell expression of the stellate cell activation markers and SMAD2/3 phosphorylation affected by TUFT1 knockdown in both primary human HSCs and LX2 cells (P < 0.01, Fig. 6E, F). Thus, TUFT1 is required for TβRII- dependent SMAD signaling activation and myofibroblastic differentiation of HSCs upon TGF-β1 stimulation.

### TUFT1 is transcriptionally upregulated by TGF-β1 stimulation

Both RNA sequencing and WB revealed that TUFT1 was upregulated by TGF-β1 stimulation (Figs. 5A, G, 6A, and S10A). We therefore collected primary human HSCs and LX2 cells, stimulated with TGF-β1 for 24 h, for qRT-PCR, and the results confirmed that TUFT1 mRNA level was induced by TGF-β1 in both HSCs and LX2 cells, similar to that of α-SMA and CTGF (P < 0.05, Fig. S10B). To test whether TUFT1 is a direct transcriptional target of TGF-β1/SMAD signaling, a TUFT1 luciferase reporter construct was created for luciferase reporter assay by inserting the promoter of TUFT1 (–1000 bp to 0 bp) into the luciferase reporter vector pGL4.35-basic. The empty vector was used as a control. Luciferase reporter assay revealed that luciferase expression under the TUFT1 promoter in 293T cells was enhanced to more than 3-fold by TGF- β1 stimulation (P < 0.001, Fig. 5H). Anti-SMAD2/3 was next used for ChIP, followed by qPCR to test whether SMAD2/3 of HSCs were able to bind to the promoter of TUFT1 in response to TGF-β1 stimulation. Our ChIP-qPCR results indeed confirmed that SMAD2/3 bound to the promoter region of TUFT1 in TGF-β1-stimulated HSCs (P < 0.001, Fig. 5I). Thus, the TUFT1 gene is directly turned on for gene transcription by the TGF-β/SMAD signaling in HSCs.

### Higher TUFT1 expression in the CAFs compared to quiescent HSCs

Next, we leveraged publicly available scRNA-seq data to compare Tuft1 expression in the CAFs of murine CRC liver metastases to that in quiescent HSCs at a single-cell resolution. To this end, GSM4874984 datasets from the GSE160541 series [37] were downloaded from Gene Expression Omnibus for analyzing Tuft1 transcripts with the R toolkit Seurat. Based on the sequencing data, the cells in the murine CRCLM were grouped into 6 clusters by the software: T cells, endothelial cells, monocytes, macrophages, fibroblasts, and hepatocytes (Fig. S11A). Cells in the fibroblast group were further clustered and separated into 2 scattered groups (Fig. S11B). Gene expression signature of quiescent HSCs, such as expression of transcripts of Lrat, Gfap, and Des, stratified Lrat^+^Gfap^+^Des^+^ cells as quiescent HSCs (Fig. S11C); Gene expression signature of CAFs, such as expression of transcripts of Acta2 and Col1a1, stratified Acta2^+^Col1a1^+^ as the CAFs (Fig. S11D). Profiling of Tuft1 transcripts at the single-cell level revealed that the number of Tuft1 transcripts per cell was higher in the CAFs compared to quiescent HSCs (P< 0.001, Fig. S11E, F). In addition to analyzing RNA levels, we measured protein levels of TUFT1 in CRCLM samples of the patients. In the end, the CAFs of patient CRCLM and matched HSCs in the adjacent normal liver tissues were isolated from 18 CRC patients. WB revealed that the TUFT1 protein level was significantly higher in the CAFs compared to matched HSCs (P< 0.001, Fig. S12). Moreover, the expression protein level of TβRⅡ was also upregulated in the CAFs than in adjacent HSCs (P< 0.001, Fig. S12).

### Targeting TUFT1 suppresses the tumor-promoting effect of HSCs in vitro

**Fig. 7:**
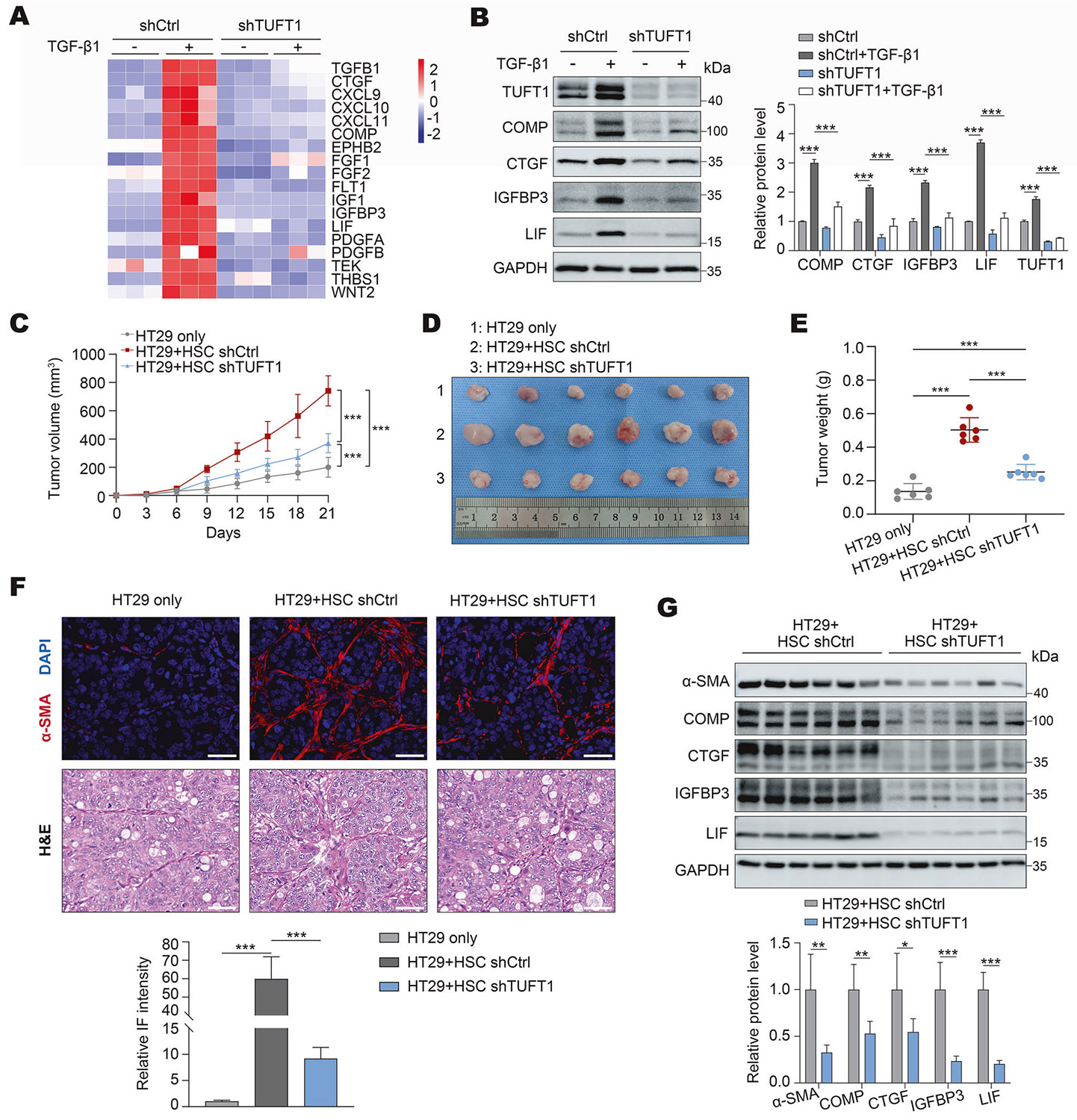
TUFT1 knockdown suppresses the tumor-promoting effect of HSCs in mice. **A** RNA sequencing revealed that TUFT1 knockdown suppressed HSC expression of tumor-promoting factors in response to TGF-β1 stimulation for 24 h. FDR *P* < 0.05. The minimum expression level (blue) to maximum expression level (red) is shown by a color bar. **B** WB confirmed that TGF-β1-stimulated expression of 4 tumor-promoting factors was suppressed by TUFT1 knockdown in HSCs. ***P < 0.001 by ANOVA, *n* = 3. **C**, **D**, **E** Subcutaneous co-injection of human HT29 CRC cells with either control or TUFT1 knockdown HSCs into nude mice revealed that the tumor-promoting effect of HSCs was reduced by TUFT1 knockdown. HT29 tumor growth curves are shown in (**C**). ***P < 0.001 by ANOVA, *n* = 6. The pictures of HT29 tumors at the endpoint are shown in (**D**), and tumor weights are shown in (**E**). ***P < 0.001 by ANOVA, *n* = 6. **F** α-SMA IF detected lower CAF densities in tumors arising from HT29+HSCshTUFT1 co-injections compared to control co-injections. Scale bar, 50 μm. ***P < 0.001 by ANOVA, *n* = 6. **G** WB showed that the levels of HSC-derived tumor-promoting factor were reduced in tumors arising from HT29 + HSC shTUFT1 co-injections compared to control co-injections. *P< 0.05, **P < 0.01, ***P < 0.001 by t-test, *n* = 6.

Our RNA sequencing data also revealed that a panel of transcripts encoding growth factors and cytokines was altered by TUFT1 knockdown in HSCs, including CXCL9, CXCL10, FGF2, IGF1, IGFBP3, LIF, PDGFA, PDGFB, COMP, and THBS1, suggesting that TUFT1 may regulate the tumor-promoting effects of HSCs (Fig. 7A). qRT-PCR and WB confirmed that TGF-β1 indeed promoted HSC expression of various tumor-promoting factors, such as COMP, CTGF, IGFBP3, and LIF, in a TUFT1-dependent manner (P< 0.001, Fig. 7B, and Fig. S15A). These data led us to use the CM of HSCs for in vitro experiments to test whether TUFT1 knockdown influenced the tumor-promoting effect of HSCs. To this end, human CRC cells, HT29 and HCT116 cells, were incubated with a CM collected from control HSCs or TUFT1 knockdown HSCs. Subsequently, they were subjected to CCK8 proliferation assay, colony-forming assay, Edu assay, and transwell-based migration and invasion assays. Cells incubated with the basal medium were used as the control. As expected, the CM of control HSCs and that of TUFT1 knockdown HSCs promoted proliferation, migration, and invasion of HT29 and HCT116 cells compared to the basal medium (P < 0.05, Fig. S13A-D). However, the CM of TUFT1 knockdown HSCs was less effective on CRC cells compared to that of control HSCs (P < 0.05, Fig. S13A-D). WB also revealed that the CM of control HSCs prompted cancer cell expression of N-cadherin and vimentin, and suppressed CRC expression of E-cadherin (P < 0.01, Fig. S13E). Moreover, these effects of HSC CM were suppressed by TUFT1 knockdown (P < 0.01, Fig. S13E). Notably, introducing TβRII- HA into TUFT1 knockdown HSCs partially rescued the impaired tumor-promoting effect of TUFT1 knockdown HSCs (P < 0.05, Fig. S14A-E). Thus, targeting HSC TUFT1 suppresses the tumorpromoting effect of HSCs in vitro by downregulating TβRII protein.

### Targeting TUFT1 of HSCs suppresses CRC tumor growth in a subcutaneous tumor implantation mouse model

Next, we employed a CRC/HSCs co-implantation mouse model to test the role of HSC TUFT1 in CRC cell growth in mice [17, 18]. To this end, HT29 (1 × 10^6^ cells) were mixed with either control HSCs or TUFT1 knockdown HSCs (1 × 10^6^ cells) in vitro, followed by their co-injection into nude mice subcutaneously. Tumor growth curves were generated by measuring tumor nodules once every 3 days (Fig. 7C-E), revealing that co-injection of control HSCs or TUFT1 knockdown HSCs promoted HT29 tumor growth in mice compared to HT29 injection alone. However, TUFT1 knockdown HSCs showed reduced efficacy compared to control HSCs. As revealed by IHC, the rate of Ki67-positive tumor cells was lower in the tumors arising from HT29/HSC-shTUTF1 co-injections compared to tumors arising from control co-injections (P < 0.01, Fig. S15B, C). E-cadherin IHC signals were stronger, whereas vimentin IHC signals were weaker in the tumors arising from HT29/HSC-shTUTF1 co-injections compared to tumors arising from control co-injections (P < 0.01, Fig. S15B, C). IF for α-SMA demonstrated that the CAF densities were reduced in tumors arising from HT29/HSC-shTUFT1 co-injections compared to tumors arising from control co-injections (P < 0.001, Fig. 7F). Moreover, the levels of HSC-derived tumor-promoting factors, such as COMP, CTGF, IGFBP3, and LIF, were all reduced in tumors arising from HT29/HSC-shTUFT1 co-injections (P < 0.05, Fig. 7G). Moreover, the inhibitory effect caused by TUFT1 knockdown of HSCs on CRC tumor growth, HSC activation, and HSC expression of tumorpromoting factor was partially abolished by TβRII-HA overexpression (P < 0.05, Fig. S16A-E). Thus, targeting TUFT1 of HSCs selectively suppresses CAF activation of HSCs and CRC tumorigen- esis in a subcutaneous tumor implantation mouse model via reducing TβRII protein expression.

### Targeting HSC TUFT1 by Cre/loxP recombination suppresses CAF activation of HSCs and CRCLM in mice

**Fig. 8:**
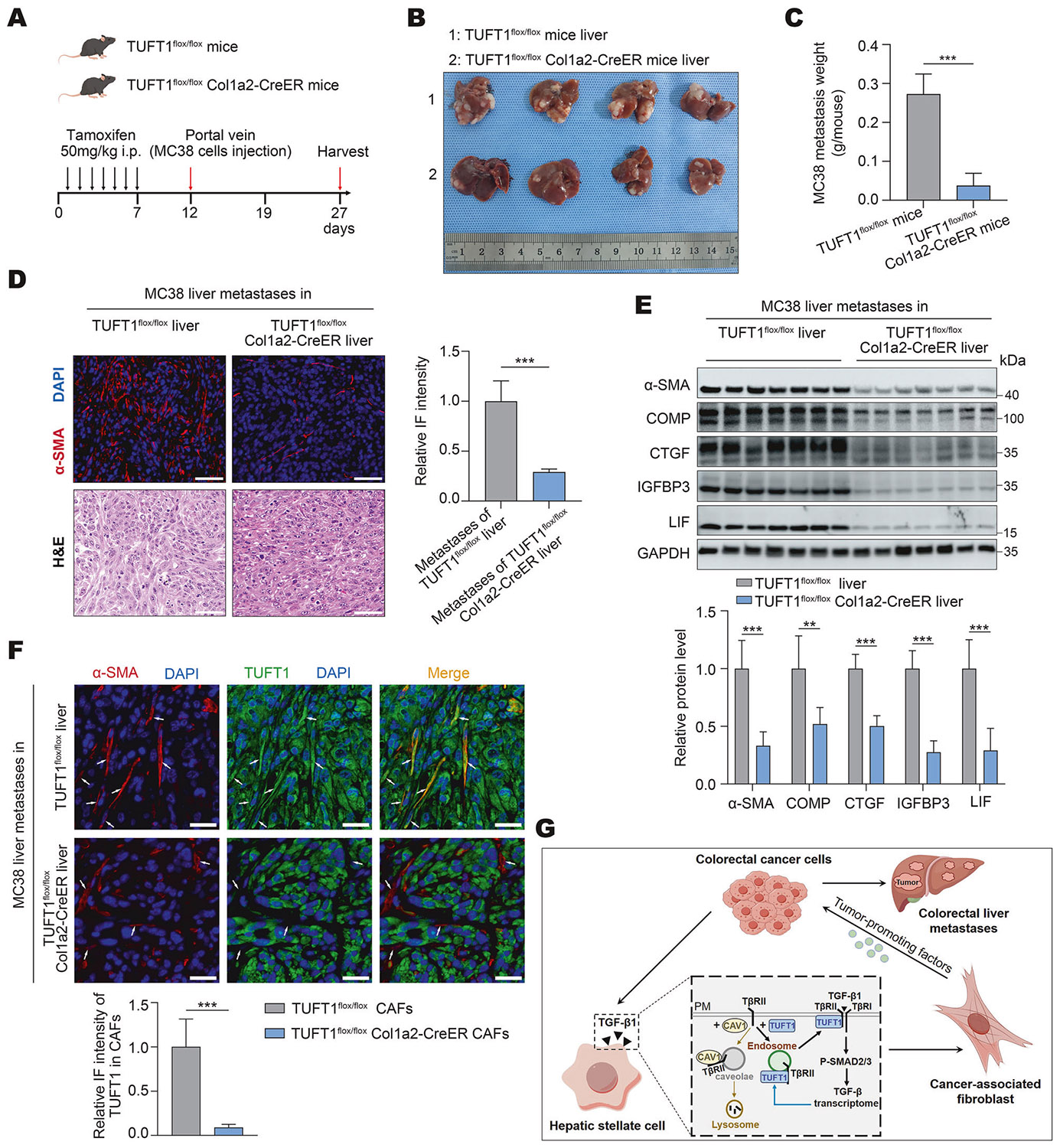
Targeting HSC TUFT1 by Cre/LoxP recombination suppresses HSC activation and CRCLM in mice. **A** A schematic representation of the study to generate CRCLM in control and conditional TUFT1 knockout mice. **B**, **C** Mice were sacrificed at day 15 after portal vein injection of MC38 CRC cells. Representative pictures of murine livers at the endpoint are shown in (**B**), and the weight of MC38 CRCLM is shown in (**C**). ***P < 0.001 by t-test, *n* = 7. **D** α-SMA IF revealed that the CAF density of MC38 liver metastasis (LM) was lower in TUFT1^flox/flox^Col1a2-CreER mice than in TUFT1^flox/flox^ mice. Scale bar, 50 μm. ***P < 0.001 by t-test, *n* = 7. **E** WB detected that the levels of 4 tumor-promoting factors were all reduced in MC38 LM of TUFT1^flox/flox^Col1a2-CreER mice compared to those of TUFT1^flox/flox^ mice. **P < 0.01, ***P < 0.001 by t-test, *n*=7. **F** Double IF for α-SMA and TUFT1 demonstrated much reduced TUFT1 IF in TUFT1^flox/flox^Col1a2-CreER CAFs compared to TUFT1^flox/flox^ CAFs of the MC38 LM. Scale bar, 25 μm. ***P < 0.001 by t-test, *n* = 28, 21. **G** A diagram summarizing the mechanism by which TUFT1 of HSCs protects TβRII from degradation in HSCs and facilitates TGF-β1-mediated activation of HSCs into CAFs that promote CRCLM.

To translate our findings into the hepatic tumor microenvironment and CRCLM, we obtained a TUFT1 floxed mutant mouse line from Cyagen Biosciences (Suzhou, China) and set up its crossing with a Col1a2-CreER transgenic mouse line from Cyagen Biosciences (Suzhou, China). Age-matched TUFT1^flox/flox^ mice (control) and TUFT1^flox/flox^Col1a2-CreER mice were selected for portal vein tumor injection (PCR-based mouse genotyping results are shown in Fig. S17A). As shown in the experimental scheme in Fig. 8A, each mouse received tamoxifen injection intraperitoneally once daily for 7 consecutive days to induce activation of the Cre recombinase in mice (50 mg/kg/day), and portal vein injection of murine MC38 cells was performed at day 12 (5 × 10^5^ cells per mouse). Mice were sacrificed 15 days later, which revealed that TUFT1^flox/flox^Col1a2-CreER mice developed fewer MC38 liver metastases compared to TUFT1^flox/flox^ mice (Figs. 8B, C and S17B). IF detected lower α-SMA IF densities in MC38 liver metastases of TUFT1^flox/flox^Col1a2-CreER mice compared to those of TUFT1^flox/flox^ mice (P < 0.001, Fig. 8D). WB revealed that the protein levels of α- SMA, COMP, CTGF, IGFBP3, and LIF were all reduced in the liver metastases of TUFT1^flox/flox^Col1a2-CreER mice compared to those of TUFT1^flox/flox^ mice (P < 0.01, Fig. 8E). As expected, much reduced TUFT1 IF signals were detected in TUFT1^flox/flox^Col1a2-CreER CAFs compared to TUFT1^flox/flox^ CAFs (P < 0.001, Fig. 8F). Thus, targeting HSC TUFT1 by Cre/LoxP-mediated gene deletion suppresses CAF activation of HSCs and CRCLM in mice.

## DISCUSSION

The hepatic microenvironment provides a fertile soil for the dissemination and seeding of CRC cells [48]. Within this niche, HSCs have emerged as pivotal regulators, significantly contributing to forming a pro-metastatic environment [49]. Upon activation, HSCs facilitate CRCLM through multiple mechanisms, including ECM remodeling, angiogenesis, potentiation of EMT, and immune suppression [9]. Unraveling the mechanistic basis of HSC activation and its functional contribution to CRCLM thus becomes a critical focus for the current study. Here, we showed that TUFT1 facilitates TGF-β1-induced activation of HSCs into metastasis-promoting myofibroblasts by protecting TβRII from lipid rafts/caveolae-mediated degradation (Fig. 8G). Functionally, knocking down TUFT1 of HSCs attenuates CAF activation of HSCs and suppresses CRCLM in mice. Additionally, TUFT1 is transcriptionally upregulated by the TGF-β/SMAD signaling of HSCs (Fig. 8G), and its expression level is higher in the CAFs of CRCLM compared to quiescent HSCs. Collectively, our findings establish TUFT1 as a promising therapeutic target to disrupt the transformation of HSCs into CAFs and inhibit CRCLM.

The activation of HSCs by TGF-β is tightly controlled at the receptor level. The binding of TGF-β to TβRII triggers the signaling cascade, positioning this receptor as the essential gatekeeper that orchestrates downstream events [50]. The regulation of TβRII turnover is crucial for TGF-β signaling. Deubiquitinases such as POH1, USP8, and USP11 bind directly to TβRII, deubiquitinating and stabilizing it to potentiate TGF-β signaling [51-53]. Similarly, c- Cbl-mediated neddylation of TβRII prevents its ubiquitination and subsequent degradation [54]. While binding of IQGAP1 to TβRII enhances SMURF1/TβRII co-localization, leading to lysosomal and proteasomal degradation of TβRII, focal adhesion kinase (FAK) or programmed death-ligand 1 (PD-L1) targets TβRII onto the PM to protect TβRII from degradation by lysosomes [17, 41, 42]. Despite these insights, further investigation is needed into TβRII- interacting proteins in the context of HSC activation. TUFT1 plays a vital role in tooth development and mineralization, and its wider expression in non-mineralized normal and tumor tissues points to additional biological functions [21, 25]. Accumulating evidence has verified the oncogenic role of TUFT1, implicating it in promoting proliferation, metastasis, and drug resistance of tumor cells [27, 29, 30]. However, little is known about the expression of TUFT1 in HSCs or its tumor-promoting effects within the tumor microenvironment. This study reveals TUFT1 expression and function in HSCs, highlighting its novel role in shaping the hepatic tumor microenvironment. In addition, for the first time, we discovered that TUFT1 is a TβRⅡ-binding protein in HSCs, providing a mechanistic basis for its function. The interaction between TUFT1 and TβRII regulates TβRII protein stability and intracellular trafficking. TUFT1 mediates TβRII stabilization through its specific regions (a.a. 1–86 and a.a. 87–157), protecting it from lysosomal degradation. Ubiquitination is recognized as an essential signal that directs the trafficking of TβRII from the PM to the lysosome for degradation [17, 41, 42]. In TUFT1 knockdown cells, the level of ubiquitinated TβRⅡ is elevated and undergoes lysosomal degradation. Consequently, the amount of TβRⅡ recycling back to the PM from the cytoplasm is diminished. These findings emphasize the critical role of TUFT1 in HSCs through binding to TβRII. Furthermore, our finding that TUFT1 controls the sorting mechanism of TβRII may have an important implication and relevance for sorting many PM proteins and secretory proteins critical for tumor and tumor stromal cell functions.

TGF-β receptors are known to be internalized via clathrin- mediated and lipid rafts/caveolae-mediated pathways [55]. The clathrin-dependent endocytosis carries TGF-β receptors to the early endosomes for SMAD activation, and the receptors can also be transported back to the PM via Rab11-positive recycling endosomes [18, 56]. In contrast, the lipid rafts/caveolae-depen- dent endocytosis leads to receptor degradation [45, 46, 51]. Although the two pathways are well-recognized, the mechanisms by which TβRII is sorted into one or another, or retrieved from one into another, remain inadequately understood. Interestingly, herein we observed that disrupting lipid rafts with MβCD rescues TβRII protein levels that were downregulated upon TUFT1 knockdown. Knockdown of CAV1, the critical scaffolding and regulatory protein in lipid rafts/caveolae, also rescued TβRII expression in TUFT1-deficient HSCs. As the first-identified and best-characterized member of the caveolin family, CAV1 is essential for caveolae formation and regulates critical cellular processes such as endocytosis and signal transduction [57]. The consistent effects of MβCD treatment and CAV1 knockdown strongly support the conclusion that TUFT1 inhibits the lipid rafts/ caveolae-mediated trafficking of TβRII to lysosomal degradation. Further mechanistic studies demonstrated that TUFT1 competitively disrupts CAV1 binding to TβRII and retrieves TβRII from the caveolae into the early endosome. Thus, TUFT1 and CAV1 compete for binding to TβRII, which is a critical determinant of the fate of TβRII, whether TβRII is sorted into the clathrin-mediated recycling and signaling pathway or into the lipid rafts/caveolae- mediated degradation pathway.

It is well-established that the responsiveness of HSCs to TGF-β is regulated by TβRII [41, 42]. Our study identified TUFT1 as a critical requisite for TGF-β1 signaling and the subsequent activation of HSCs into metastasis-promoting CAFs. We also demonstrated that TUFT1 mediates the TGF-β1-induced myofibroblastic activation of HSCs in a TβRII-dependent manner. Furthermore, consistent with prior research [40], we found that TUFT1 expression is upregulated by the TGF-β1/SMAD pathway, thereby establishing a positive feedback loop that potently reinforces HSC activation. Activated HSCs remodel the hepatic microenvironment by secreting many factors, including cytokines, growth factors, and ECM components [5]. Among these, tumor-promoting factors such as CTGF, COMP, LIF, and IGFBP3 are known to drive cancer proliferation and metastasis [31, 58-60]. Our data revealed that TUFT1 modulates the expression of this key set of tumor-promoting factors in HSCs and mediates the promotion of CRC cell proliferation, migration, invasion, and EMT by HSCs. Notably, targeting HSC TUFT1 significantly inhibits the growth of CRC subcutaneously and the development of liver metastases, supporting TUFT1 as a key player driving HSC activation and CRCLM in vivo. These findings underscore the significance of TUFT1 within HSC activation to orchestrate the pre-metastatic niche in the liver.

Nevertheless, there are some limitations associated with the current study. Activated-HSCs/CAFs facilitate the recruitment of immunosuppressive cells into the liver microenvironment, which is one of the mechanisms underlying immune escape and evasion of cancer [61, 62]. This study focuses specifically on the role of the myofibroblast TUFT1 for tumor cells. Whether HSC TUFT1 regulates HSC/immune crosstalks to promote CRCLM remains unclear. Additionally, the present study utilized shRNA-mediated knockdown to investigate the functional role of TUFT1 in HSC activation. While this genetic approach provides valuable insights, it has inherent limitations for therapeutic translation. Future development of direct inhibitory agents, such as neutralizing antibodies or targeted peptides against TUFT1, will be essential to validate its druggability and achieve a more precise and therapeutically relevant blockade of its pro-metastatic functions in HSCs. Furthermore, our RNA-seq analysis indicated that TUFT1 depletion leads to broad transcriptomic alterations in HSCs. Although we have identified the stabilization of TβRII as a key mechanism, we cannot entirely exclude the possibility that TUFT1 may interact with additional, yet unidentified, binding partners or modulate non-canonical pathways. Elucidating these potential mechanisms represents a major focus of our ongoing research.

In summary, we have identified a new function of TUFT1 and uncovered a novel mechanism driving CRCLM, whereby TUFT1 of HSCs protects TβRII from degradation by the lipid rafts/caveolae pathway to facilitate TGF-β-mediated CAF activation of HSCs. Targeting HSC TUFT1 suppresses HSC activation and CRCLM in mice. Our results highlight TUFT1 as a potential therapeutic target to disrupt crosstalk between HSCs and CRC cells, offering a novel strategy against CRCLM.

## Supplementary Material

Supplementary information

Suppl. Table 1

Suppl. Table 2

Suppl. Table 3

Suppl. Table 4

Supplementary information The online version contains supplementary material available at https://doi.org/10.1038/s41418-026-01664-2.

## Data Availability

All raw RNA sequencing data were uploaded to the NCBI SRA database (accession No: PRJNA1165333). Other data that support the findings of this study are available from the corresponding authors upon reasonable request.
