# Supplementary material for: TUFT1 stabilizes TGF-β receptor II protein and facilitates activation of hepatic stellate cells into metastasis-promoting myofibroblasts
Source: Cell Death Differ. 2026 Jan 28;33(7):1436–54. doi: 10.1038/s41418-026-01664-2 (PMC13203373; doi:10.1038/s41418-026-01664-2)
Supplement: Supplementary file 1 — Supplementary Figures and Legends [file 41418_2026_1664_MOESM1_ESM.docx]

**Supplementary Figures and Legends**

**
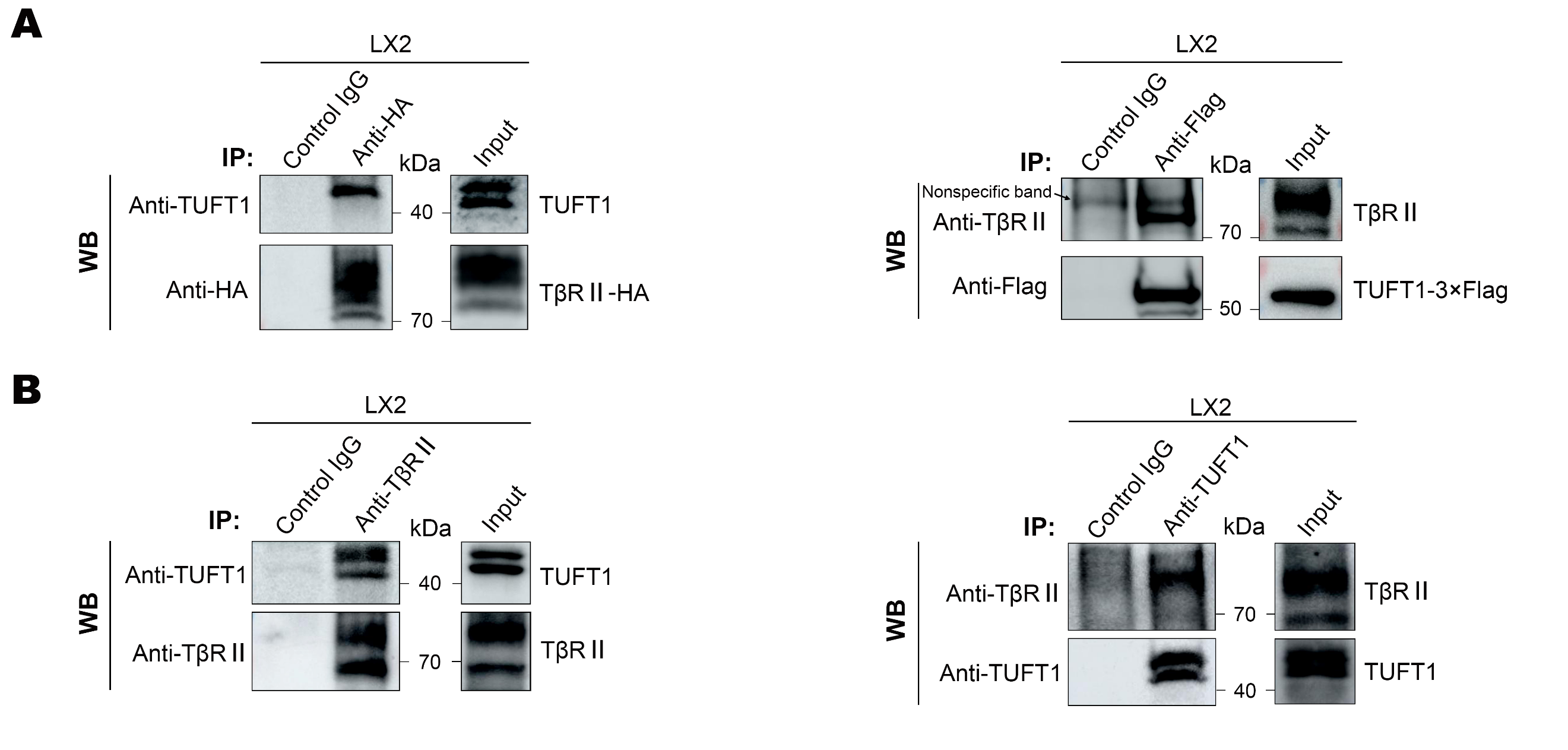
**

**Fig. S1 TUFT1 interacts with TGF-β receptor Ⅱ (TβRⅡ) in LX2 cells. A** LX2 cells overexpressing TβRⅡ-HA (left) or overexpressing TUFT1-3×Flag (right) were collected for co-IP with anti-HA or anti-Flag antibody; co-precipitated TUFT1 or TβRⅡ was detected by Western blot (WB) analysis. Data represent 3 repeats with similar results. **B** Co‐immunoprecipitation (co-IP) confirmed the interaction between endogenous TβRⅡ and TUFT1 in LX2 cells using anti-TβRⅡ antibody (left) or anti-TUFT1 antibody (right). Data represent 3 repeats with similar results.

**
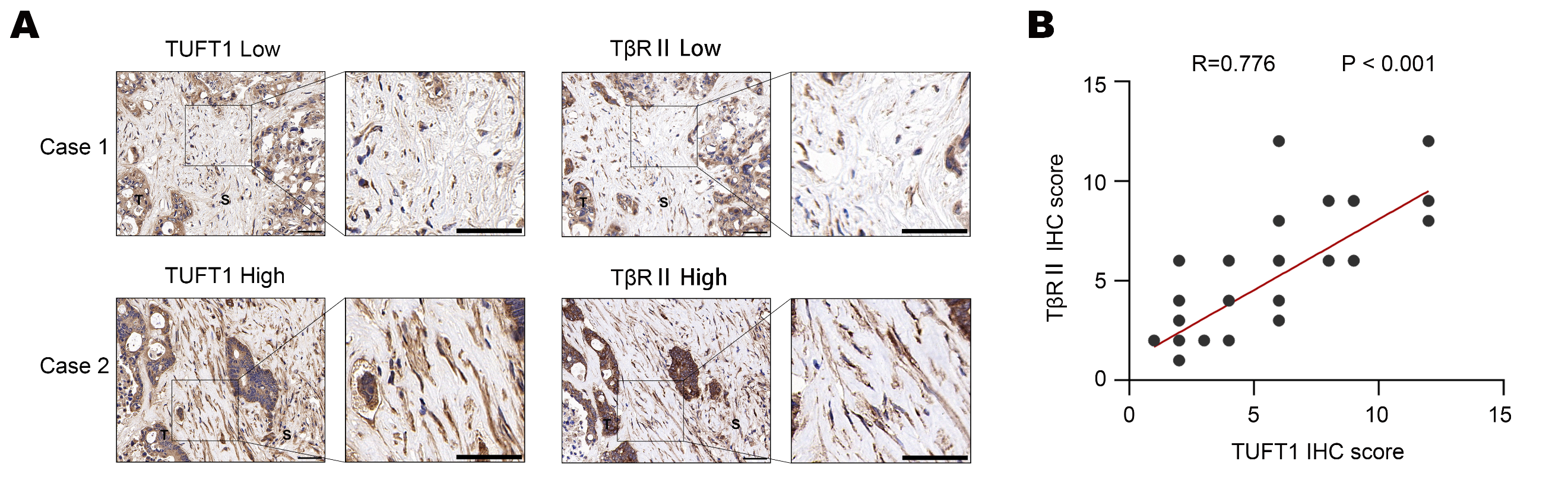
**

**Fig. S2 TUFT1 and TβRⅡ expression are positively correlated in the stroma of CRCLM of the patients. A** Immunohistochemistry (IHC) was performed on patient CRCLM tissue, and representative IHC images for TUFT1 and TβRⅡ are shown. Scale bar, 50 µm. **B** Patient IHC data were subjected to Spearman correlation analysis, which revealed that TUFT1 and TβRII levels were positively correlated in 40 CRCLM samples. R=0.776, P < 0.001.

**
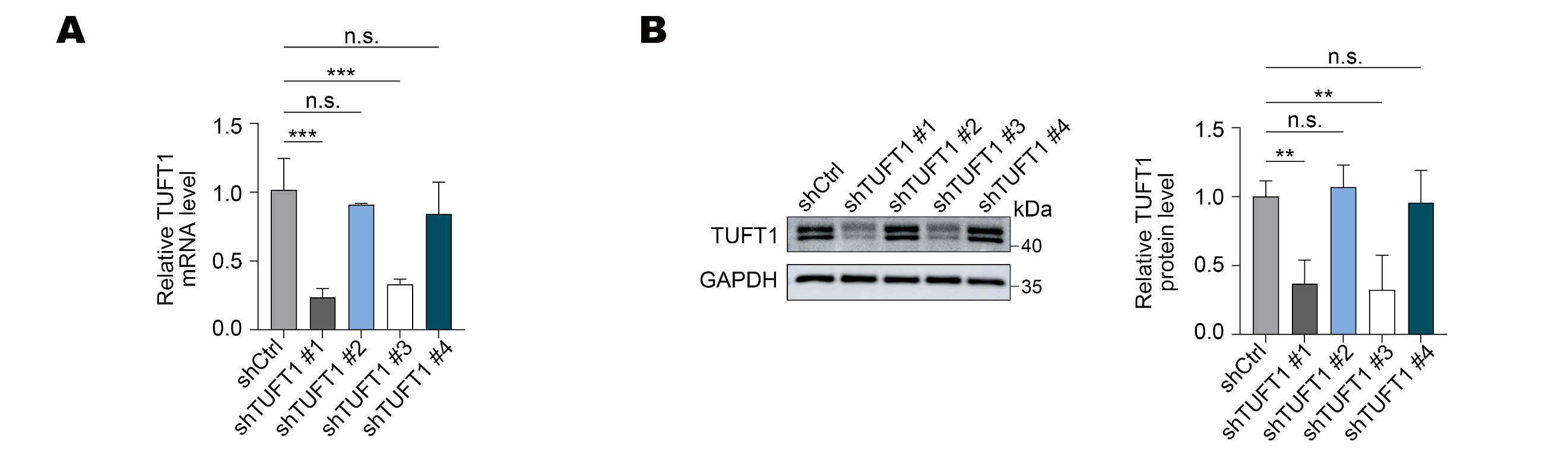
**

**Fig. S3 Validation of the knockdown effect of TUFT1 shRNAs. A** qRT-PCR for TUFT1 was performed on cells transduced with each of 4 different TUFT1 shRNA lentiviruses. Not significant (n.s., P > 0.05), ***P < 0.001 by ANOVA, n = 3. **B** Western blot (WB) validation for TUFT1 knockdown. Not significant (n.s., P > 0.05), **P < 0.01 by ANOVA, n = 3.


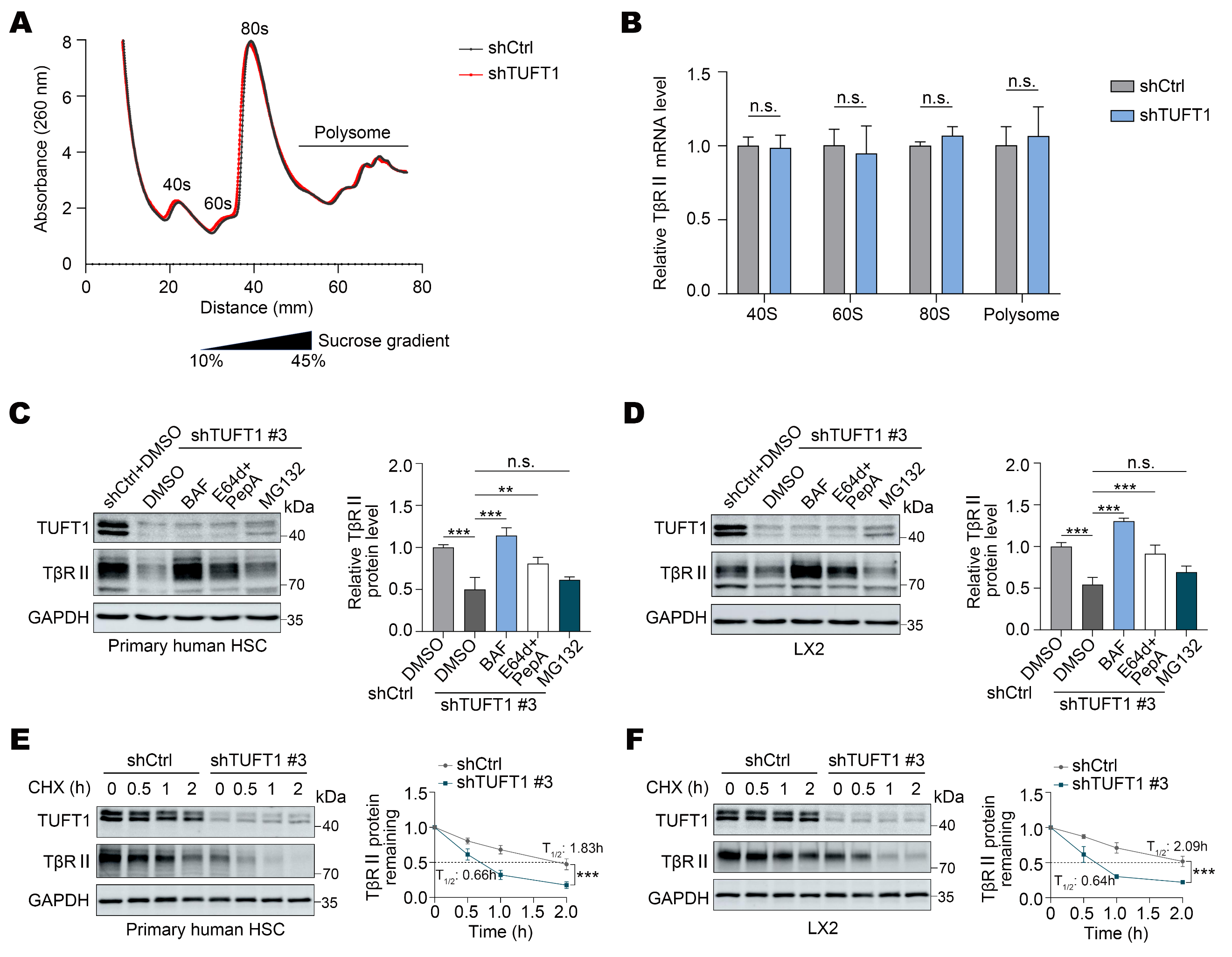


**Fig. S4 TUFT1 knockdown promotes lysosome-mediated degradation of TβRII. A** Polysome profiling by sucrose density gradient showed that the polysome distribution profile in TUFT1 knockdown LX2 cells remained unchanged compared to control cells. **B** Quantification of TβRII mRNA level in each fraction by qRT-PCR demonstrated that TUFT1 knockdown did not alter TβRII mRNA translation. n.s., not significant; P > 0.05 by t-test, n = 3. **C** Primary human HSCs and **D** LX2 cells transduced with TUFT1 shRNA #3 lentiviruses were incubated with bafilomycin A1 [BAF, 10 nM] or E64d [10 μg/mL] + pepstatin A [PepA, 10 μg/mL], or proteasome inhibitor (MG132, 25 μM) for 8 hours were subjected to WB for TβRII. The TβRII protein levels affected by TUFT1 knockdown were restored by the lysosomal inhibitors, but not by the proteasomal inhibitor. Not significant (n.s., P > 0.05), **P< 0.01, ***P < 0.001 by ANOVA, n = 3. **E** TUFT1 shRNA #3 lentiviruses were used to knock down TUFT1 of primary human HSCs and **F** LX2 cells, and the cells were subjected to TβRII degradation analysis by WB. TβRII degraded much faster in TUFT1 knockdown cells compared to control cells. ***P < 0.001 by ANOVA, n = 3.


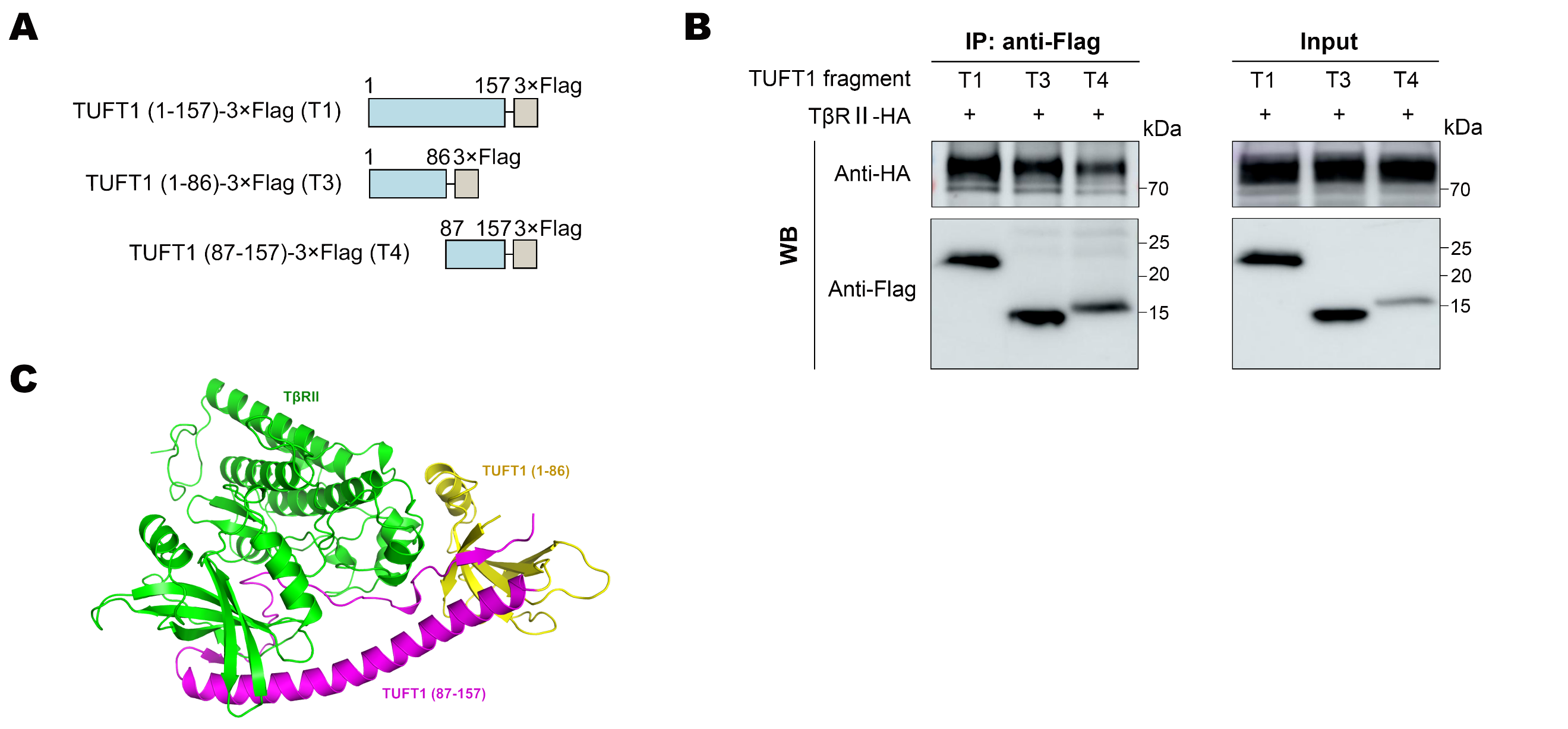


**Fig. S5 TβRII interacts with N-terminal a.a. 1-86 and a.a. 87-157 of TUFT1. A** Schematic diagram for TUFT1 (a.a. 1-157)-3×Flag, TUFT1 (a.a. 1-86)-3×Flag, and TUFT1 (a.a. 87-157)-3×Flag truncation mutants. **B** TβRII-HA was co-transfected with either TUFT1 truncation mutant shown in **A** into 293T cells for co-IP with anti-Flag antibody. Both a.a. 1-86 and a.a. 87-157 fragments of TUFT1 can interact with TβRII. **C** The structural model of the TUFT1 (a.a. 1-157)/TβRII complex was predicted with Boltz-2 (based on AlphaFold3) using default parameters and visualized using PyMOL. In the representation, TUFT1 (a.a. 1-86), TUFT1 (a.a. 87-157), and TβRII are colored in yellow, magenta, and green, respectively.

**
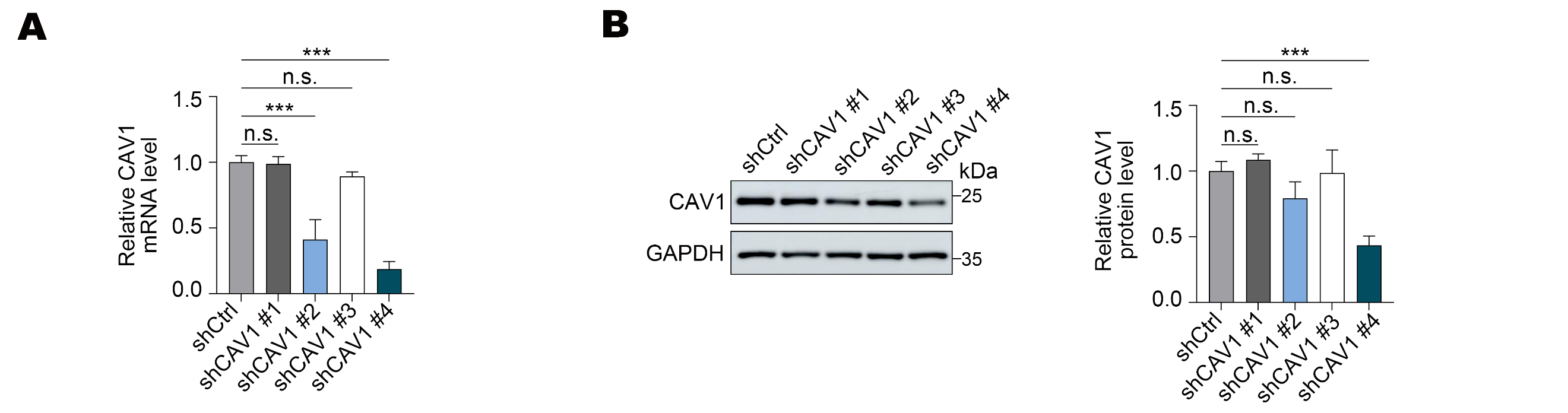
**

**Fig. S6 Validation of the knockdown effect of CAV1 shRNAs. A** qRT-PCR validation of CAV1 knockdown by different shRNA lentiviruses. Not significant (n.s., p > 0.05), ***P < 0.001 by ANOVA, n = 3. **B** WB validation of CAV1 knockdown by different shRNA lentiviruses. Not significant (n.s., P > 0.05), ***P < 0.001 by ANOVA, n = 3.


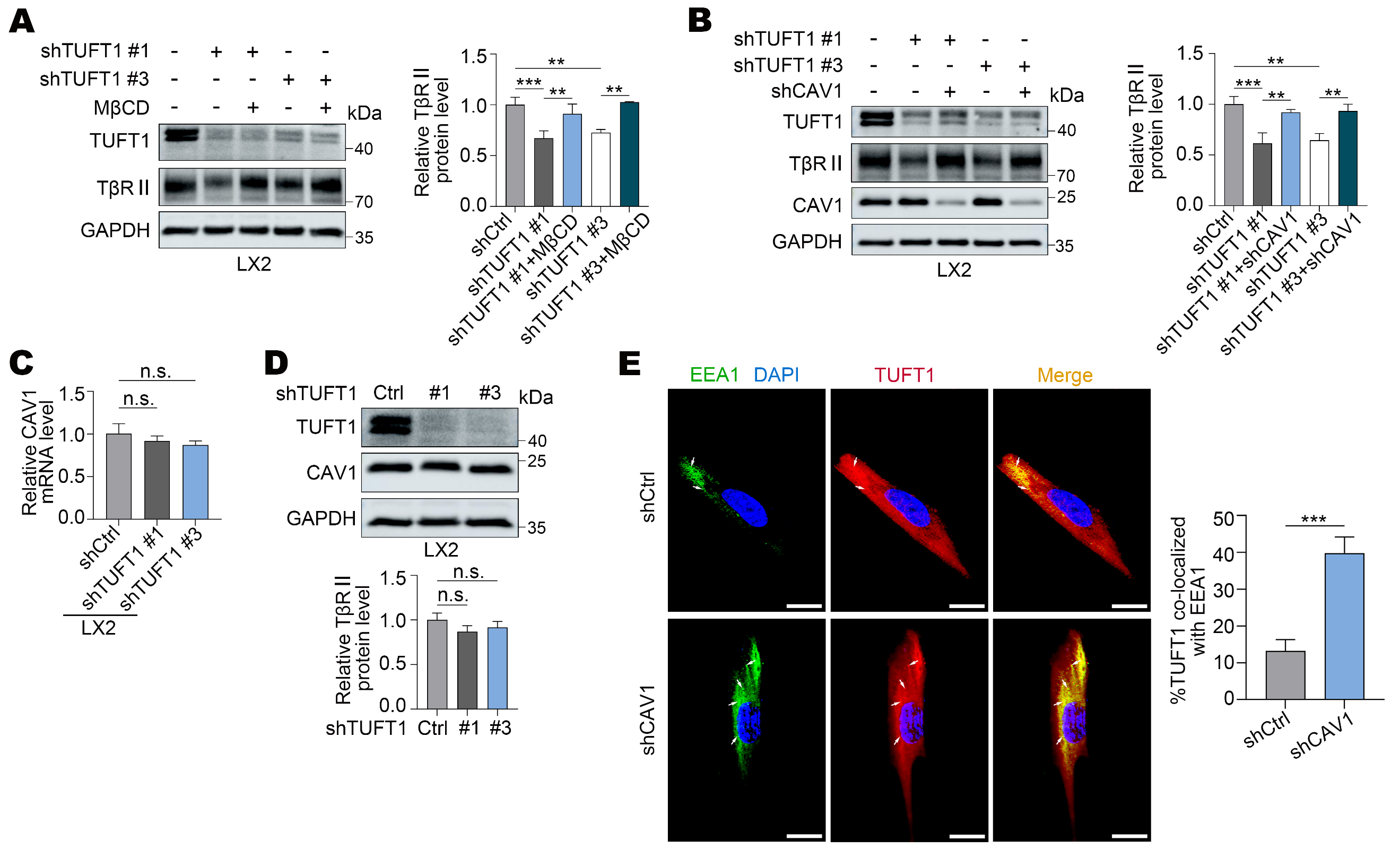


**Fig. S7 Targeting TUFT1 induces lipid rafts/caveolae-mediated degradation of TβRII. A** Control and TUFT1 knockdown LX2 cells were incubated with Methyl-β-cyclodextrin (MβCD, 1.5 mM) for 4 hours and collected for WB for TβRII. The TβRII protein levels affected by TUFT1 knockdown were restored by MβCD in LX2 cells. **P < 0.01, ***P < 0.001 by ANOVA, n = 3. **B** LX2 cells with knockdown of TUFT1 or knockdown of both TUFT1 and caveolin-1 (CAV1) were subjected to WB for TβRII. The TβRII protein levels affected by TUFT1 knockdown were restored by CAV1 knockdown. **P < 0.01, ***P < 0.001 by ANOVA, n = 3. **C, D** qRT-PCR and WB revealed that mRNA and protein levels of CAV1 were not affected by TUFT1 knockdown in LX2 cells. Not significant (n.s., P > 0.05) by ANOVA, n = 3. **E** Double IF staining for TUFT1 (red) and EEA1 (green) showed that the rate of TUFT1/EEA1 co-localization (yellow, arrows) was higher in CAV1 knockdown HSCs compared to control HSCs. Arrows highlight areas of co-localization. Scale bar, 20 μm. ***P < 0.001 by t-test, n = 12.


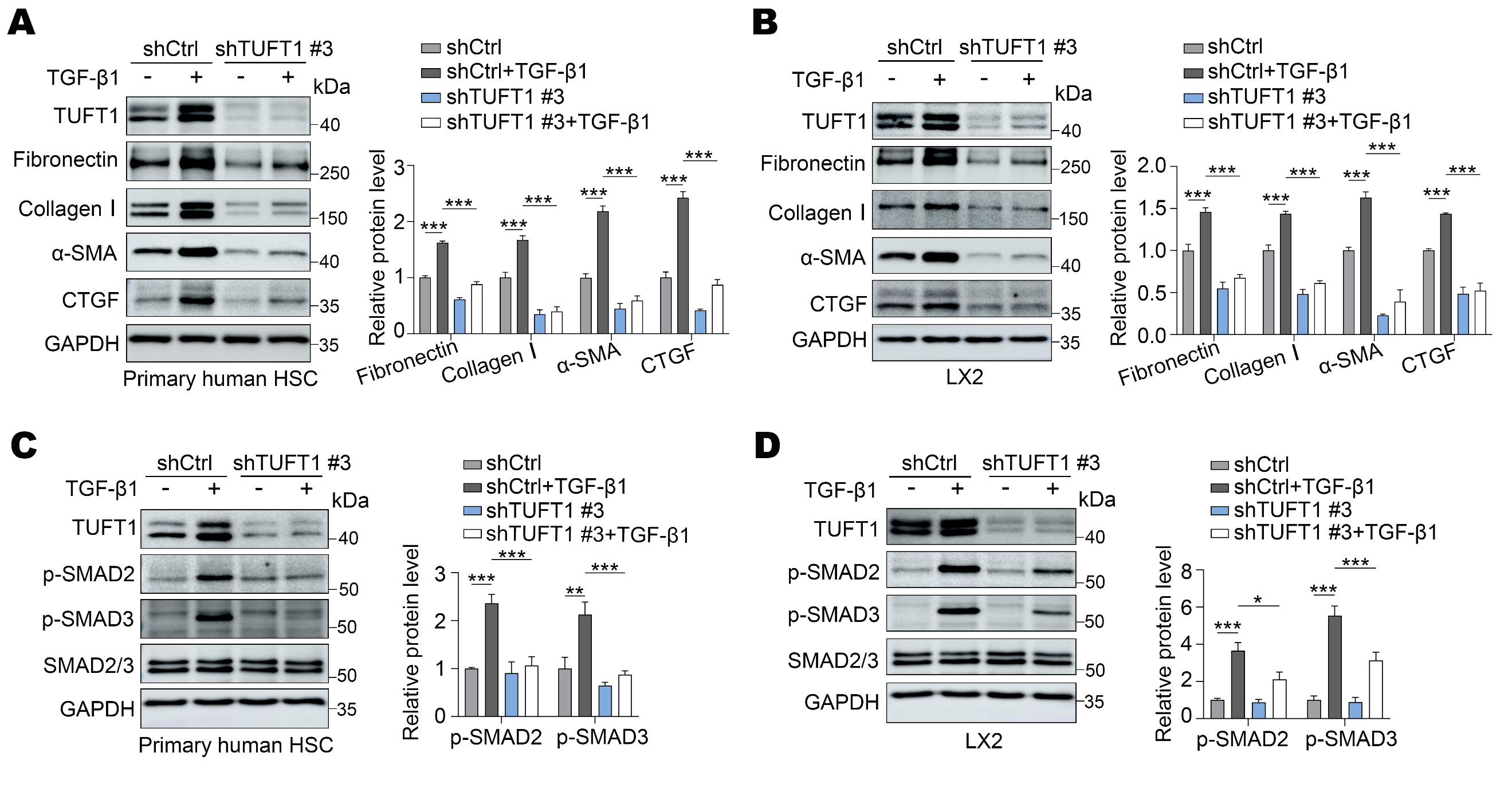


**Fig. S8 TUFT1 knockdown inhibits TGF-β1–induced activation of HSCs into myofibroblasts. A** Primary human HSCs and **B** LX2 cells transduced with shTUFT1 #3 lentiviruses were incubated with TGF-β1 (5 ng/mL) for 24 hours, and WB was performed to detect cell expression of activation markers. TGF-β1-mediated HSC activation was suppressed by TUFT1 knockdown. ***P < 0.001 by ANOVA, n = 3. **C** Primary human HSCs and **D** LX2 cells transduced with shTUFT1 #3 lentiviruses were incubated with TGF-β1 (5 ng/mL) for 30 minutes, and WB was performed to detect phosphorylation of SMAD2 and SMAD3 induced by TGF-β1 in the cells. TGF-β1-induced SMAD2/3 phosphorylation was suppressed by TUFT1 knockdown. *P < 0.05, **P < 0.01, ***P < 0.001 by ANOVA, n = 3.


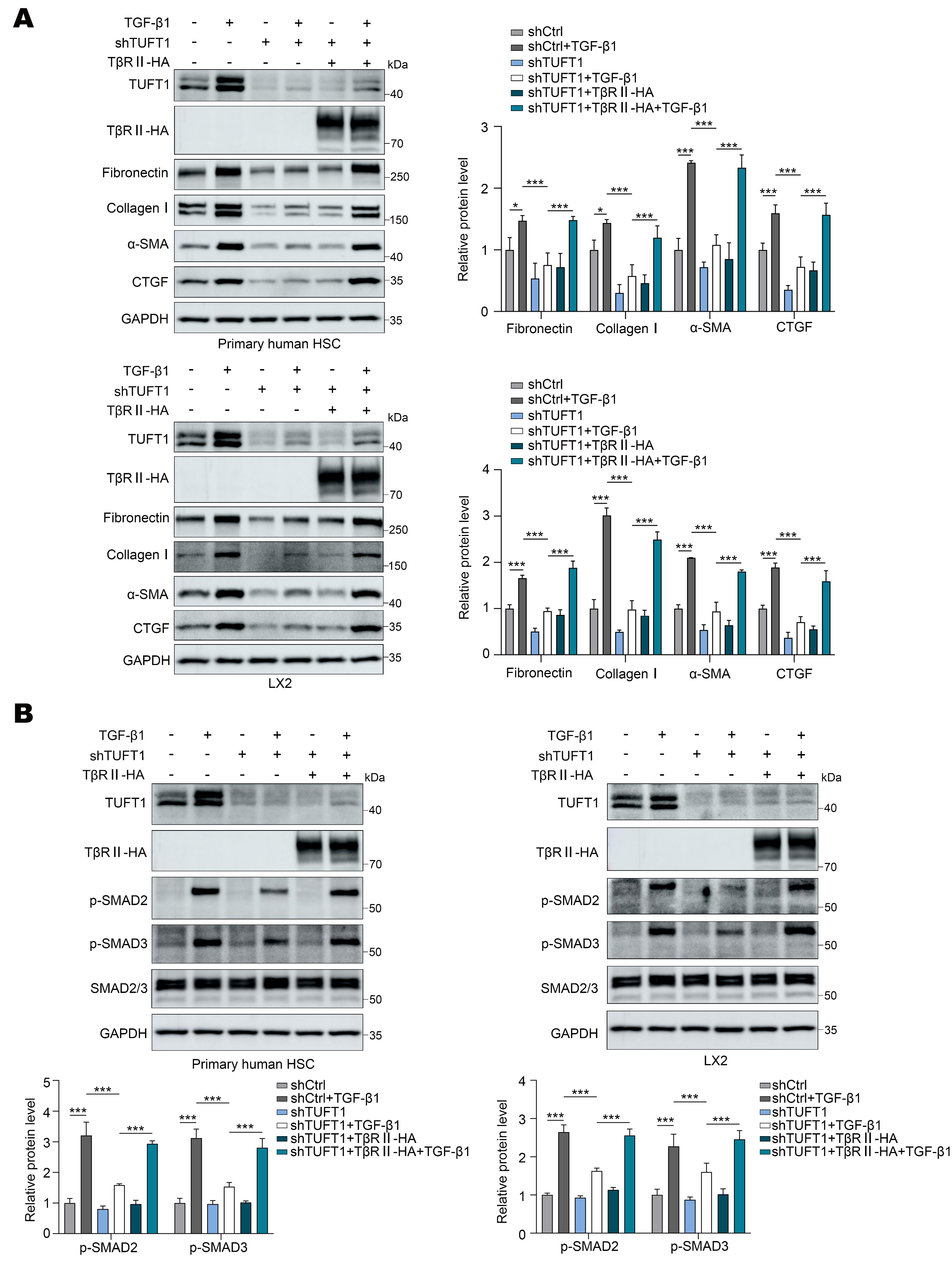


**Fig. S9 TUFT1 promotion of HSC activation into myofibroblasts induced by TGF-β1 is dependent on TβRII. A** TUFT1 knockdown primary human HSCs (upper) and TUFT1 knockdown LX2 cells (lower) were transduced with control or TβRII-HA lentiviruses, followed by TGF-β1 stimulation (5 ng/mL) for 24 hours. WB revealed that the impaired expression of stellate cell activation markers in TGFβ1-stimulated TUFT1 knockdown cells was rescued by TβRII-HA overexpression. *P < 0.05, ***P < 0.001 by ANOVA, n = 3. **B** The cells as described in **A** were incubated with TGF-β1 (5 ng/mL) for 30 minutes. WB revealed that the impaired phosphorylation of SMAD2 and SMAD3 in TGFβ1-stimulated TUFT1 knockdown cells was rescued by TβRII-HA overexpression. ***P < 0.001 by ANOVA, n = 3.


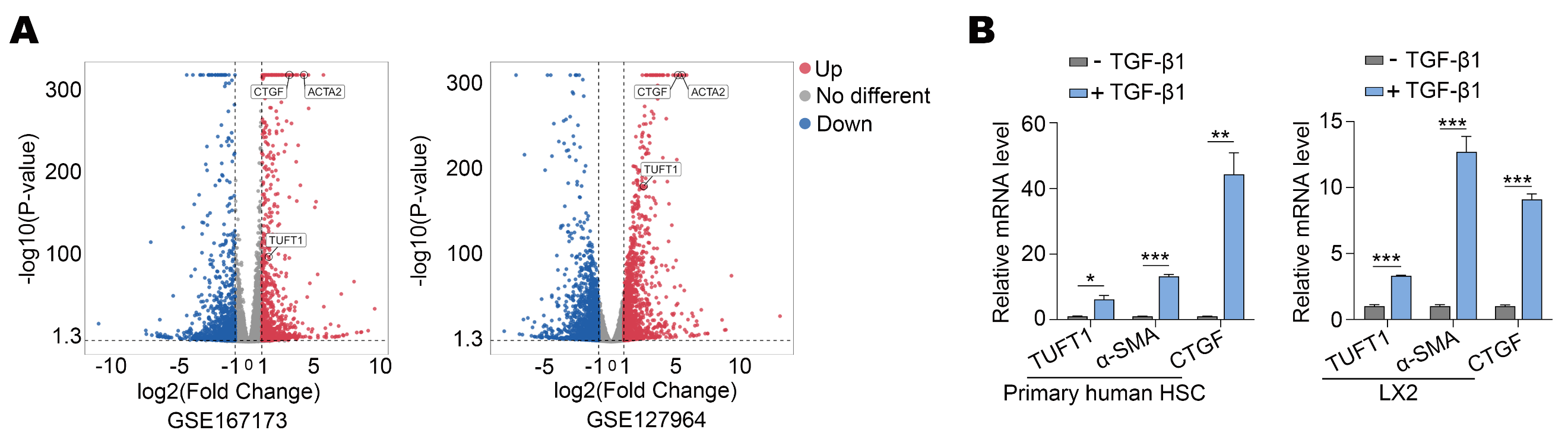


**Fig. S10 TUFT1 expression is induced by the TGF-β/SMAD signaling in HSCs. A** Volcano plots for the GSE167173 and GSE127964 datasets revealed that TUFT1 transcripts were upregulated by TGF-β1 stimulation in primary human HSCs. **B** qRT-PCR confirmed upregulation of TUFT1 transcripts by TGF-β1 stimulation for 24 hours (5 ng/mL) in primary human HSCs (left) and LX2 cells (right). *P < 0.05, **P < 0.01, ***P < 0.001 by t-test, n = 3.


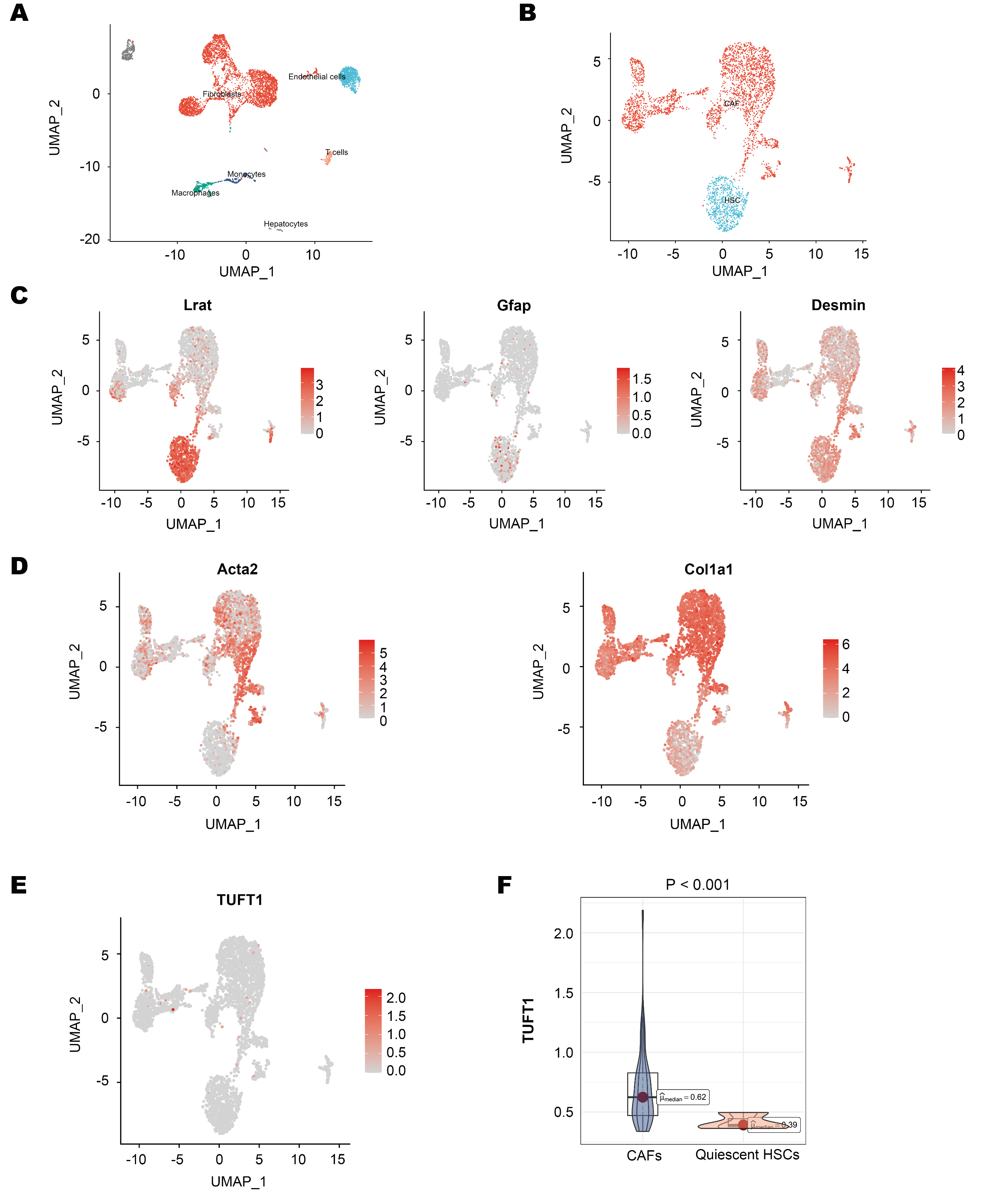


**Fig. S11 Single-cell RNA sequencing revealing higher number of *Tuft1* transcripts in the CAFs of CMT93 murine CRC liver metastases compared to quiescent HSCs.** **A** [GSM4874984](https://www.ncbi.nlm.nih.gov/geo/query/acc.cgi?acc=GSM4874983) datasets of [GSE160541](https://www.ncbi.nlm.nih.gov/geo/query/acc.cgi?acc=GSE160541) in GEO were analyzed by the R toolkit Seurat. The UMAP revealed that the cells were divided into 6 clusters based on their gene expression pattern. **B** The cells of the fibroblast group were further divided into 2 clusters. **C** UMAPs revealing cells expressing the transcripts of *Lrat*, *Gfap*, or *Des*. The redder the color is, the higher the transcript level is. **D** UMAP plots showing cells expressing Acta2 or Col1a1 transcripts. The redder the color is, the higher the transcript level is. **E** An UMAP revealing cells expressing *Tuft1* transcripts. The redder the color is, the higher the transcript level is. **F** Quantification of *Tuft1* transcripts at the single-cell level revealed a higher number of *Tuft1* transcripts in the CAFs compared to quiescent HSCs. P < 0.001, the Wilcoxon signed rank was used to compare the two groups.


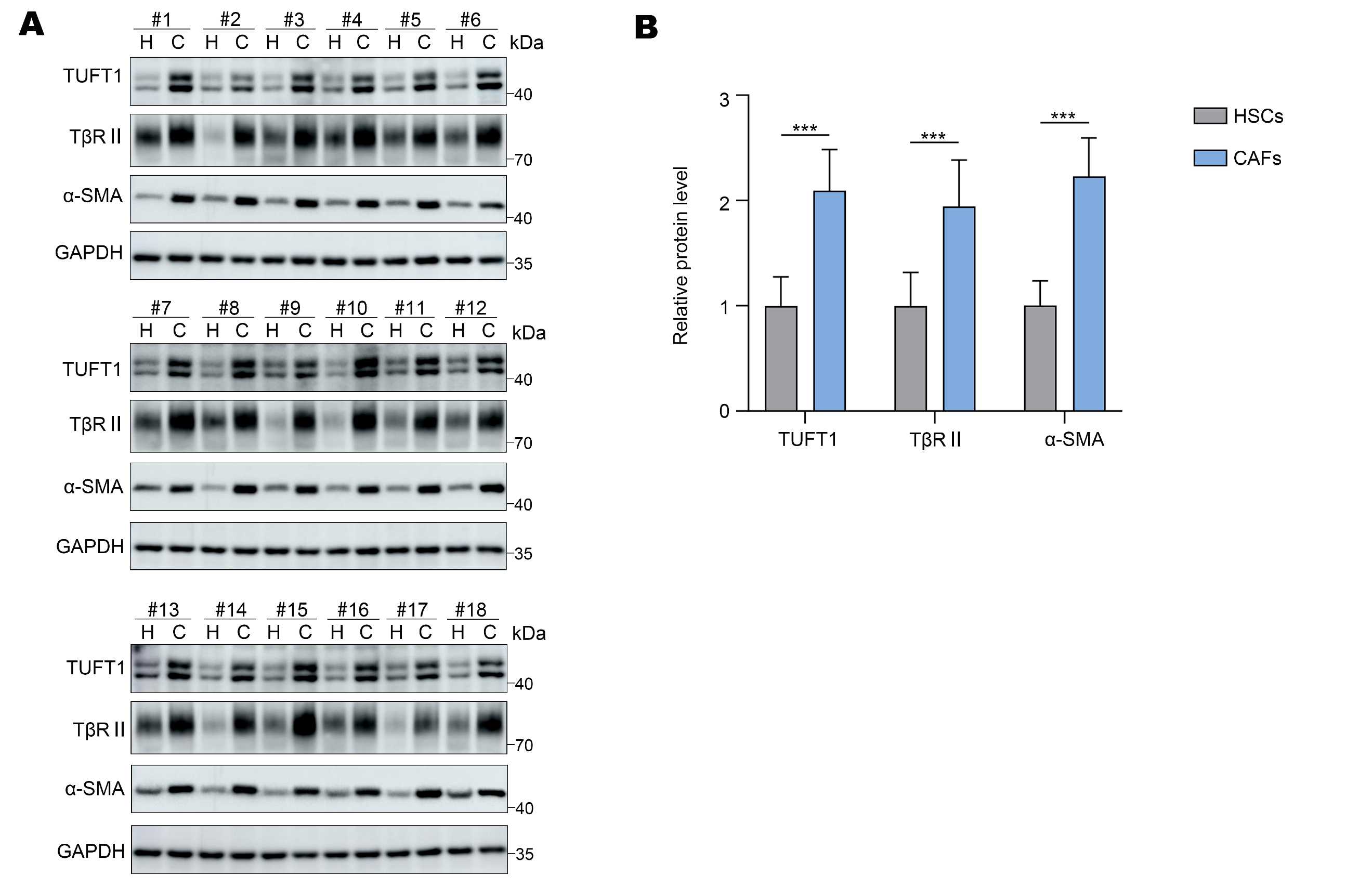


**Fig. S12 Higher TUFT1 and TβRⅡ protein levels were detected in the CAFs of patient CRCLM than in adjacent liver HSCs. A** CAFs of CRCLM and matched HSCs in the adjacent normal liver tissues were isolated from 18 CRC patients. Cells were cultured *in vitro* and collected for WB. As isolated HSCs underwent spontaneous activation during *in vitro* culture, low levels of α-SMA were detected in HSCs by WB. **B** Densitometric analysis revealed that the CAFs of patient CRCLM express higher TUFT1 and TβRⅡ protein levels than matched HSCs. ***P < 0.001 by t-test, n = 18.


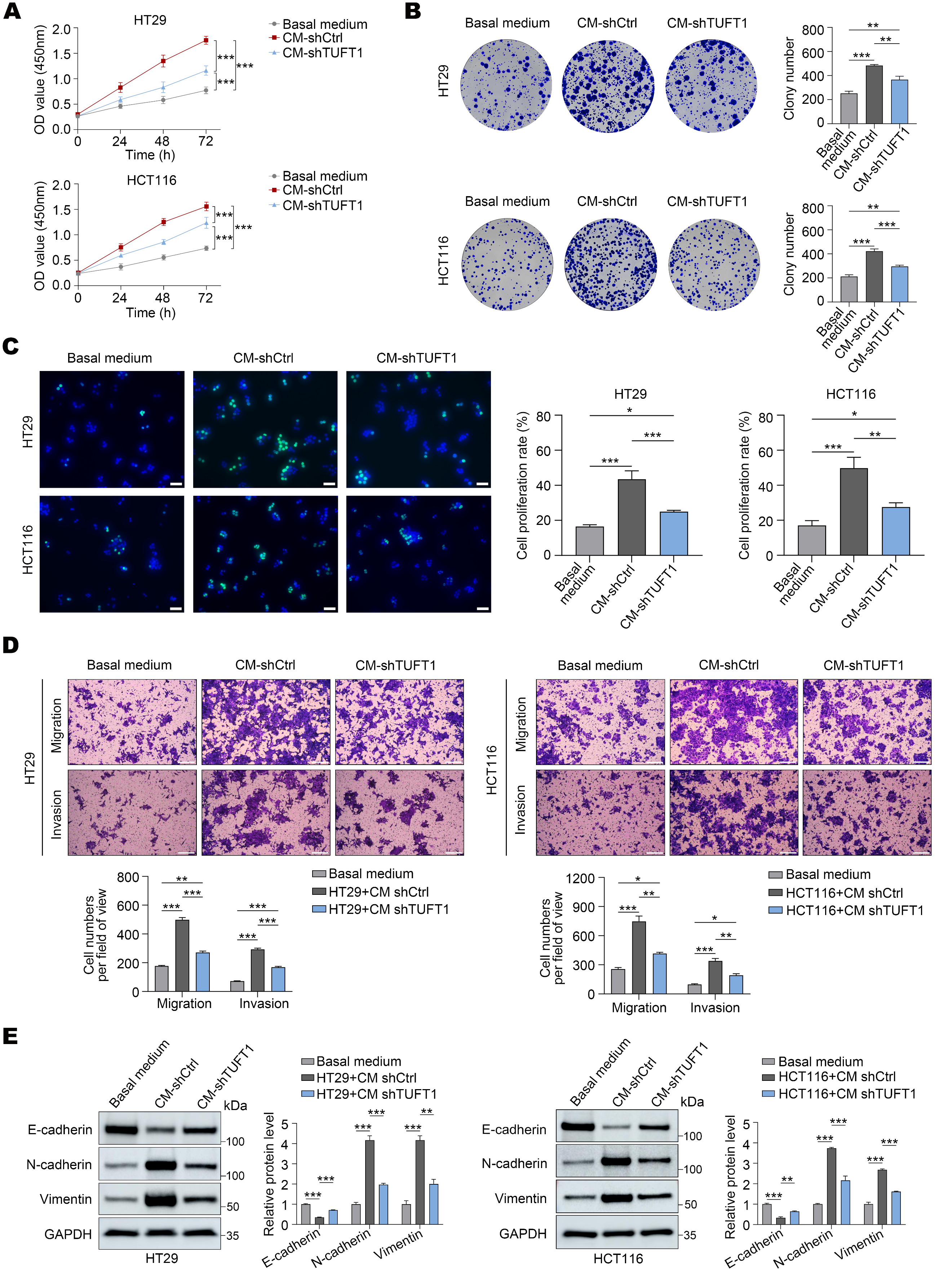


**Fig. S13 TUFT1 knockdown suppresses the tumor-promoting effect of HSCs *in vitro*. A** CCK8 assay results for HT29 and HCT116 cells incubated with conditioned medium (CM)-shCtrl or CM-shTUFT1. ***P < 0.001 by ANOVA, n = 3. **B** Colony formation assay results for HT29 and HCT116 cells incubated with CM-shCtrl or CM-shTUFT1. **P < 0.01, ***P < 0.001 by ANOVA, n = 3. **C** Edu staining showed that the rate of HT29 and HCT116 cells at the S-phase is higher in cells incubated with CM-shCtrl than in cells incubated with CM-shTUFT1. Scale bar, 50 μm. *P < 0.05, **P < 0.01, ***P < 0.001 by ANOVA, n = 3. **D** Transwell assay detected reduced effect of CM-shTUFT1 on the migration and invasion of HT29 and HCT116 cells compared to CM-shCtrl. Scale bar, 150 μm. *P < 0.05, **P < 0.01, ***P < 0.001 by ANOVA, n = 3. **E** WB revealed differential regulation of the expression of epithelial-to-mesenchymal transition (EMT) markers by HT29 and HCT116 cells incubated with CM-shCtrl or CM-shTUFT1. **P < 0.01, ***P < 0.001 by ANOVA, n = 3.


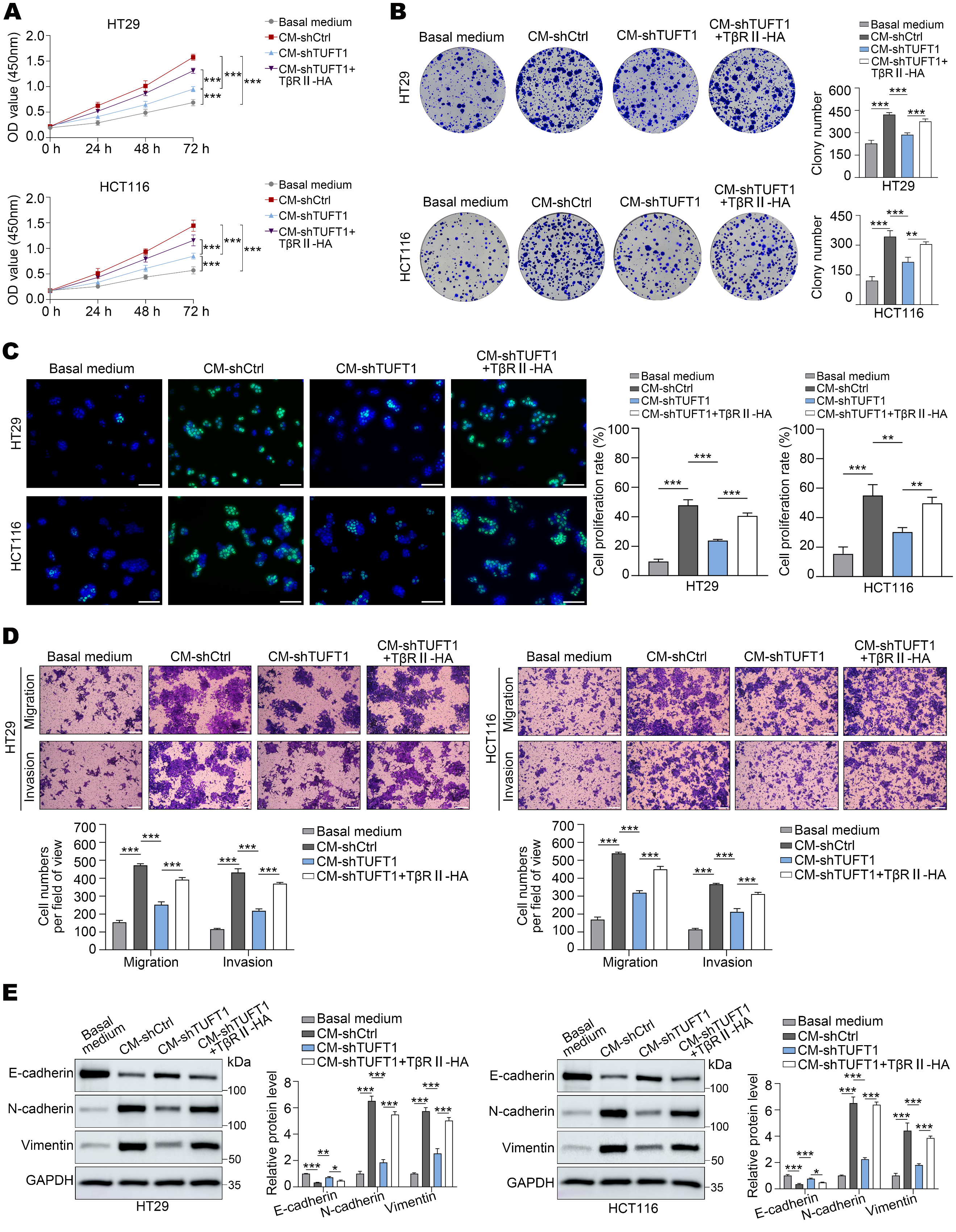


**Fig. S14 TUFT1 contributes to the tumor-promoting effect of HSCs depending on TβRII in vitro. A** HT29 and HCT116 cells were incubated with CM-shCtrl, CM-shTUFT1, or CM-shTUFT1+TβRII-HA, and cell proliferation was detected by CCK-8 assay. ***P < 0.001 by ANOVA, n = 3. **B** Colony formation ability was assessed for HT29 and HCT116 cells incubated with CM-shCtrl, CM-shTUFT1, or CM-shTUFT1+TβRII-HA. **P < 0.01, ***P < 0.001 by ANOVA, n = 3. **C** The results of Edu staining of HT29 and HCT116 cells stimulated with CM-shCtrl, CM-shTUFT1, or CM-shTUFT1+TβRII-HA. Scale bar, 100 μm. **P < 0.01, ***P < 0.001 by ANOVA, n = 3. **D** Transwell assays revealed that the effect of CM-shTUFT1 on the migration and invasion of HT29 and HCT116 cells was suppressed compared with CM-shCtrl, which was reversed by TβRII-HA overexpression in TUFT1 knockdown cells. Scale bar, 150 μm. P < 0.001 by ANOVA, n = 3. **E** WB analysis of EMT markers expressed by CRC cells incubated with CM-shCtrl, CM-shTUFT1, or CM-shTUFT1+TβRII-HA. *P < 0.05, **P < 0.01, ***P < 0.001 by ANOVA, n = 3.


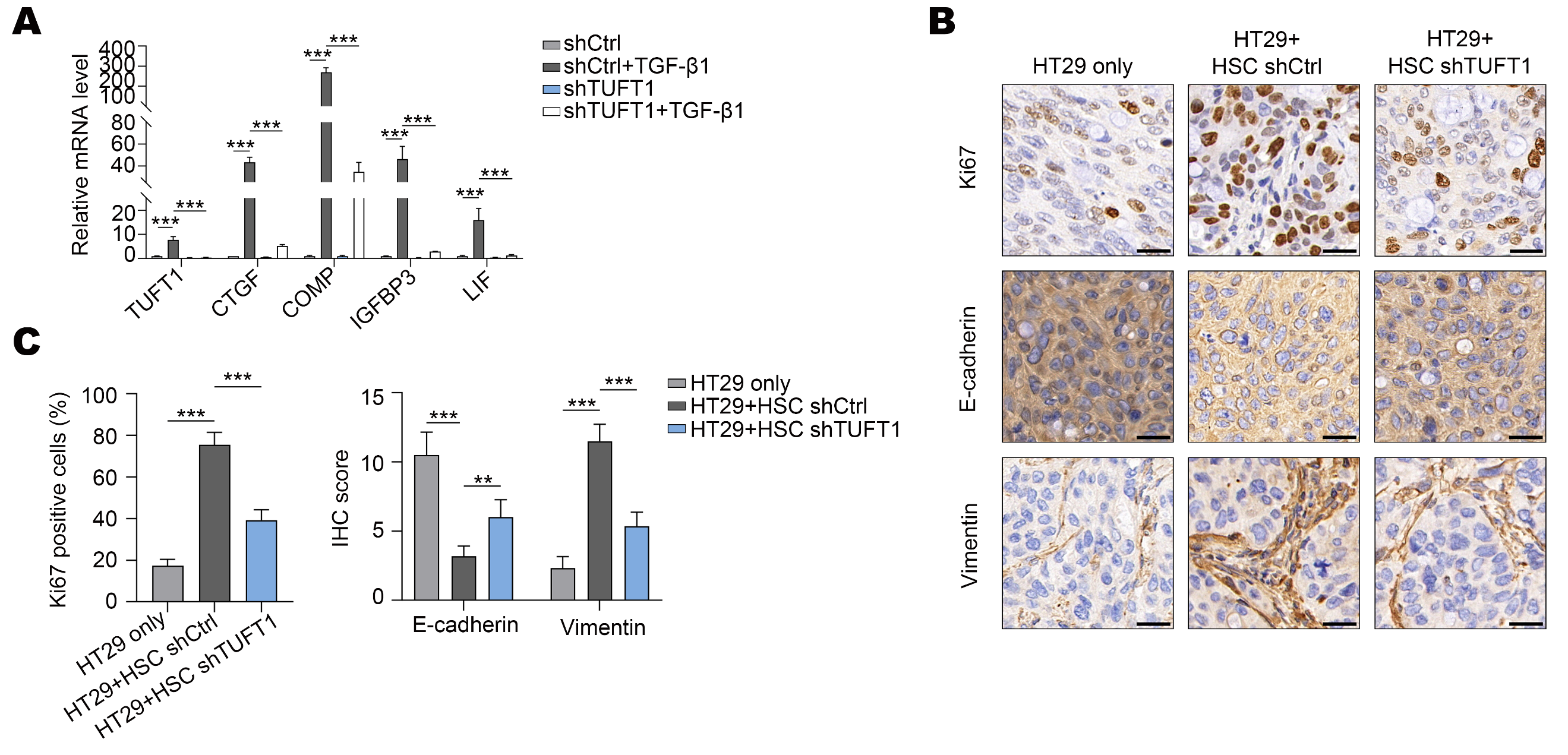


**Fig. S15 TUFT1 knockdown suppresses the tumor-promoting effect of HSCs in mice. A** qRT-PCR validated that the expression of 4 tumor-promoting factors by primary human HSCs was induced by TGFβ1 in a TUFT1-dependent manner. ***P < 0.001 by ANOVA, n = 3. **B** Pictures of IHC for Ki67, E-cadherin, and Vimentin on HT29 subcutaneous xenografts are shown. Scale bar, 25 μm. **C** Percentages of Ki67-positive cells and IHC scores of E-cadherin and Vimentin in different groups are shown. **P < 0.01, ***P < 0.001 by ANOVA, n = 6.


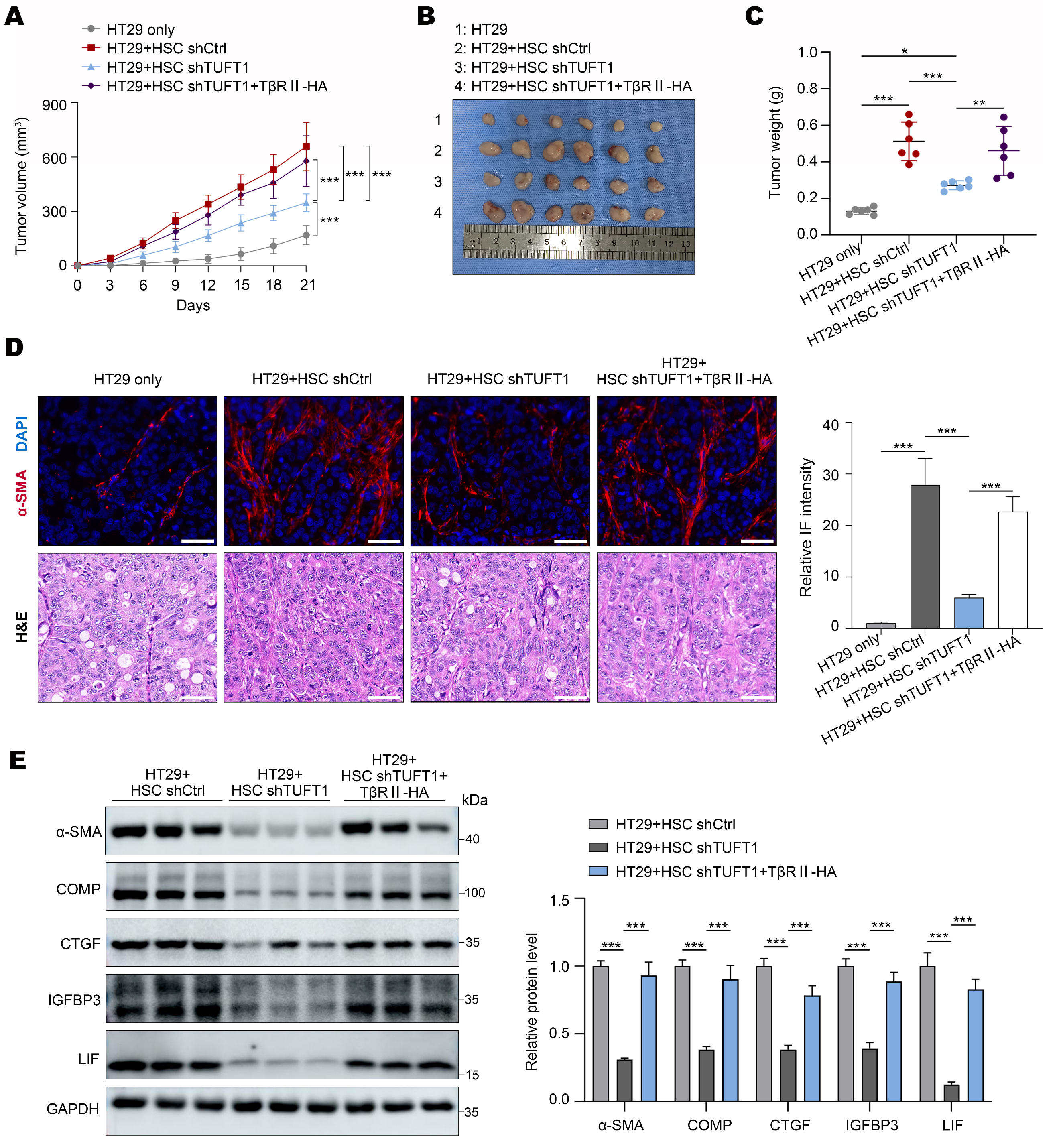


**Fig. S16 TUFT1 promotes the tumor-promoting effect of HSCs in mice via TβRII. A, B, C** Subcutaneous HT29/HSCs co-injection into nude mice revealed that knocking down TUFT1 of HSCs reduced the tumor-promoting effect of HSCs, which was partially rescued by TβRII-HA overexpression. Tumor growth curves are shown in (**A**); the pictures of HT29 tumors at the endpoint are shown in (**B**), and tumor weights are shown in (**C**). *P < 0.05, **P < 0.01, ***P < 0.001 by ANOVA, n = 6. **D** IF staining of α-SMA showed that the reduced CAF densities in tumors arising from HT29+HSC-shTUFT1 co-injections were partially restored in tumors arising from HT29+HSC-shTUFT1+TβRII-HA co-injections. Scale bar, 50 μm. ***P < 0.001 by ANOVA, n = 6. **E** The levels of HSC-derived tumor-promoting factors in tumors arising from HT29+HSC-shTUFT1 co-injections were partially restored in tumors arising from HT29+HSC-shTUFT1+TβRII-HA co-injections. ***P < 0.001 by ANOVA, n = 6.


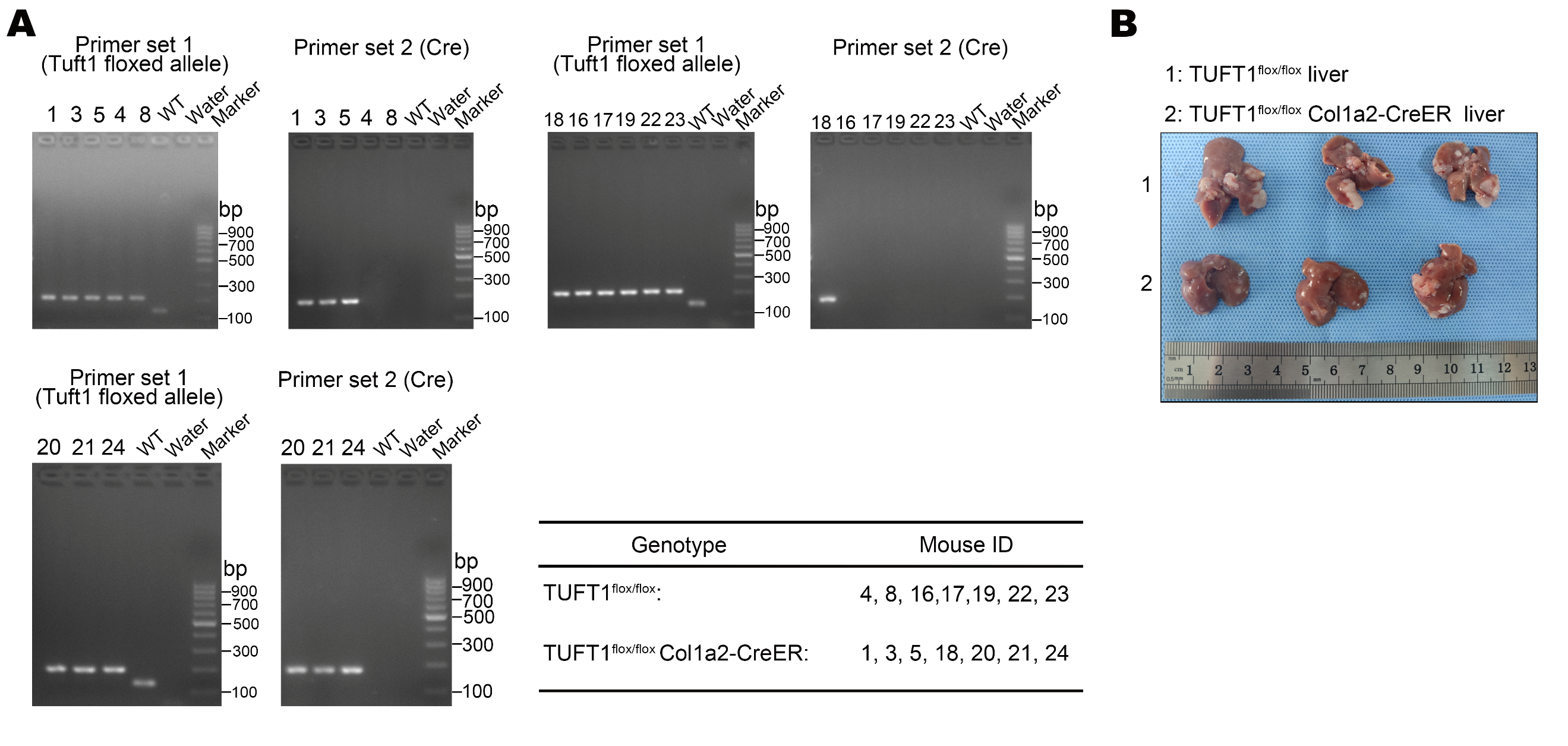


**Fig. S17 Targeting HSC TUFT1 by Cre/LoxP recombination suppresses CRCLM in mice. A** PCR genotyping results showed 7 Cre^+^ and 7 Cre^-^ mice that were homozygotes for the Tuft1 floxed alleles. The primer set 1 was used to detect the Tuft1 floxed allele; the primer set 2 was used to detect the Cre transgene. **B** Images of the murine liver after portal vein injection are shown.
