# Supplementary material for: TUFT1 stabilizes TGF-β receptor II protein and facilitates activation of hepatic stellate cells into metastasis-promoting myofibroblasts
Source: Cell Death Differ. 2026 Jan 28;33(7):1436–54. doi: 10.1038/s41418-026-01664-2 (PMC13203373; doi:10.1038/s41418-026-01664-2)
Supplement: Supplementary file 2 — Table S1 [file 41418_2026_1664_MOESM2_ESM.docx]

**Table S1.** Sequences of all shRNAs.

| Gene | Sequences |
| --- | --- |
| pLKO.1-PURO-NC | GGTTCTCCGAACGTGTCACGT |
| TUFT1 shRNA #1 | AGAAGCTCCGGGAGGATATAA |
| TUFT1 shRNA #2 | TGAGGTGGACACCTGTATAAA |
| TUFT1 shRNA #3 | CGGATGGAACACCTGATAGAA |
| TUFT1 shRNA #4 | ATGGACATGAGGAGATCATTA |
| CAV1 shRNA #1 | GCGACCCTAAACACCTCAACG |
| CAV1 shRNA #2 | GCATTTGGAAGGCCAGCTTCA |
| CAV1 shRNA #3 | GCATCCCGATGGCACTCATCT |
| CAV1 shRNA #4 | GCAATGTCCGCATCAACTTGC |
