# Supplementary material for: TUFT1 stabilizes TGF-β receptor II protein and facilitates activation of hepatic stellate cells into metastasis-promoting myofibroblasts
Source: Cell Death Differ. 2026 Jan 28;33(7):1436–54. doi: 10.1038/s41418-026-01664-2 (PMC13203373; doi:10.1038/s41418-026-01664-2)
Supplement: Supplementary file 3 — Table S2 [file 41418_2026_1664_MOESM3_ESM.docx]

**Table S2.** Primer sequences for qRT-PCR.

| Gene | Forward Sequence | Reverse Sequence |
| --- | --- | --- |
| TβRⅠ | GTTCCGTGAGGCAGAGATTTAT | ACCAGAGCTGAGTCCAAGTA |
| TβRⅡ | GTCGCTTTGCTGAGGTCTATAA | CTCTGTCTTCCAAGAGGCATAC |
| TUFT1 | AGAAGCTCCGGGAGGATATAA | GCTGTGGGACTCTGACTAAAG |
| CAV1 | GACCCTAAACACCTCAACGATGA | CCAGATGTGCAGGAAAGAGAGAA |
| CTGF | GCTGACCTGGAAGAGAACATTA | TGCAGCCAGAAAGCTCAA |
| ACTA2 | GATGGTGGGAATGGGACAAA | GCCATGTTCTATCGGGTACTTC |
| COMP | GATCACGTTCCTGAAAAACACG | GCTCTCCGTCTGGATGCAG |
| IGFBP3 | AGAGCACAGATACCCAGAACT | GGTGATTCAGTGTGTCTTCCATT |
| LIF | CCAACGTGACGGACTTCCC | TACACGACTATGCGGTACAGC |
| CXCL9 | CCAGTAGTGAGAAAGGGTCGC | AGGGCTTGGGGCAAATTGTT |
| CXCL10 | GTGGCATTCAAGGAGTACCTC | TGATGGCCTTCGATTCTGGATT |
| GAPDH | ACATCGCTCAGACACCATG | TGTAGTTGAGGTCAATGAAGGG |
