# Supplementary material for: TUFT1 stabilizes TGF-β receptor II protein and facilitates activation of hepatic stellate cells into metastasis-promoting myofibroblasts
Source: Cell Death Differ. 2026 Jan 28;33(7):1436–54. doi: 10.1038/s41418-026-01664-2 (PMC13203373; doi:10.1038/s41418-026-01664-2)
Supplement: Supplementary file 4 — Table S3 [file 41418_2026_1664_MOESM4_ESM.docx]

**Table S3.** Antibodies information.

| Antibody | Application and dilution ratio | Source | Cat No. |
| --- | --- | --- | --- |
| Rabbit monoclonal anti-TUFT1 | WB (1:2000) | Abcam | ab184949 |
| Rabbit polyclonal anti-TUFT1 | IF (1:50); IHC (1:200) | Proteintech | 23385-1-AP |
| Mouse monoclonal anti-TUFT1 | IP (1:100) | Santa Cruz | sc-365632 |
| Rabbit monoclonal anti-TGFβRⅠ | WB (1:1000) | Abcam | ab235578 |
| Rabbit monoclonal anti-TGFβRⅡ | WB (1:2500) | Abcam | ab184948 |
| Mouse monoclonal anti-TGFβRⅡ | IP (1:100) | Santa Cruz | sc-17799 |
| Mouse monoclonal anti-TGFβRⅡ | IHC (1:500) | Proteintech | 66636-1-Ig |
| Rabbit monoclonal anti-CTGF | WB (1:1000) | Abcam | ab209780 |
| Rabbit monoclonal anti-CTGF | WB (1:1000) | Proteintech | 82976-1-RR |
| Rabbit monoclonal anti-Fibronectin | WB (1:1000) | Abcam | ab45688 |
| Rabbit monoclonal anti-α-SMA | WB (1:1000) | Abcam | ab124964 |
| Mouse monoclonal anti-α-SMA | IF (1:1000) | BOSTER | BM0002 |
| Goat anti-Type I Collagen | WB (1:2000) | Southern Biotech | 1310-01 |
| Rabbit monoclonal anti-SMAD2/3 | WB (1:1000) | CST | 8685 |
| Rabbit monoclonal anti-Phospho-SMAD2 (Ser465/467) | WB (1:1000) | CST | 3108 |
| Rabbit monoclonal anti-Phospho-SMAD3 (Ser423/425) | WB (1:1000) | CST | 9520 |
| Mouse monoclonal anti-Smad2/3 | IF (1:50) | Santa Cruz | sc-133098 |
| Mouse monoclonal anti-GAPDH | WB (1:5000) | Proteintech | 60004-1-Ig |
| Mouse monoclonal anti-Ubiquitin | WB (1:100) | Santa Cruz | sc-8017 |
| Mouse monoclonal anti-LAMP1 | IF (1:50) | Santa Cruz | sc-20011 |
| Mouse monoclonal anti-Caveolin-1 | WB (1:2500); IF (1:300) | Proteintech | 66067-1-Ig |
| Rabbit polyclonal anti-E-cadherin | WB (1:5000); IHC (1:5000) | Proteintech | 20874-1-AP |
| Rabbit polyclonal anti-N-cadherin | WB (1:5000) | Proteintech | 22018-1-AP |
| Rabbit polyclonal anti-Vimentin | WB (1:4000); IHC (1:2500) | Proteintech | 10366-1-AP |
| Rabbit polyclonal anti-Ki67 | IHC (1:5000) | Proteintech | 27309-1-AP |
| Rabbit polyclonal anti-IGFBP3 | WB (1:1000) | Immunoway | YT2286 |
| Rabbit polyclonal anti-LIF | WB (1:300) | Proteintech | 26757-1-AP |
| Rabbit monoclonal anti-COMP | WB (1:1000) | ABclonal | A22260 |
| Mouse monoclonal anti-EEA1 | IF (1:1500) | Proteintech | 68065-1-Ig |
| Rabbit monoclonal anti-HA | IP (1:50); WB (1:1000); IF (1:1000) | CST | 3724 |
| Mouse monoclonal anti-HA | IP (1:200); WB (1:5000); IF (1:1000) | Immunoway | YM3003 |
| Rabbit monoclonal anti-Flag | WB (1:1000) | CST | 14793 |
| Mouse monoclonal anti-Flag | IP (1:200); WB (1:1000) | Sigma | F1804 |
| Goat Anti-Rabbit IgG, FITC Conjugated | IF (1:200) | Zhuangzhi-BIO | EK023 |
| Goat Anti-Mouse IgG, FITC Conjugated | IF (1:200) | Zhuangzhi-BIO | EK013 |
| Goat Anti-Mouse IgG, Cy3 Conjugated | IF (1:200) | Zhuangzhi-BIO | EK012 |
| Goat Anti-Rabbit IgG, Cy3 Conjugated | IF (1:200) | Zhuangzhi-BIO | EK022 |
