# Supplementary material for: TUFT1 stabilizes TGF-β receptor II protein and facilitates activation of hepatic stellate cells into metastasis-promoting myofibroblasts
Source: Cell Death Differ. 2026 Jan 28;33(7):1436–54. doi: 10.1038/s41418-026-01664-2 (PMC13203373; doi:10.1038/s41418-026-01664-2)
Supplement: Supplementary file 5 — Table S4 [file 41418_2026_1664_MOESM5_ESM.docx]

**Table S4.** Primer sequences for ChIP-qPCR.

| Gene | Forward Sequence | Reverse Sequence |
| --- | --- | --- |
| TUFT1 | GGCAGCGTCAGTAAAAGAGC | AAGCCACCTGTAGGCTTTGT |
