# Supplementary material for: TUFT1 stabilizes TGF-β receptor II protein and facilitates activation of hepatic stellate cells into metastasis-promoting myofibroblasts
Source: Cell Death Differ. 2026 Jan 28;33(7):1436–54. doi: 10.1038/s41418-026-01664-2 (PMC13203373; doi:10.1038/s41418-026-01664-2)

Fig. 1C

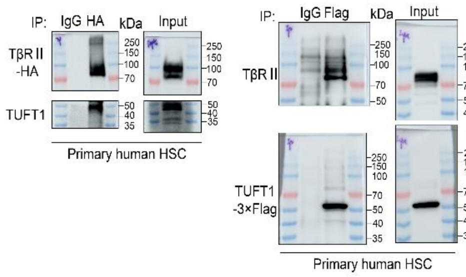

Fig. 1D

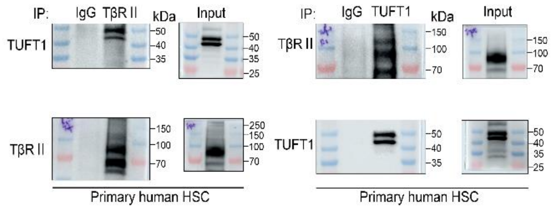

Fig. 2A

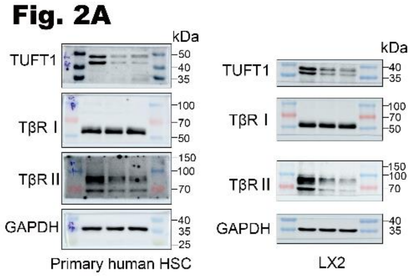

Fig. 2C

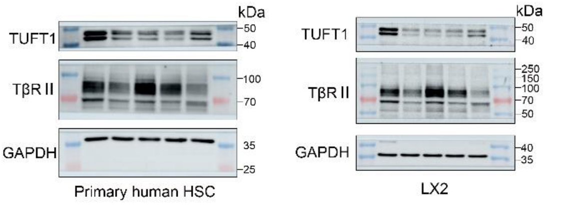

Fig. 2D

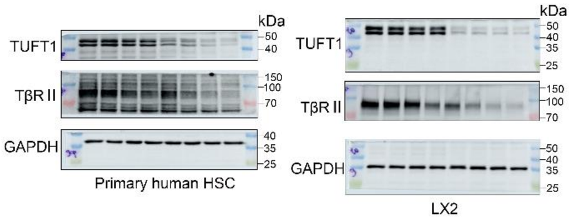

Fig. 2F

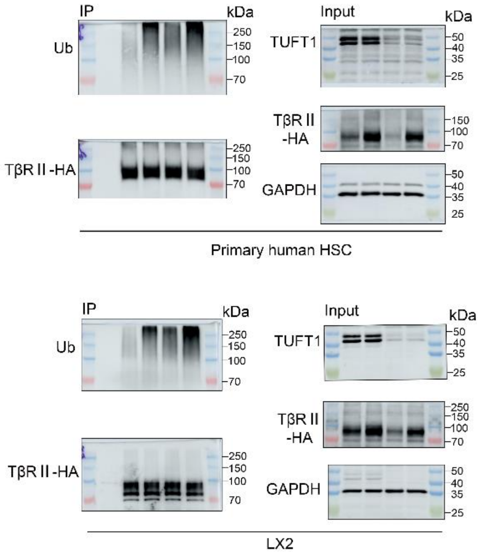

Fig. 2G

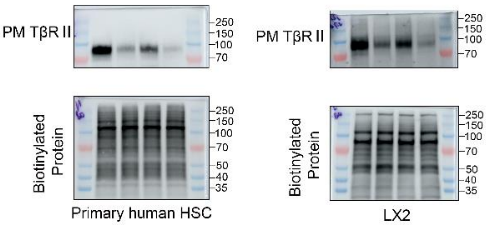

Fig. 3B

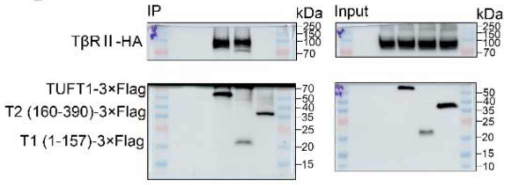

Fig. 3D

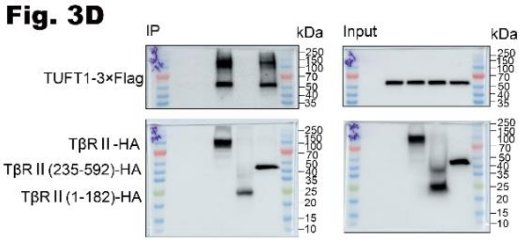

Fig. 3E

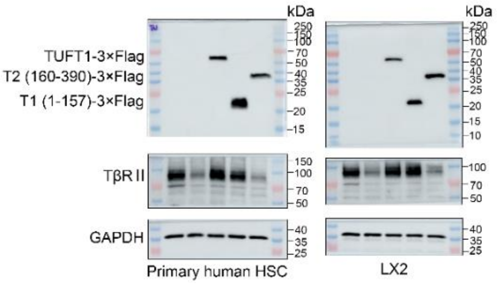

Fig. 3F

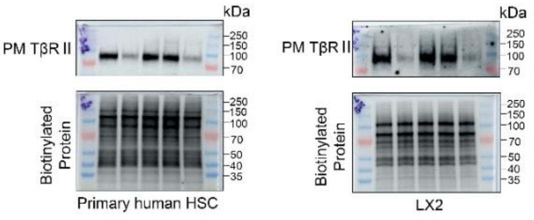

Fig. 4A

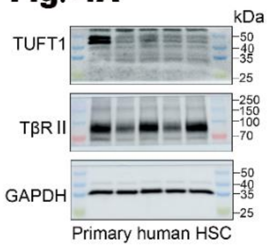

Fig. 4B

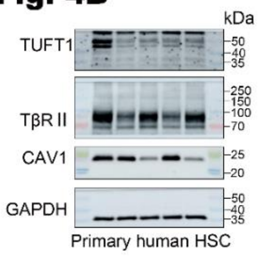

Fig. 4D

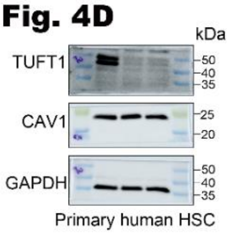

Fig. 4E

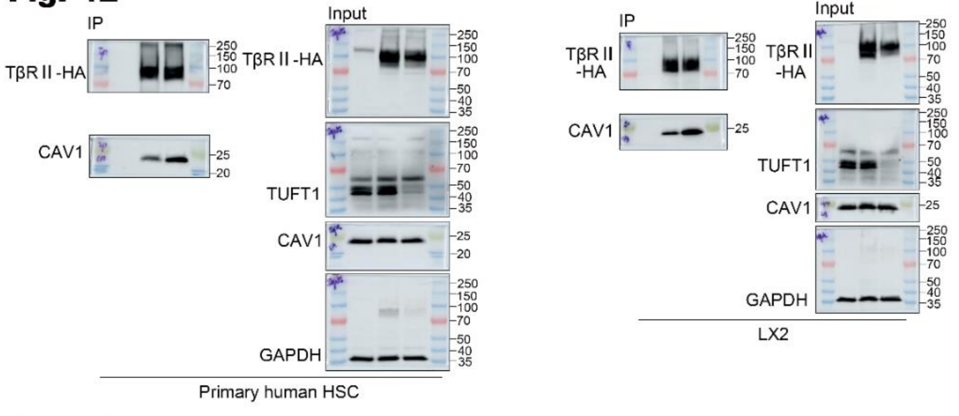

Fig. 4F

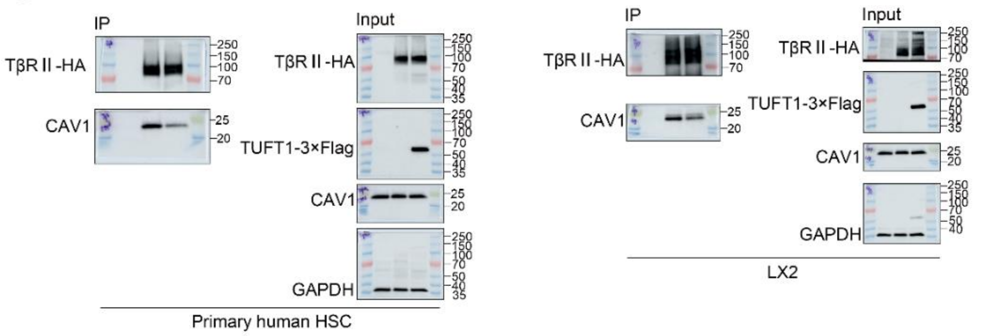

Fig. 6A

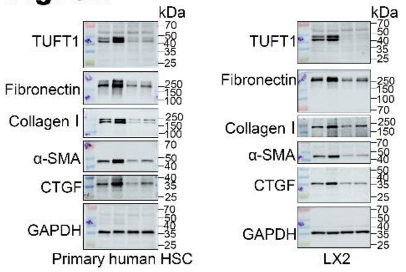

Fig. 6C

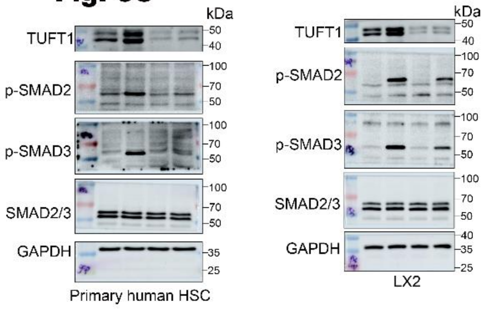

Fig. 6E

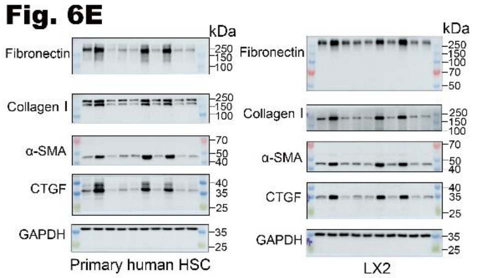

Fig. 6F

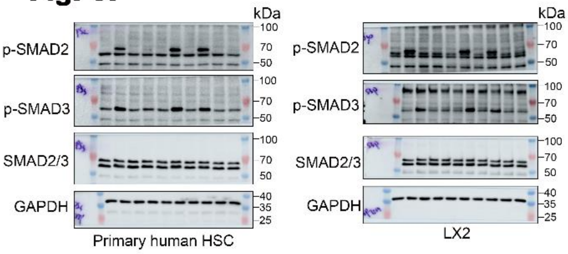

Fig. 7B

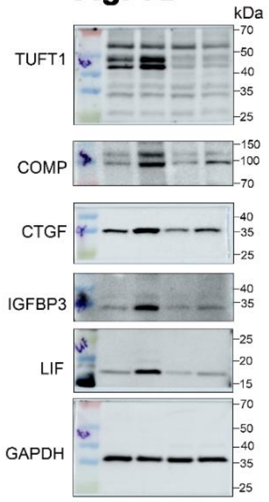

Fig. 7G

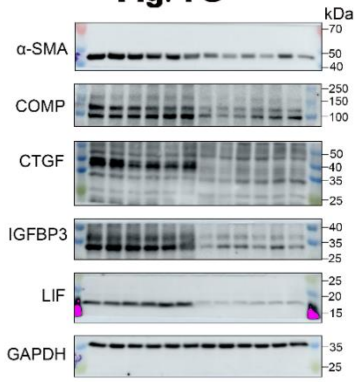

Fig. 8E

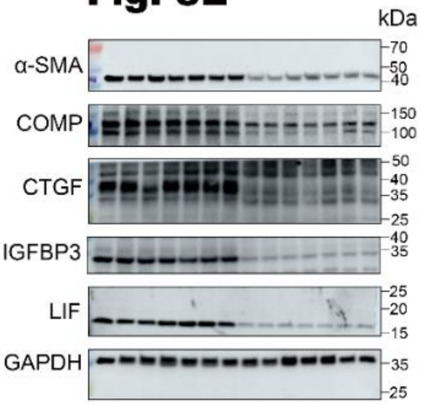

Fig. S1A

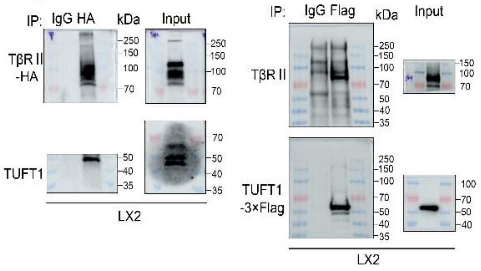

Fig. S1B

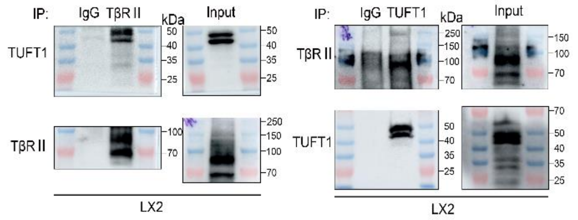

Fig. S3B

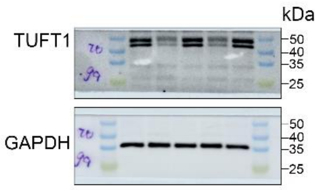

Fig. S4C

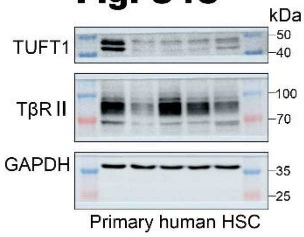

Fig. S4D

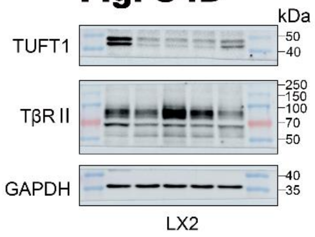

Fig. S4E

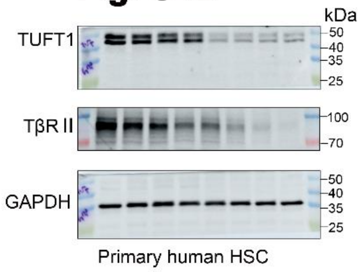

Fig. S4F

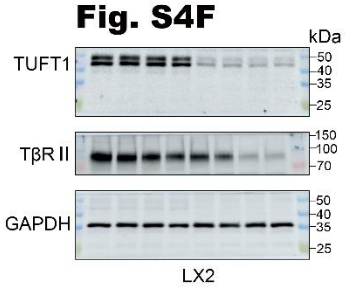

Fig. S5B

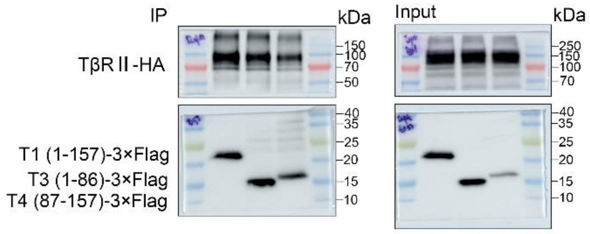

Fig. S6B

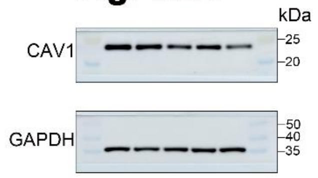

Fig. S7A

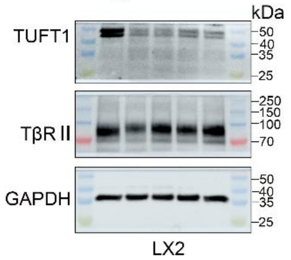

Fig. S7B

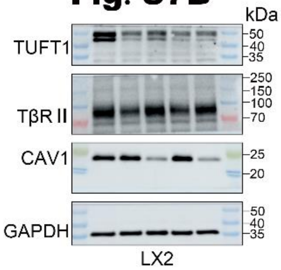

Fig. S7D

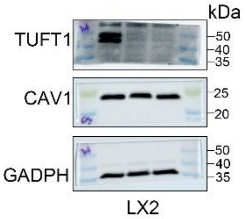

Fig. S8A

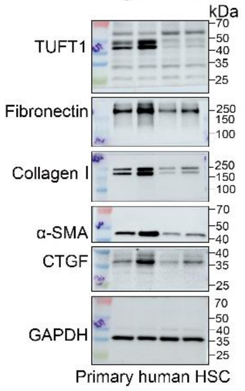

Fig. S8B

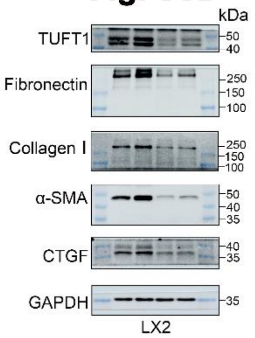

Fig. S8C

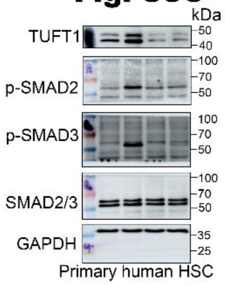

Fig. S8D

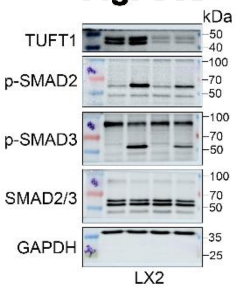

Fig. S9A

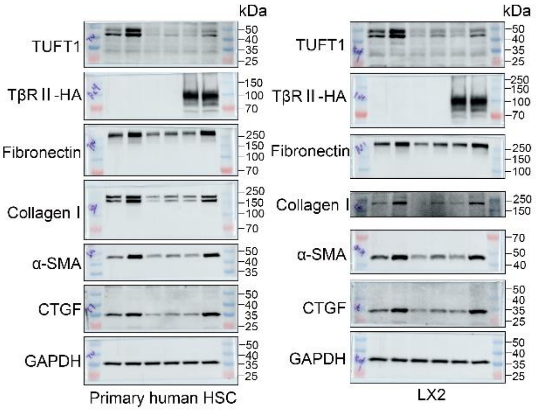

Fig. S9B

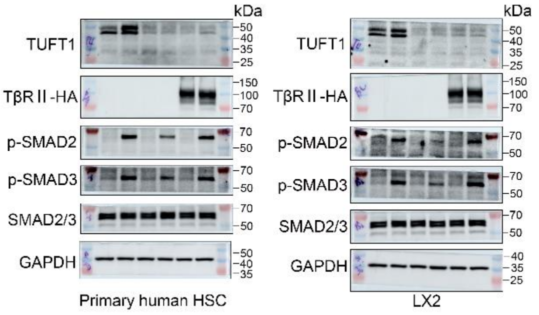

Fig. S12A

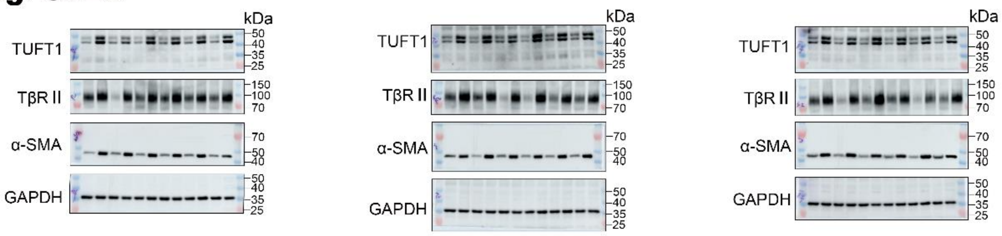

Fig. S13E

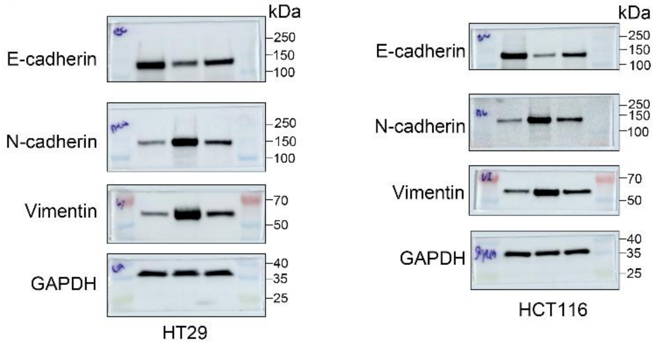

Fig. S14E

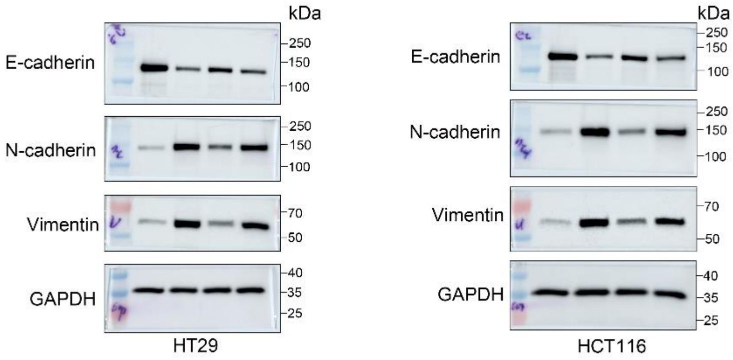

Fig. S16E

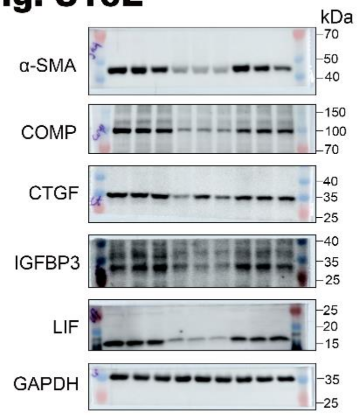

Supplement: Supplementary file 6 — Uncropped original western blots [file 41418_2026_1664_MOESM6_ESM.pdf]
