# Supplementary material for: TUFT1 stabilizes TGF-β receptor II protein and facilitates activation of hepatic stellate cells into metastasis-promoting myofibroblasts
Source: Cell Death Differ. Author manuscript; Available in PMC 2026 May 27. (PMC13203373; doi:10.1038/s41418-026-01664-2)
Supplement: Supplementary file 1 [file NIHMS2172331-supplement-Suppl__Table_1.pdf]

Table S1. Sequences of all shRNAs.

Gene	Sequences
pLKO.1-PURO-NC	GGTTCTCCGAACGTGTCACGT
TUFT1 shRNA #1	AGAAGCTCCGGGAGGATATAA
TUFT1 shRNA #2	TGAGGTGGACACCTGTATAAA
TUFT1 shRNA #3	CGGATGGAACACCTGATAGAA
TUFT1 shRNA #4	ATGGACATGAGGAGATCATTA
CAV1 shRNA #1	GCGACCCTAAACACCTCAACG
CAV1 shRNA #2	GCATTTGGAAGGCCAGCTTCA
CAV1 shRNA #3	GCATCCCGATGGCACTCATCT
CAV1 shRNA #4	GCAATGTCCGCATCAACTTGC
