# Supplementary material for: TUFT1 stabilizes TGF-β receptor II protein and facilitates activation of hepatic stellate cells into metastasis-promoting myofibroblasts
Source: Cell Death Differ. Author manuscript; Available in PMC 2026 May 27. (PMC13203373; doi:10.1038/s41418-026-01664-2)
Supplement: Supplementary file 2 [file NIHMS2172331-supplement-Suppl__Table_2.pdf]

Table S2. Primer sequences for qRT-PCR.

Gene	Forward Sequence	Reverse Sequence
TβRI	GTTCCGTGAGGCAGAGATTTAT	ACCAGAGCTGAGTCCAAGTA
TβRII	GTCGCTTTGCTGAGGTCTATAA	CTCTGTCTTCCAAGAGGCATAC
TUFT1	AGAAGCTCCGGGAGGATATAA	GCTGTGGGACTCTGACTAAAG
CAV1	GACCCTAAACACCTCAACGATGA	CCAGATGTGCAGGAAAGAGAGAA
CTGF	GCTGACCTGGAAGAGAACATTA	TGCAGCCAGAAAGCTCAA
ACTA2	GATGGTGGGAATGGGACAAA	GCCATGTTCTATCGGGTACTTC
COMP	GATCACGTTCCTGAAAAACACG	GCTCTCCGTCTGGATGCAG
IGFBP3	AGAGCACAGATACCCAGAACT	GGTGATTCAGTGTGTCTTCCATT
LIF	CCAACGTGACGGACTTCCC	TACACGACTATGCGGTACAGC
CXCL9	CCAGTAGTGAGAAAGGGTCGC	AGGGCTTGGGGCAAATTGTT
CXCL10	GTGGCATTCAAGGAGTACCTC	TGATGGCCTTCGATTCTGGATT
GAPDH	ACATCGCTCAGACACCATG	TGTAGTTGAGGTCAATGAAGGG
