# Supplementary material for: TUFT1 stabilizes TGF-β receptor II protein and facilitates activation of hepatic stellate cells into metastasis-promoting myofibroblasts
Source: Cell Death Differ. Author manuscript; Available in PMC 2026 May 27. (PMC13203373; doi:10.1038/s41418-026-01664-2)
Supplement: Supplementary file 3 [file NIHMS2172331-supplement-Suppl__Table_3.pdf]

Table S3. Antibodies information.

Antibody	Application and dilution ratio	Source	Cat No.
Rabbit monoclonal anti-TUFT1	WB (1:2000)	Abcam	ab184949
Rabbit polyclonal anti-TUFT1	IF (1:50); IHC (1:200)	Proteintech	23385-1-AP
Mouse monoclonal anti-TUFT1	IP (1:100)	Santa Cruz	sc-365632
Rabbit monoclonal anti-TGFβRI	WB (1:1000)	Abcam	ab235578
Rabbit monoclonal anti-TGFβRII	WB (1:2500)	Abcam	ab184948
Mouse monoclonal anti-TGFβRII	IP (1:100)	Santa Cruz	sc-17799
Mouse monoclonal anti-TGFβRII	IHC (1:500)	Proteintech	66636-1-Ig
Rabbit monoclonal anti-CTGF	WB (1:1000)	Abcam	ab209780
Rabbit monoclonal anti-CTGF	WB (1:1000)	Proteintech	82976-1-RR
Rabbit monoclonal anti-Fibronectin	WB (1:1000)	Abcam	ab45688
Rabbit monoclonal anti-α-SMA	WB (1:1000)	Abcam	ab124964
Mouse monoclonal anti-α-SMA	IF (1:1000)	BOSTER	BM0002
Goat anti-Type I Collagen	WB (1:2000)	Southern Biotech	1310-01
Rabbit monoclonal anti-SMAD2/3	WB (1:1000)	CST	8685
Rabbit monoclonal anti-Phospho-SMAD2 (Ser465/467)	WB (1:1000)	CST	3108
Rabbit monoclonal anti-Phospho-SMAD3 (Ser423/425)	WB (1:1000)	CST	9520
Mouse monoclonal anti-Smad2/3	IF (1:50)	Santa Cruz	sc-133098
Mouse monoclonal anti-GAPDH	WB (1:5000)	Proteintech	60004-1-Ig
Mouse monoclonal anti-Ubiquitin	WB (1:100)	Santa Cruz	sc-8017
Mouse monoclonal anti-LAMP1	IF (1:50)	Santa Cruz	sc-20011
Mouse monoclonal anti-Caveolin-1	WB (1:2500); IF (1:300)	Proteintech	66067-1-Ig
Rabbit polyclonal anti-E-cadherin	WB (1:5000); IHC (1:5000)	Proteintech	20874-1-AP
Rabbit polyclonal anti-N-cadherin	WB (1:5000)	Proteintech	22018-1-AP
Rabbit polyclonal anti-Vimentin	WB (1:4000); IHC (1:2500)	Proteintech	10366-1-AP
Rabbit polyclonal anti-Ki67	IHC (1:5000)	Proteintech	27309-1-AP
Rabbit polyclonal anti-IGFBP3	WB (1:1000)	Immunoway	YT2286
Rabbit polyclonal anti-LIF	WB (1:300)	Proteintech	26757-1-AP
Rabbit monoclonal anti-COMP	WB (1:1000)	ABclonal	A22260
Mouse monoclonal anti-EEA1	IF (1:1500)	Proteintech	68065-1-Ig
Rabbit monoclonal anti-HA	IP (1:50); WB	CST	3724

	(1:1000); IF (1:1000)		
Mouse monoclonal anti-HA	IP (1:200); WB (1:5000); IF (1:1000)	Immunoway	YM3003
Rabbit monoclonal anti-Flag	WB (1:1000)	CST	14793
Mouse monoclonal anti-Flag	IP (1:200); WB (1:1000)	Sigma	F1804
Goat Anti-Rabbit IgG, FITC Conjugated	IF (1:200)	Zhuangzhi-BIO	EK023
Goat Anti-Mouse IgG, FITC Conjugated	IF (1:200)	Zhuangzhi-BIO	EK013
Goat Anti-Mouse IgG, Cy3 Conjugated	IF (1:200)	Zhuangzhi-BIO	EK012
Goat Anti-Rabbit IgG, Cy3 Conjugated	IF (1:200)	Zhuangzhi-BIO	EK022
