# Supplementary material for: TUFT1 stabilizes TGF-β receptor II protein and facilitates activation of hepatic stellate cells into metastasis-promoting myofibroblasts
Source: Cell Death Differ. Author manuscript; Available in PMC 2026 May 27. (PMC13203373; doi:10.1038/s41418-026-01664-2)
Supplement: Supplementary file 4 [file NIHMS2172331-supplement-Suppl__Table_4.pdf]

Table S4. Primer sequences for ChIP-qPCR.

Gene	Forward Sequence	Reverse Sequence
TUFT1	GGCAGCGTCAGTAAAAGAGC	AAGCCACCTGTAGGCTTTGT
