# Supplementary material for: TUFT1 stabilizes TGF-β receptor II protein and facilitates activation of hepatic stellate cells into metastasis-promoting myofibroblasts
Source: Cell Death Differ. Author manuscript; Available in PMC 2026 May 27. (PMC13203373; doi:10.1038/s41418-026-01664-2)
Supplement: Supplementary file 5 [file NIHMS2172331-supplement-Supplementary_information.pdf]

Supplementary Figures and Legends

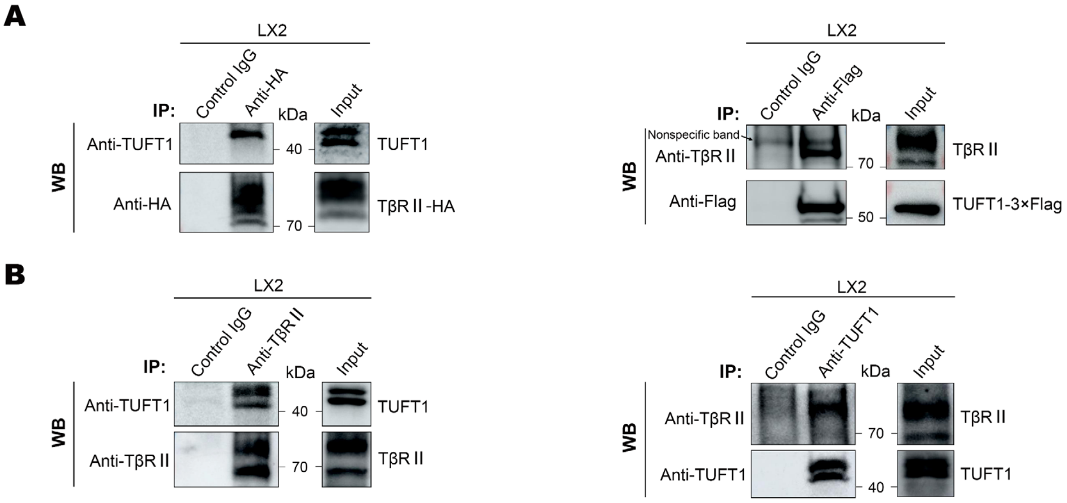

Fig. S1 TUFT1 interacts with TGF-β receptor II (TβRII) in LX2 cells. A LX2 cells overexpressing TβRII-HA (left) or overexpressing TUFT1-3×Flag (right) were collected for co-IP with anti-HA or anti-Flag antibody; co-precipitated TUFT1 or TβRII was detected by Western blot (WB) analysis. Data represent 3 repeats with similar results. **B** Co-immunoprecipitation (co-IP) confirmed the interaction between endogenous TβRII and TUFT1 in LX2 cells using anti-TβRII antibody (left) or anti-TUFT1 antibody (right). Data represent 3 repeats with similar results.

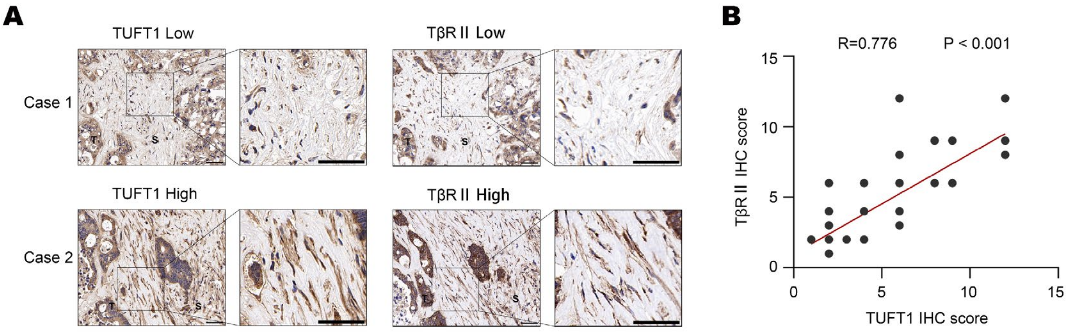

Fig. S2 TUFT1 and TβRII expression are positively correlated in the stroma of CRCLM of the patients. A Immunohistochemistry (IHC) was performed on patient

CRCLM tissue, and representative IHC images for TUFT1 and TβRII are shown. Scale bar, 50 μm. **B** Patient IHC data were subjected to Spearman correlation analysis, which revealed that TUFT1 and TβRII levels were positively correlated in 40 CRCLM samples. R=0.776, P < 0.001.

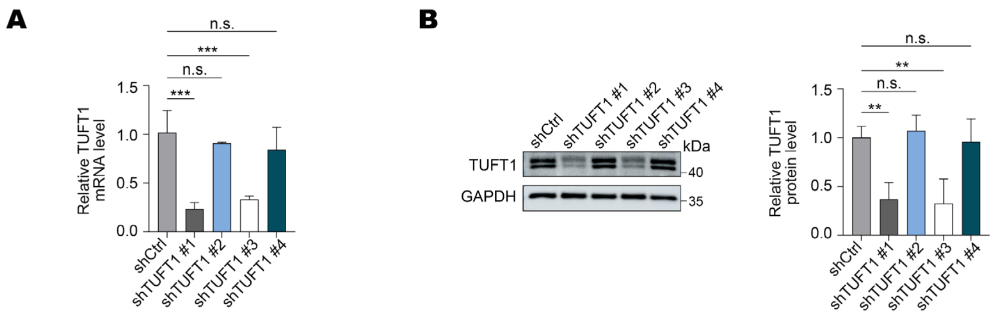

Fig. S3 Validation of the knockdown effect of TUFT1 shRNAs. A qRT-PCR for TUFT1 was performed on cells transduced with each of 4 different TUFT1 shRNA lentiviruses. Not significant (n.s., P > 0.05), ***P < 0.001 by ANOVA, n = 3. **B** Western blot (WB) validation for TUFT1 knockdown. Not significant (n.s., P > 0.05), **P < 0.01 by ANOVA, n = 3.

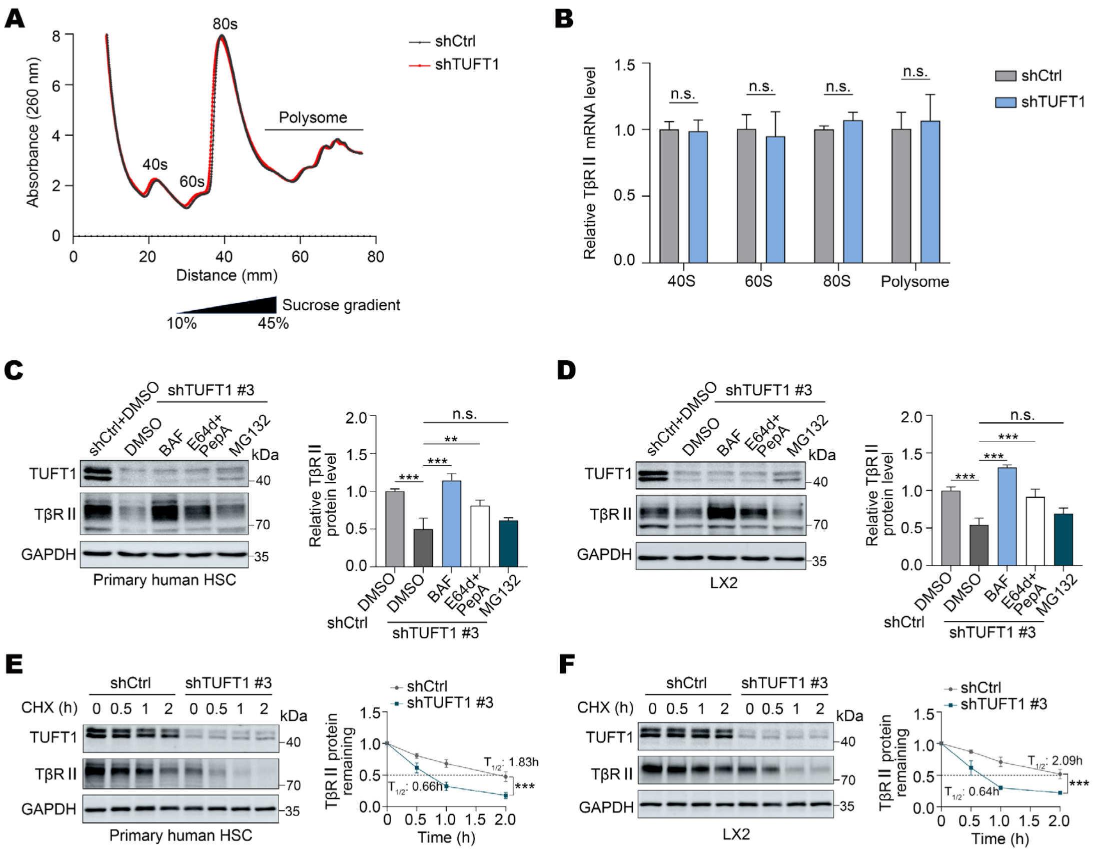

Fig. S4 TUFT1 knockdown promotes lysosome-mediated degradation of TβRII. A Polysome profiling by sucrose density gradient showed that the polysome distribution profile in TUFT1 knockdown LX2 cells remained unchanged compared to control cells. **B** Quantification of TβRII mRNA level in each fraction by qRT-PCR demonstrated that TUFT1 knockdown did not alter TβRII mRNA translation. n.s., not significant; $P > 0.05$ by t-test, $n = 3$. **C** Primary human HSCs and **D** LX2 cells transduced with TUFT1 shRNA #3 lentiviruses were incubated with bafilomycin A1 [BAF, 10 nM] or E64d [10 μg/mL] + pepstatin A [PepA, 10 μg/mL], or proteasome inhibitor (MG132, 25 μM) for 8 hours were subjected to WB for TβRII. The TβRII protein levels affected by TUFT1 knockdown were restored by the lysosomal inhibitors, but not by the proteasomal inhibitor. Not significant (n.s., $P > 0.05$), $^{**}P< 0.01$, $^{***}P < 0.001$ by ANOVA, $n = 3$. **E** TUFT1 shRNA #3 lentiviruses were used to knock down TUFT1 of primary human

HSCs and **F** LX2 cells, and the cells were subjected to TβRII degradation analysis by WB. TβRII degraded much faster in TUFT1 knockdown cells compared to control cells. ***P < 0.001 by ANOVA, n = 3.

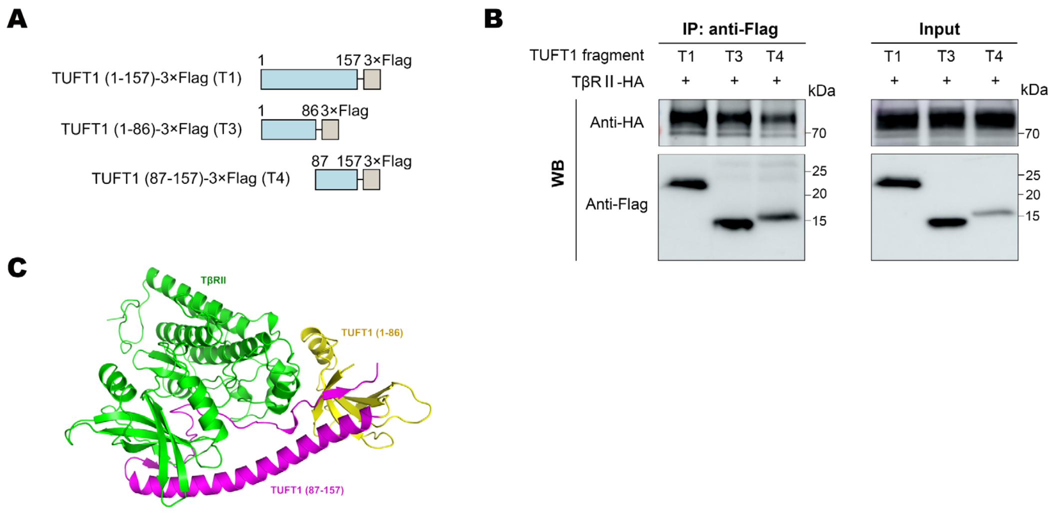

Fig. S5 TβRII interacts with N-terminal a.a. 1-86 and a.a. 87-157 of TUFT1. A Schematic diagram for TUFT1 (a.a. 1-157)-3×Flag, TUFT1 (a.a. 1-86)-3×Flag, and TUFT1 (a.a. 87-157)-3×Flag truncation mutants. **B** TβRII-HA was co-transfected with either TUFT1 truncation mutant shown in **A** into 293T cells for co-IP with anti-Flag antibody. Both a.a. 1-86 and a.a. 87-157 fragments of TUFT1 can interact with TβRII. **C** The structural model of the TUFT1 (a.a. 1-157)/TβRII complex was predicted with Boltz-2 (based on AlphaFold3) using default parameters and visualized using PyMOL. In the representation, TUFT1 (a.a. 1-86), TUFT1 (a.a. 87-157), and TβRII are colored in yellow, magenta, and green, respectively.

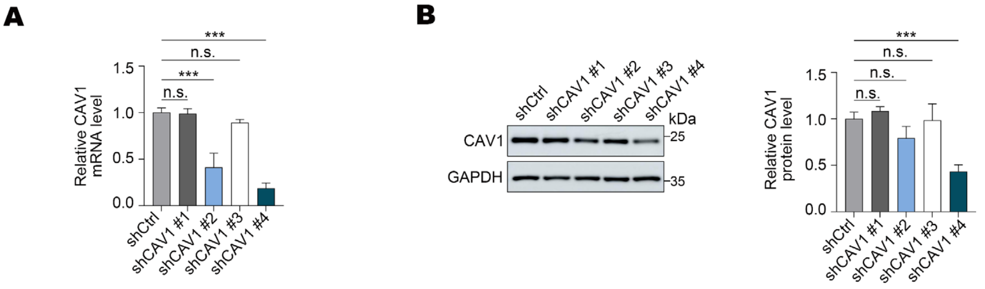

Fig. S6 Validation of the knockdown effect of CAV1 shRNAs. A qRT-PCR validation of CAV1 knockdown by different shRNA lentiviruses. Not significant (n.s., p > 0.05), ***P < 0.001 by ANOVA, n = 3. **B** WB validation of CAV1 knockdown by different shRNA lentiviruses. Not significant (n.s., P > 0.05), ***P < 0.001 by ANOVA, n = 3.

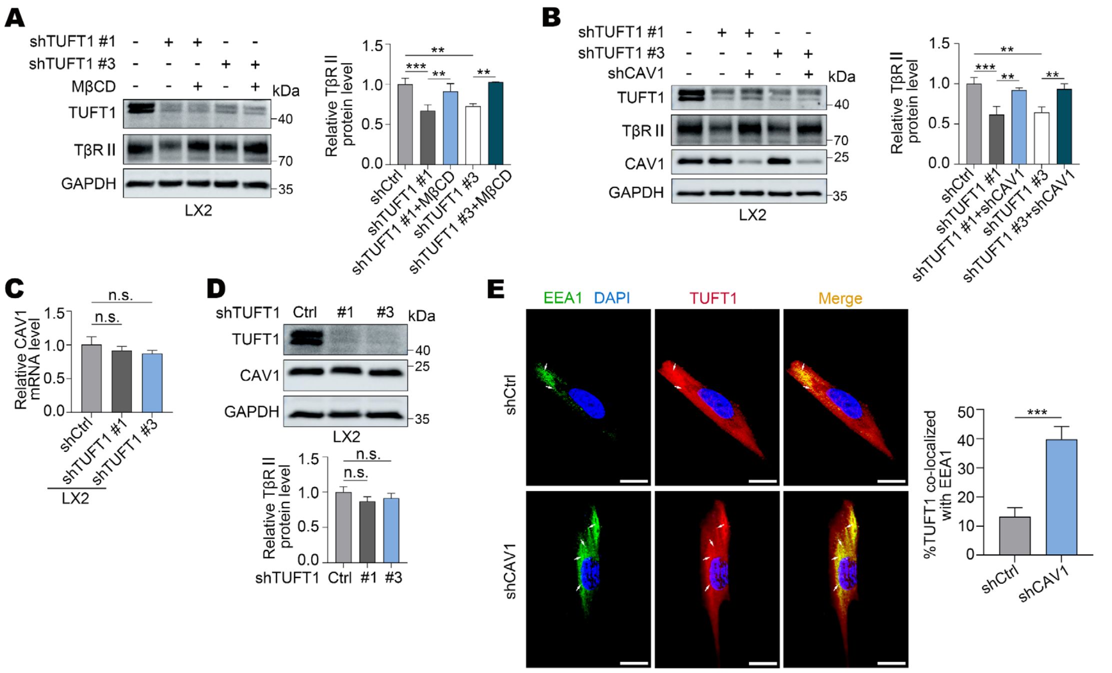

Fig. S7 Targeting TUFT1 induces lipid rafts/caveolae-mediated degradation of TβRII. A Control and TUFT1 knockdown LX2 cells were incubated with Methyl-β-cyclodextrin (MβCD, 1.5 mM) for 4 hours and collected for WB for TβRII. The TβRII protein levels affected by TUFT1 knockdown were restored by MβCD in LX2 cells. **P < 0.01, ***P < 0.001 by ANOVA, n = 3. **B** LX2 cells with knockdown of TUFT1

or knockdown of both TUFT1 and caveolin-1 (CAV1) were subjected to WB for TβRII. The TβRII protein levels affected by TUFT1 knockdown were restored by CAV1 knockdown. **P < 0.01, ***P < 0.001 by ANOVA, n = 3. **C, D** qRT-PCR and WB revealed that mRNA and protein levels of CAV1 were not affected by TUFT1 knockdown in LX2 cells. Not significant (n.s., P > 0.05) by ANOVA, n = 3. **E** Double IF staining for TUFT1 (red) and EEA1 (green) showed that the rate of TUFT1/EEA1 co-localization (yellow, arrows) was higher in CAV1 knockdown HSCs compared to control HSCs. Arrows highlight areas of co-localization. Scale bar, 20 μm. ***P < 0.001 by t-test, n = 12.

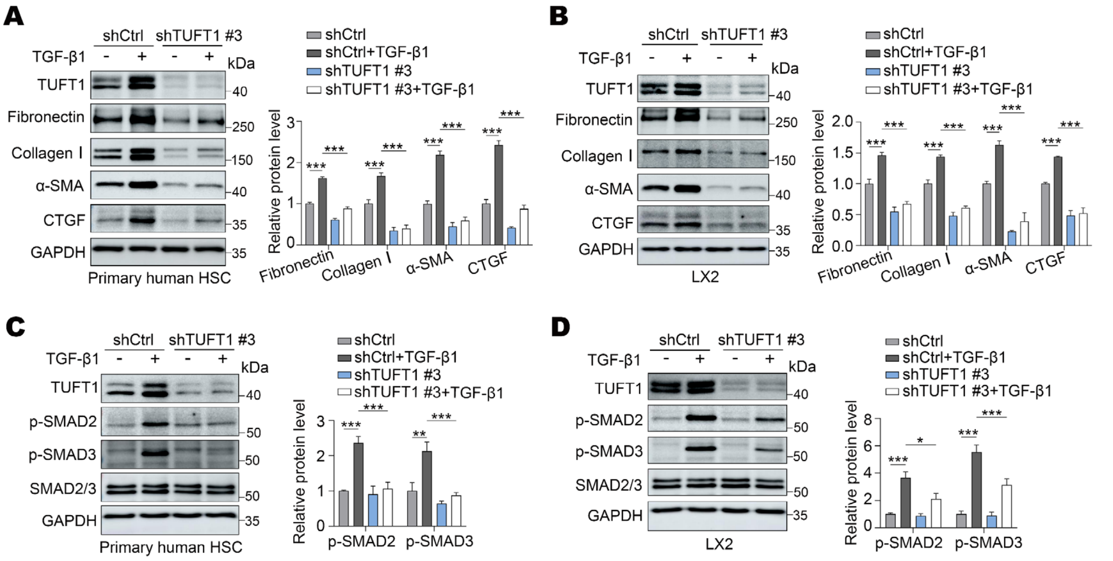

Fig. S8 TUFT1 knockdown inhibits TGF-β1–induced activation of HSCs into myofibroblasts. **A** Primary human HSCs and **B** LX2 cells transduced with shTUFT1 #3 lentiviruses were incubated with TGF-β1 (5 ng/mL) for 24 hours, and WB was performed to detect cell expression of activation markers. TGF-β1-mediated HSC activation was suppressed by TUFT1 knockdown. ***P < 0.001 by ANOVA, n = 3. **C**

Primary human HSCs and **D** LX2 cells transduced with shTUFT1 #3 lentiviruses were incubated with TGF-β1 (5 ng/mL) for 30 minutes, and WB was performed to detect phosphorylation of SMAD2 and SMAD3 induced by TGF-β1 in the cells. TGF-β1-induced SMAD2/3 phosphorylation was suppressed by TUFT1 knockdown. *$P < 0.05$, **$P < 0.01$, ***$P < 0.001$ by ANOVA, $n = 3$.

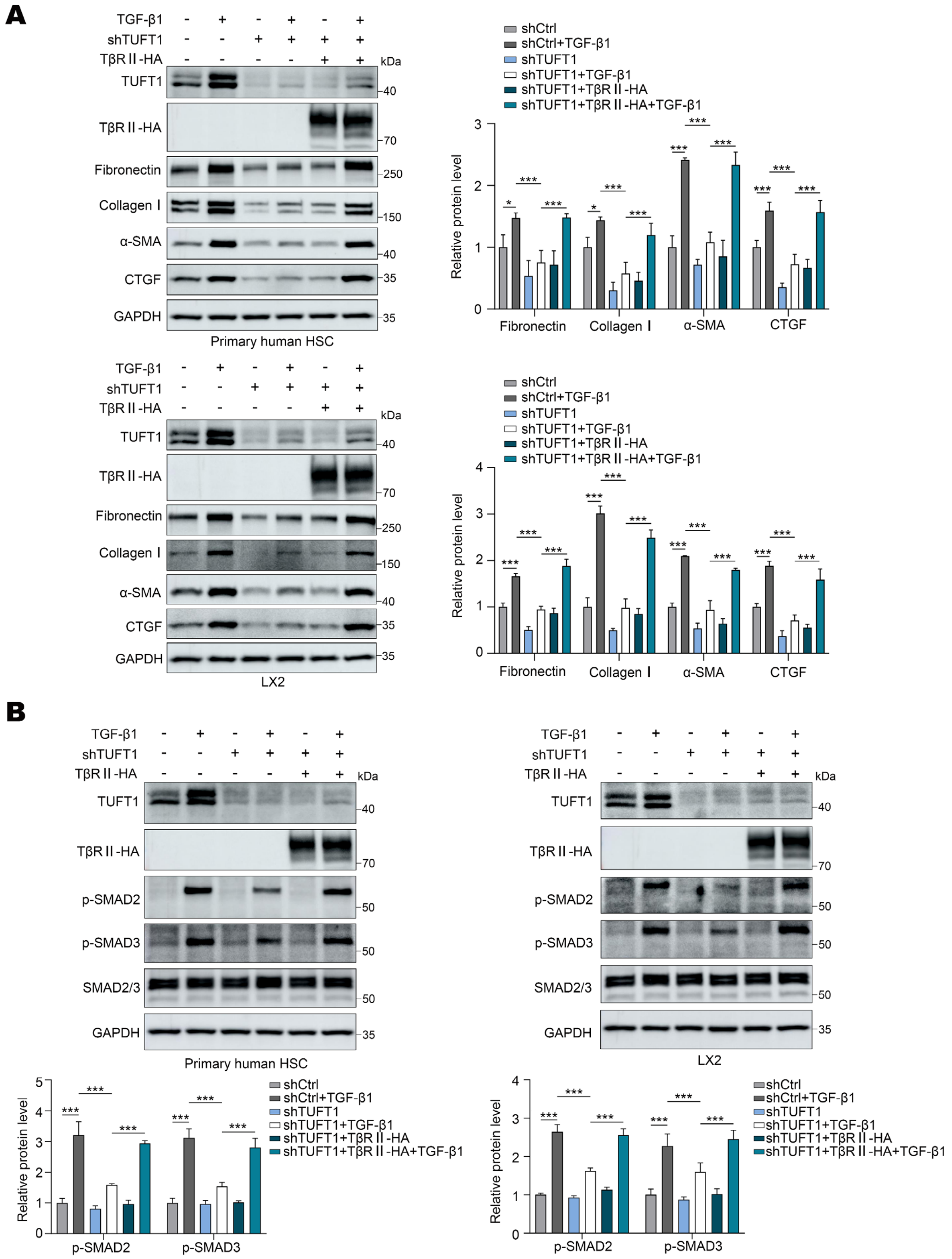

Fig. S9 TUFT1 promotion of HSC activation into myofibroblasts induced by TGF-β1 is dependent on TβRII. A TUFT1 knockdown primary human HSCs (upper) and TUFT1 knockdown LX2 cells (lower) were transduced with control or TβRII-HA lentiviruses, followed by TGF-β1 stimulation (5 ng/mL) for 24 hours. WB revealed that

the impaired expression of stellate cell activation markers in TGFβ1-stimulated TUFT1 knockdown cells was rescued by TβRII-HA overexpression. *P < 0.05, ***P < 0.001 by ANOVA, n = 3. **B** The cells as described in **A** were incubated with TGF-β1 (5 ng/mL) for 30 minutes. WB revealed that the impaired phosphorylation of SMAD2 and SMAD3 in TGFβ1-stimulated TUFT1 knockdown cells was rescued by TβRII-HA overexpression. ***P < 0.001 by ANOVA, n = 3.

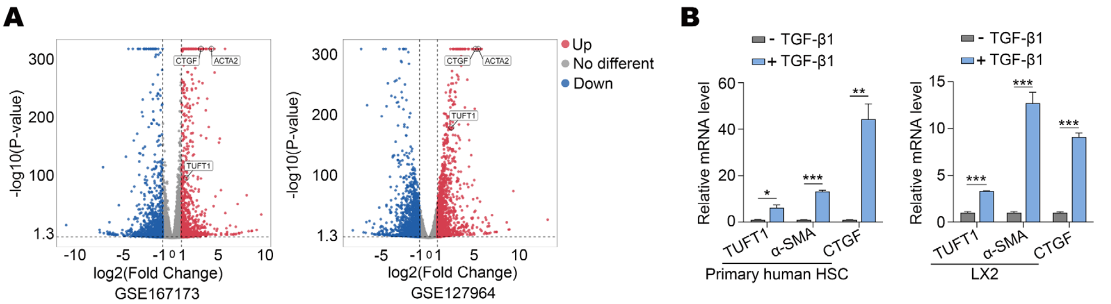

Fig. S10 TUFT1 expression is induced by the TGF-β/SMAD signaling in HSCs. A Volcano plots for the GSE167173 and GSE127964 datasets revealed that TUFT1 transcripts were upregulated by TGF-β1 stimulation in primary human HSCs. **B** qRT-PCR confirmed upregulation of TUFT1 transcripts by TGF-β1 stimulation for 24 hours (5 ng/mL) in primary human HSCs (left) and LX2 cells (right). *P < 0.05, **P < 0.01, ***P < 0.001 by t-test, n = 3.

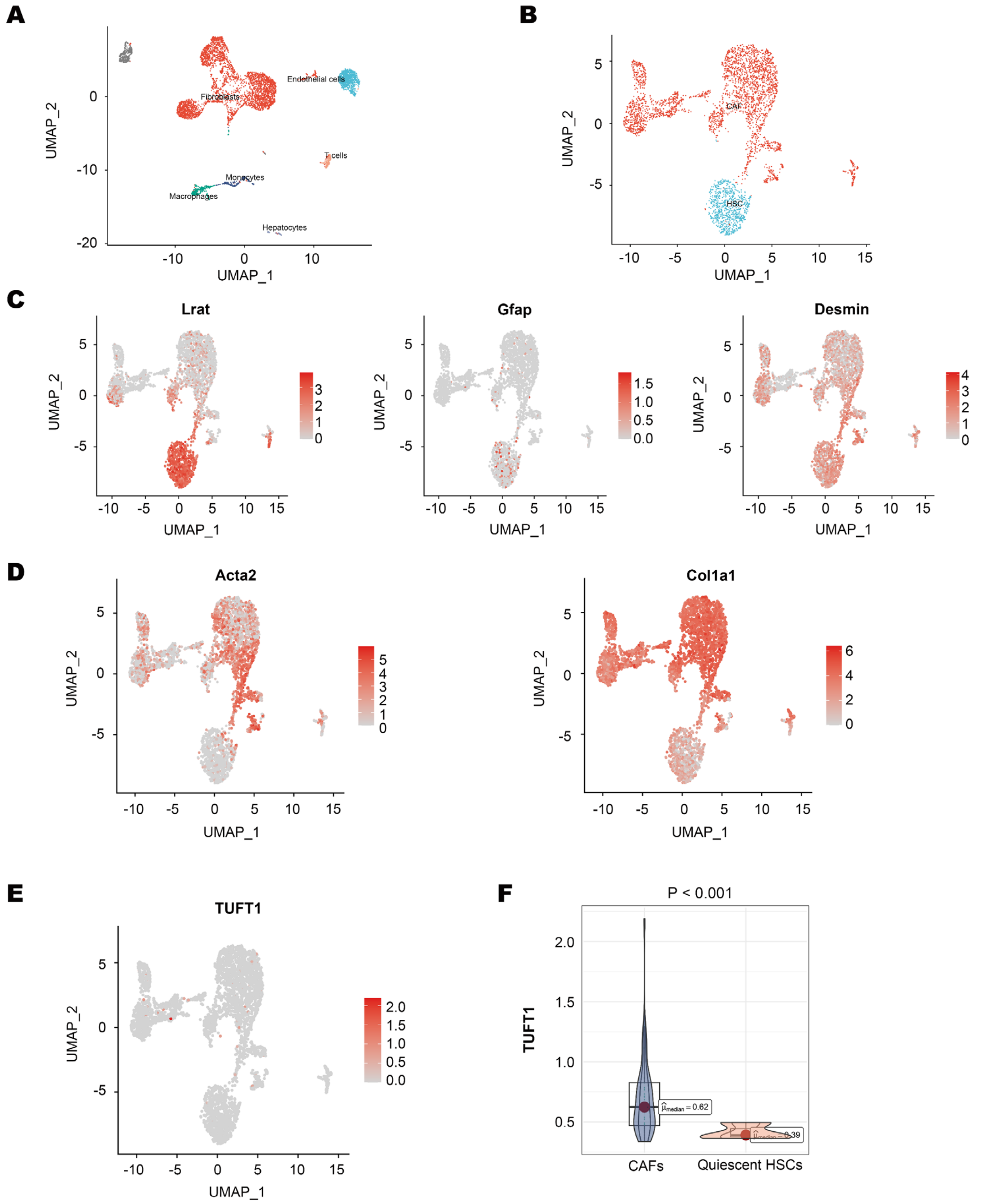

Fig. S11 Single-cell RNA sequencing revealing higher number of *Tuft1* transcripts in the CAFs of CMT93 murine CRC liver metastases compared to quiescent HSCs. **A** GSM4874984 datasets of GSE160541 in GEO were analyzed by the R toolkit Seurat. The UMAP revealed that the cells were divided into 6 clusters based on their gene expression pattern. **B** The cells of the fibroblast group were further divided into 2 clusters. **C** UMAPs revealing cells expressing the transcripts of *Lrat*, *Gfap*, or *Des*. The

redder the color is, the higher the transcript level is. **D** UMAP plots showing cells expressing Acta2 or Col1a1 transcripts. The redder the color is, the higher the transcript level is. **E** An UMAP revealing cells expressing *Tuft1* transcripts. The redder the color is, the higher the transcript level is. **F** Quantification of *Tuft1* transcripts at the single-cell level revealed a higher number of *Tuft1* transcripts in the CAFs compared to quiescent HSCs. P < 0.001, the Wilcoxon signed rank was used to compare the two groups.

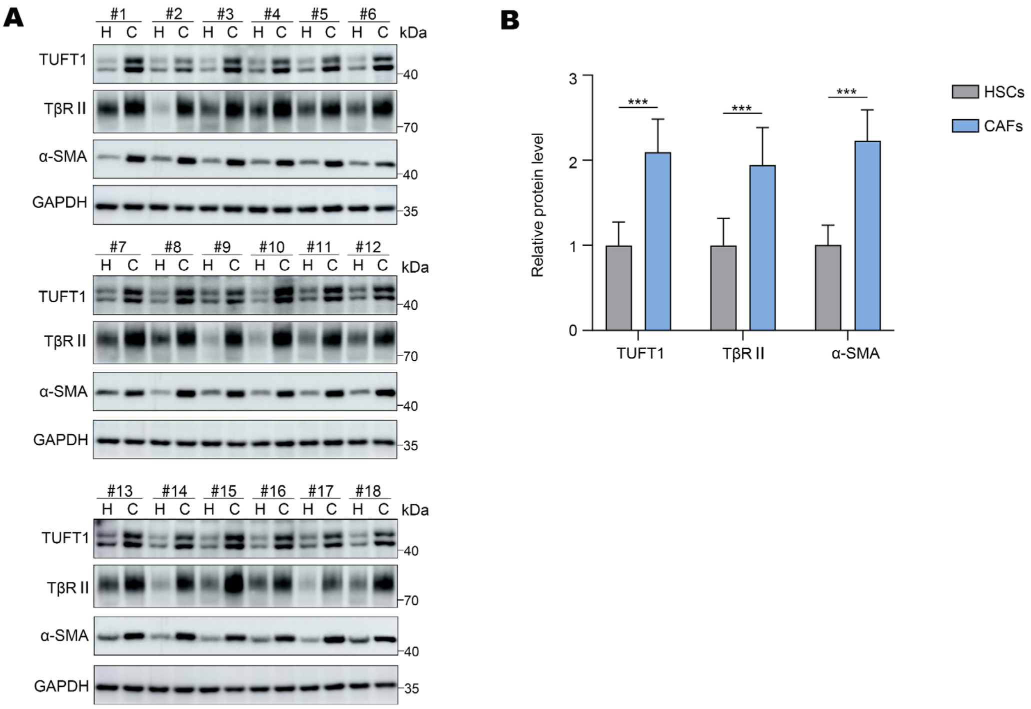

Fig. S12 Higher TUFT1 and TβRII protein levels were detected in the CAFs of patient CRCLM than in adjacent liver HSCs. **A** CAFs of CRCLM and matched HSCs in the adjacent normal liver tissues were isolated from 18 CRC patients. Cells were cultured *in vitro* and collected for WB. As isolated HSCs underwent spontaneous activation during *in vitro* culture, low levels of α-SMA were detected in HSCs by WB.

B Densitometric analysis revealed that the CAFs of patient CRCLM express higher TUFT1 and TβRII protein levels than matched HSCs. ***P < 0.001 by t-test, n = 18.

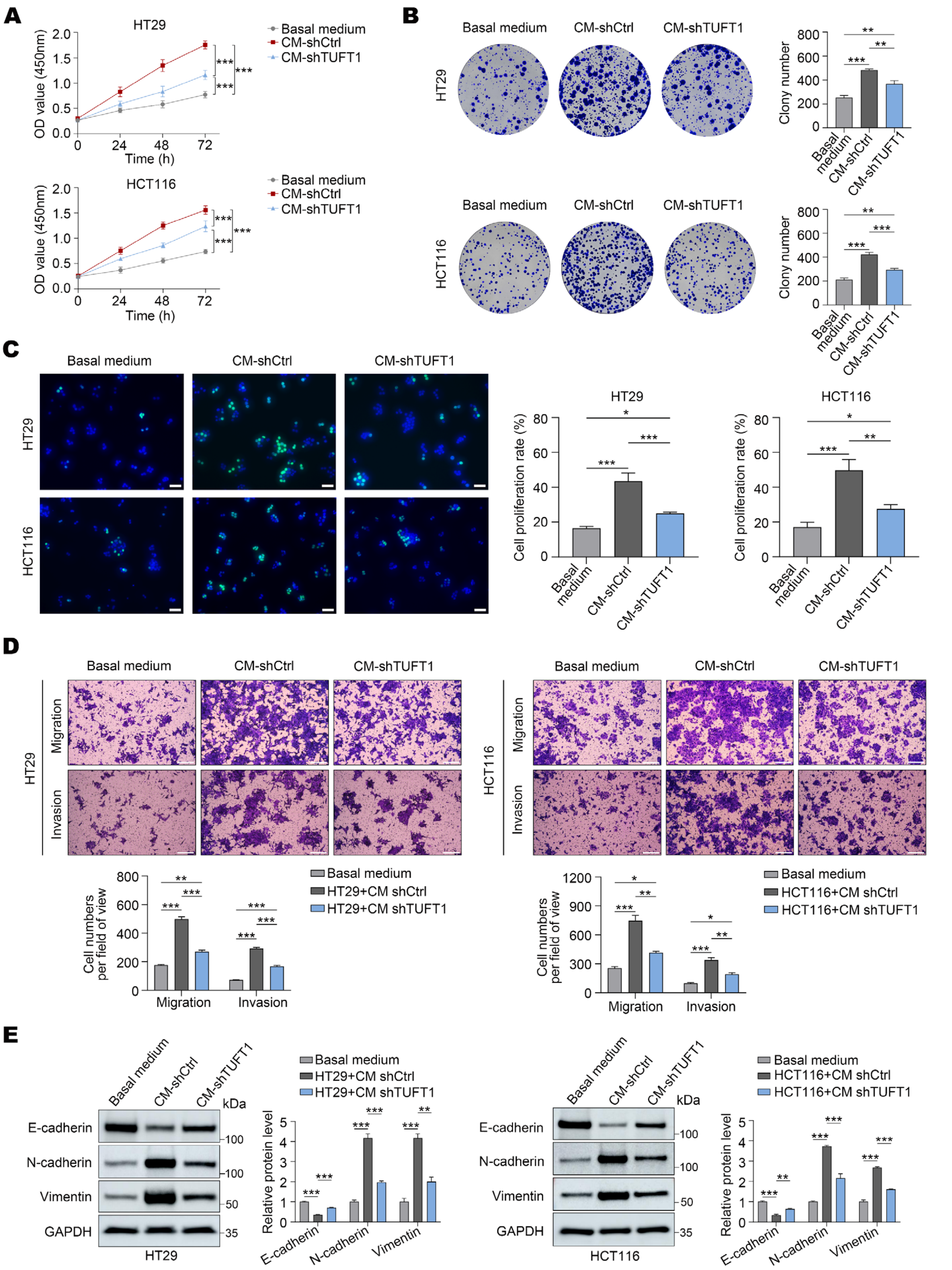

Fig. S13 TUFT1 knockdown suppresses the tumor-promoting effect of HSCs *in*

***vitro*. A** CCK8 assay results for HT29 and HCT116 cells incubated with conditioned medium (CM)-shCtrl or CM-shTUFT1. ***P < 0.001 by ANOVA, n = 3. **B** Colony formation assay results for HT29 and HCT116 cells incubated with CM-shCtrl or CM-shTUFT1. **P < 0.01, ***P < 0.001 by ANOVA, n = 3. **C** Edu staining showed that the rate of HT29 and HCT116 cells at the S-phase is higher in cells incubated with CM-shCtrl than in cells incubated with CM-shTUFT1. Scale bar, 50 μm. *P < 0.05, **P < 0.01, ***P < 0.001 by ANOVA, n = 3. **D** Transwell assay detected reduced effect of CM-shTUFT1 on the migration and invasion of HT29 and HCT116 cells compared to CM-shCtrl. Scale bar, 150 μm. *P < 0.05, **P < 0.01, ***P < 0.001 by ANOVA, n = 3. **E** WB revealed differential regulation of the expression of epithelial-to-mesenchymal transition (EMT) markers by HT29 and HCT116 cells incubated with CM-shCtrl or CM-shTUFT1. **P < 0.01, ***P < 0.001 by ANOVA, n = 3.

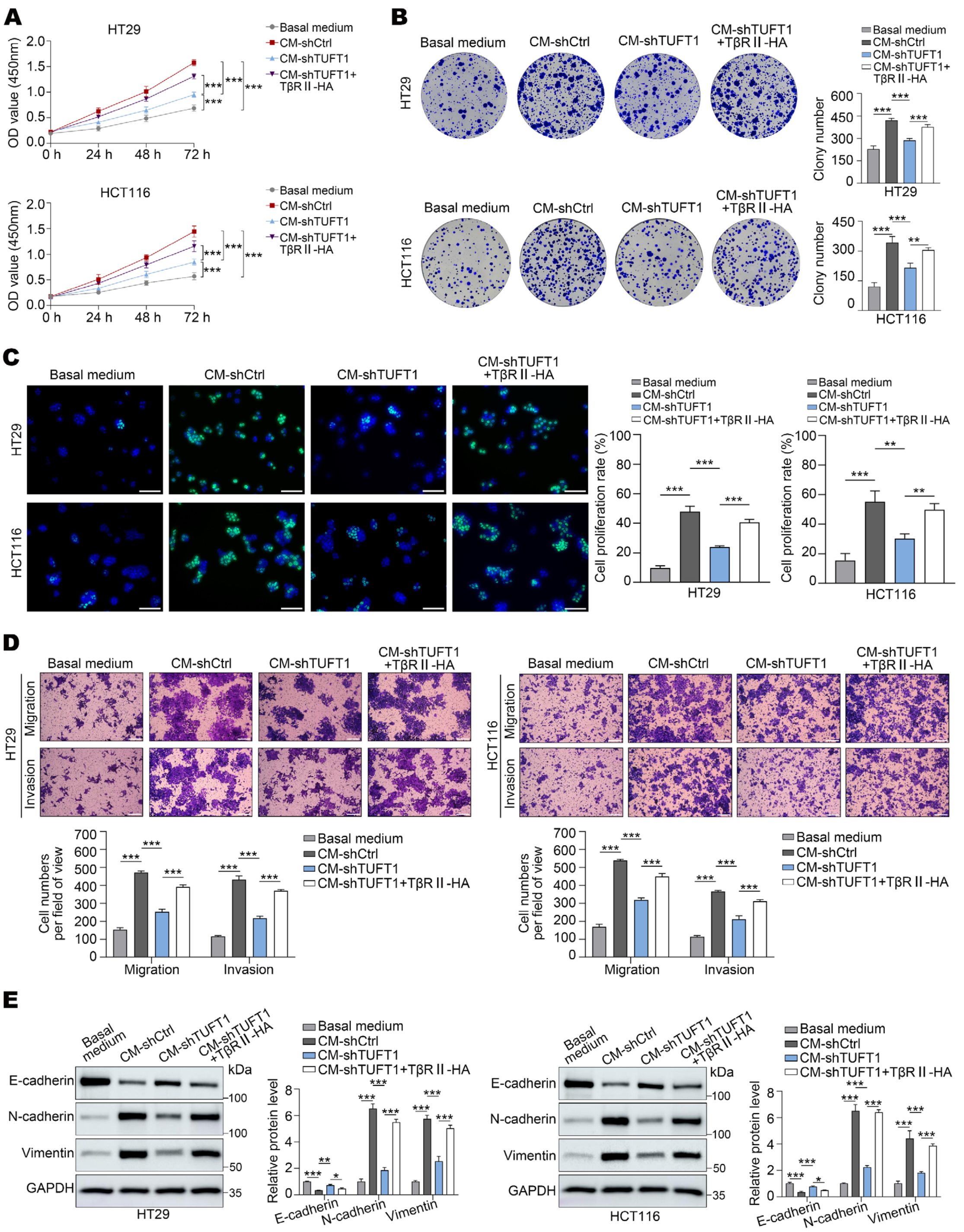

Fig. S14 TUFT1 contributes to the tumor-promoting effect of HSCs depending on TβRII in vitro. A HT29 and HCT116 cells were incubated with CM-shCtrl, CM-shTUFT1, or CM-shTUFT1+TβRII-HA, and cell proliferation was detected by CCK-8 assay. ***P < 0.001 by ANOVA, n = 3. **B** Colony formation ability was assessed for HT29 and HCT116 cells incubated with CM-shCtrl, CM-shTUFT1, or CM-

shTUFT1+TβRII-HA. **P < 0.01, ***P < 0.001 by ANOVA, n = 3. **C** The results of Edu staining of HT29 and HCT116 cells stimulated with CM-shCtrl, CM-shTUFT1, or CM-shTUFT1+TβRII-HA. Scale bar, 100 μm. **P < 0.01, ***P < 0.001 by ANOVA, n = 3. **D** Transwell assays revealed that the effect of CM-shTUFT1 on the migration and invasion of HT29 and HCT116 cells was suppressed compared with CM-shCtrl, which was reversed by TβRII-HA overexpression in TUFT1 knockdown cells. Scale bar, 150 μm. P < 0.001 by ANOVA, n = 3. **E** WB analysis of EMT markers expressed by CRC cells incubated with CM-shCtrl, CM-shTUFT1, or CM-shTUFT1+TβRII-HA. *P < 0.05, **P < 0.01, ***P < 0.001 by ANOVA, n = 3.

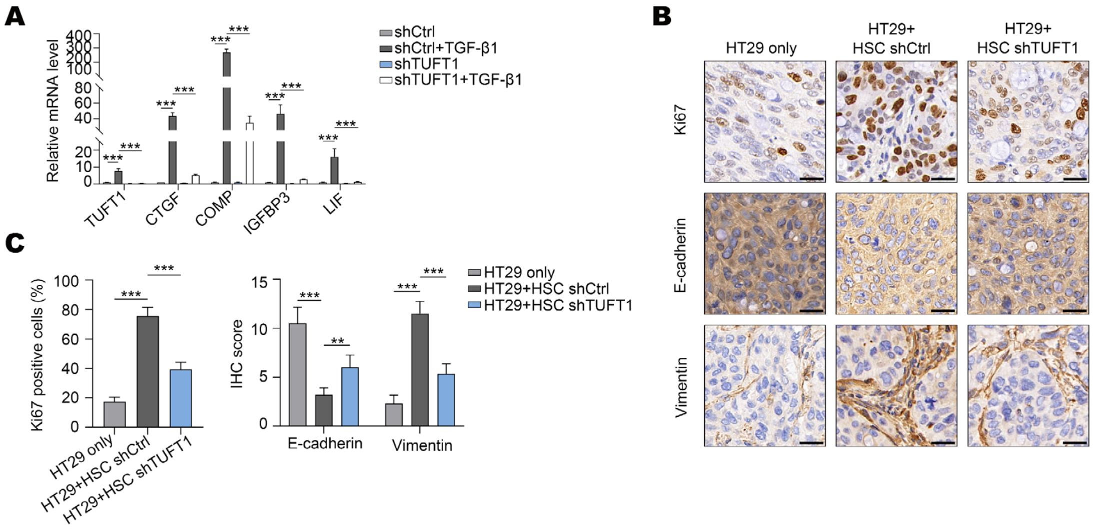

Fig. S15 TUFT1 knockdown suppresses the tumor-promoting effect of HSCs in mice. A qRT-PCR validated that the expression of 4 tumor-promoting factors by primary human HSCs was induced by TGFβ1 in a TUFT1-dependent manner. ***P < 0.001 by ANOVA, n = 3. **B** Pictures of IHC for Ki67, E-cadherin, and Vimentin on HT29 subcutaneous xenografts are shown. Scale bar, 25 μm. **C** Percentages of Ki67-positive cells and IHC scores of E-cadherin and Vimentin in different groups are shown.

**P < 0.01, ***P < 0.001 by ANOVA, n = 6.

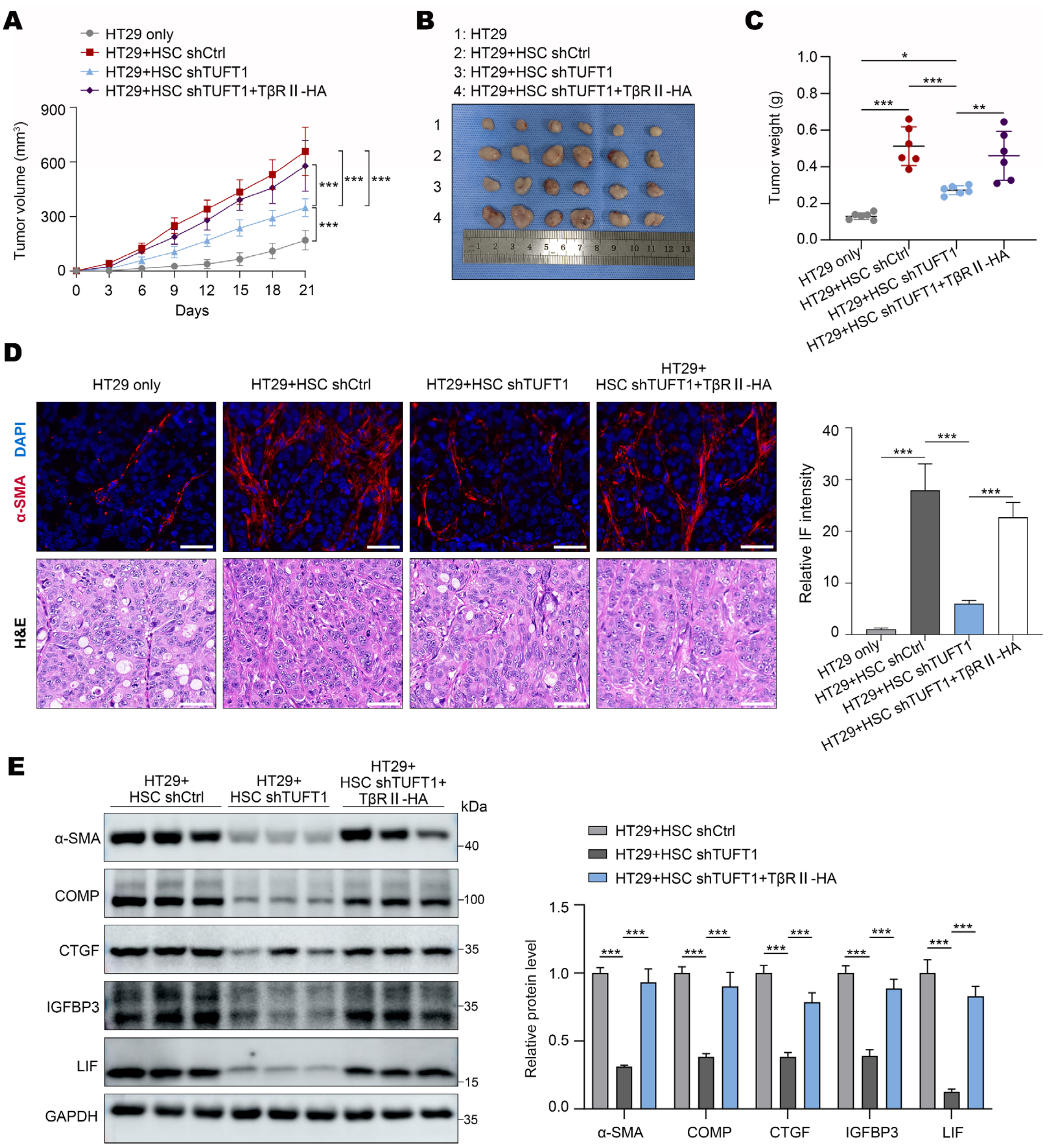

Fig. S16 TUFT1 promotes the tumor-promoting effect of HSCs in mice via TβRII.

A, B, C Subcutaneous HT29/HSCs co-injection into nude mice revealed that knocking down TUFT1 of HSCs reduced the tumor-promoting effect of HSCs, which was partially rescued by TβRII-HA overexpression. Tumor growth curves are shown in (**A**); the pictures of HT29 tumors at the endpoint are shown in (**B**), and tumor weights are shown in (**C**). *P < 0.05, **P < 0.01, ***P < 0.001 by ANOVA, n = 6. **D** IF staining

of α-SMA showed that the reduced CAF densities in tumors arising from HT29+HSC-shTUFT1 co-injections were partially restored in tumors arising from HT29+HSC-shTUFT1+TβRII-HA co-injections. Scale bar, 50 μm. ***P < 0.001 by ANOVA, n = 6. **E** The levels of HSC-derived tumor-promoting factors in tumors arising from HT29+HSC-shTUFT1 co-injections were partially restored in tumors arising from HT29+HSC-shTUFT1+TβRII-HA co-injections. ***P < 0.001 by ANOVA, n = 6.

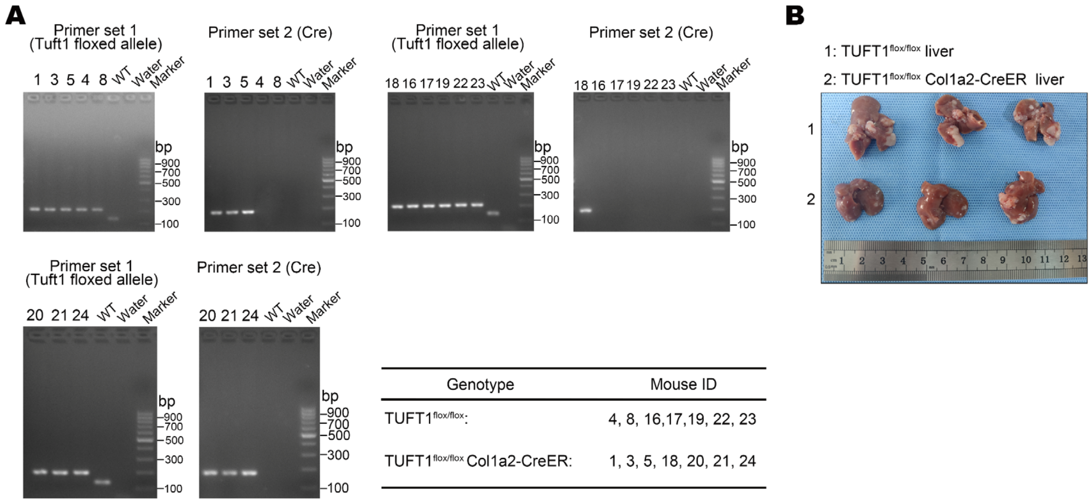

Fig. S17 Targeting HSC TUFT1 by Cre/LoxP recombination suppresses CRCLM in mice. A PCR genotyping results showed 7 Cre^{+} and 7 Cre^{-} mice that were homozygotes for the Tuft1 floxed alleles. The primer set 1 was used to detect the Tuft1 floxed allele; the primer set 2 was used to detect the Cre transgene. **B** Images of the murine liver after portal vein injection are shown.
